# Modular Synthesis of Benzoylpyridines Exploiting a Reductive Arylation Strategy

**DOI:** 10.1021/acs.orglett.3c03833

**Published:** 2023-12-22

**Authors:** Antonella Ilenia Alfano, Megan Smyth, Scott Wharry, Thomas S. Moody, Marcus Baumann

**Affiliations:** † School of Chemistry, University College Dublin, Science Centre South, Dublin 4, Ireland; ‡ Technology Department, Almac Sciences, Craigavon BT63 5QD, United Kingdom; § Arran Chemical Company, Monksland Industrial Estate, Roscommon N37 DN24, Ireland

## Abstract

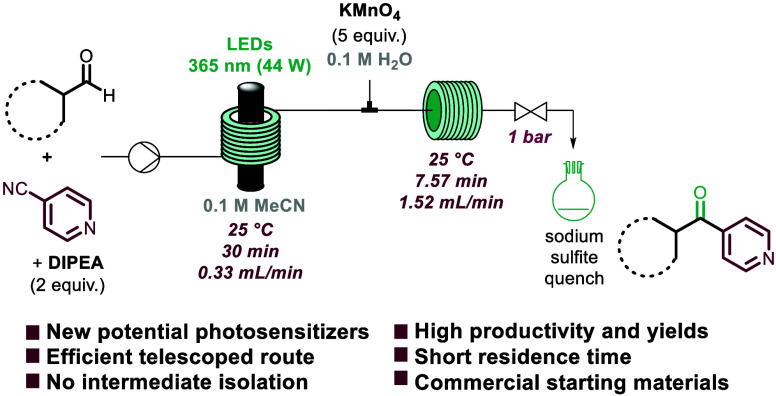

Herein we disclose a telescoped flow strategy to access electronically differentiated bisaryl ketones as potentially new and tunable photosensitizers containing both electron-rich benzene systems and electron-deficient pyridyl moieties. Our approach merges a light-driven (365 nm) and catalyst-free reductive arylation between aromatic aldehydes and cyanopyridines with a subsequent oxidation process. The addition of electron-donating and withdrawing substituents on the scaffold allowed effective modification of the absorbance of these compounds in the UV–vis region, while the continuous flow process affords high yields, short residence time, and high throughput.

Recent years have witnessed a renaissance of photochemical reactions driven by the desire for milder and more selective chemical transformations.^1^ The sustainability of chemical reactions can also be improved by using light because photons act as “traceless” reagent equivalents^2^ whose energy can be tuned based on their wavelength. However, additional considerations, including solvent choice, concentration, and reaction time, play an important role in this context.

Organic molecules tend to absorb light in the UV region of the electromagnetic spectrum (<360 nm) unless they are sufficiently conjugated. Thus, photocatalysts and photosensitizers are commonly required additives in photochemical reactions operating in the visible region. Developments in modern photocatalysis thereby have highlighted the power of Ru- and Ir-based catalysts to bring about a plethora of valuable synthetic processes using visible light.^3^ While these features are among the key drivers that account for the increasing popularity of photochemical reactions, the high cost and potential toxicity of these precious metal catalysts hinder their uptake in industrial settings. In this context, simple organic molecules with sufficient conjugation continue to play a key role in scaled photochemical reactions, and triplet photosensitizers such benzophenone and (thio)xanthone are frequently used examples.^4^ While these entities are readily available at low cost, display good solubility across various organic solvents, and are considered nonharmful, the introduction of electron-donating or -withdrawing substituents that is critical to modify their photophysical properties commonly necessitates long and inefficient synthesis routes. An important report by Elliott, Booker-Milburn, and co-workers showcases the impact of such substituent alterations on the absorbance maximum (i.e., λ_max_) and thus product selectivity of a series of thioxanthone derivatives.^5^

To address this challenge, we set out to create an expedited route into electronically differentiated bisaryl ketones that combine electron-rich benzene systems with electron-deficient pyridyl moieties. Continuous flow processing was thereby employed from the outset as a technology to provide increased scalability, reaction efficiency, and reproducibility.^6^

As shown in Scheme 1, our strategy combines a reductive arylation reaction between aryl aldehydes and cyanopyridines with an oxidation of the resulting secondary alcohol products to yield the desired bisaryl ketones via a modular route. We envisioned exploiting photochemical processing for the reductive arylation stage. Precedent by Wu^7^ and Xia^8^ highlighted that a variety of aryl reaction partners can be coupled under photochemical conditions (blue LEDs, 5–72 h) when using Ir-based catalysts (e.g., *fac*-Ir(ppy)_3_, Ir(ppy)_2_(dtbbpy)PF_6_). Complementary electrochemical strategies were recently reported for this transformation by Xia^9^ and Findlater,^10^ but long reaction times (ca. 6 h), supporting electrolytes (*n*Bu_4_NBF_4_, *n*Bu_4_NOAc), and undesired solvents (e.g., DMF) were required, which is problematic in industrial environments. Very recently, Xue and co-workers^11^ developed a general catalyst-free photoinduced pathway to activate carbonyl compounds, generating a ketyl radical that can be trapped by several coupling partners.

**Scheme 1:**
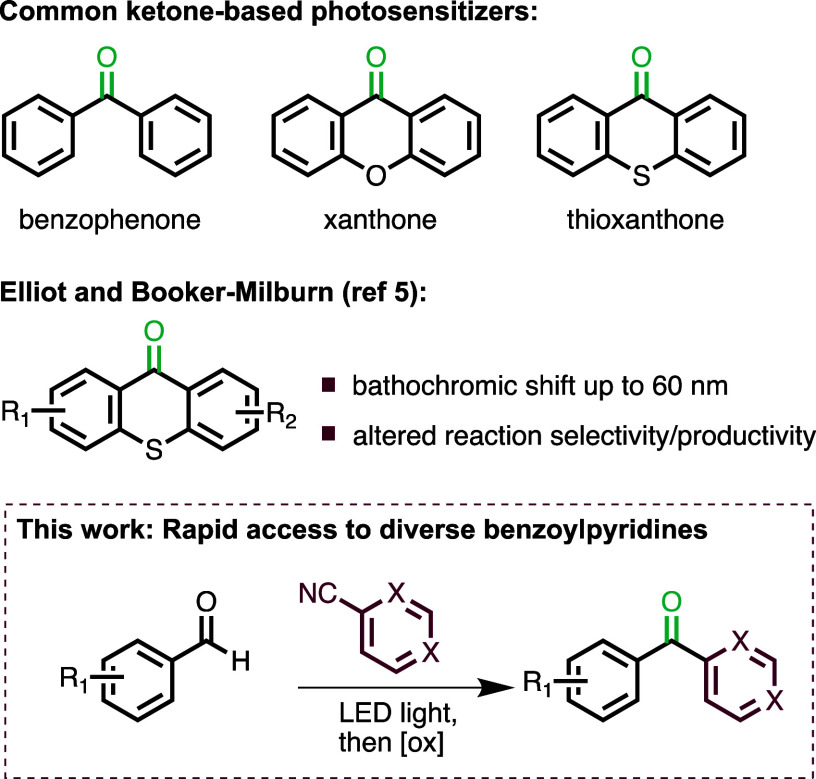
Common Sensitizers and Proposed Strategy

Additionally, numerous compounds bearing aryl-substituted 2- or 4-(pyridinyl)methanol scaffolds (as free alcohol or ether) are found among medicinally relevant entities such as histamine H1 antagonists,^12,13^ an HIV-1 NMRT inhibitor,^14^ an aldosterone synthase inhibitor,^15^ and an LTA4H inhibitor,^16^ (Figure 1) further demonstrating the value of a direct route to these entities.

**Figure 1:**
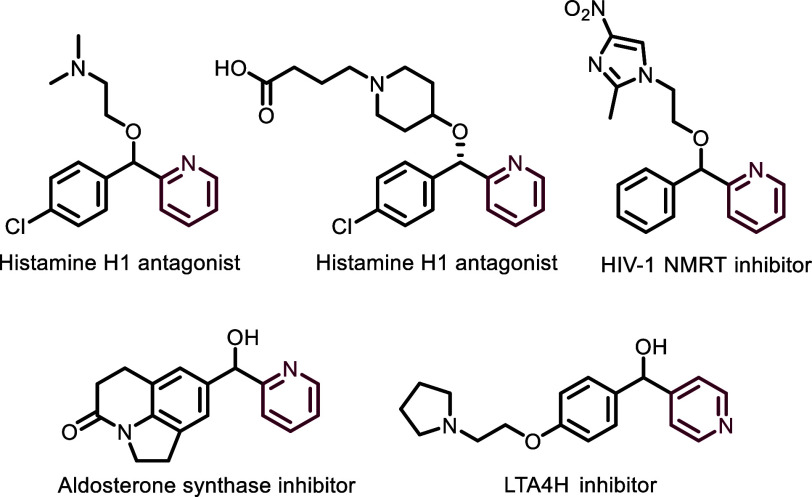
Representative medicinally relevant compounds containing the aryl(pyridinyl)methanol core.^12−16^

In our quest toward the development of robust flow conditions for the reductive arylation, we started the investigation by using readily available benzaldehyde and 4-cyanopyridine as model compounds to access the desired aryl(pyridinyl)methanol core. A thorough exploration of key parameters allowed the identification of the optimum residence time, stoichiometry, solvent, concentration, wavelength, and wattage. The flow setup consisted of a Vapourtec E-series reactor with its UV-150 photomodule equipped with different light sources (i.e., LEDs), an adjustable back-pressure regulator (BPR), and a tubular reactor coil (PFA, i.d. = 1/16 in., *V* = 10 mL).

As summarized in Table 1, optimal conditions were found using 2 equiv of 4-cyanopyridine as coupling partner, 2 equiv of DIPEA as single electron transfer (SET) agent, a substrate concentration of 0.1 M, light of 365 nm (44 W input power), and a residence time of 30 min (entry 1), affording a 95% yield of product **3a**. The reaction proceeded slowly under both lower input power and different wavelengths (entries 2 and 3). A shorter residence time of 15 min did not improve the results, as incomplete substrate conversion was observed (entry 4). The addition of water to MeCN did not affect the yield (entry 5), while the use of other solvents, such as 1,4-dioxane, caused a significant yield reduction of 50% (entry 6). Further attempts to improve the reaction throughput by increasing the substrate concentration (0.4 M) gave a lower yield (entry 7). The replacement of DIPEA with triethylamine (TEA) did not improve the results (entry 8). Control experiments demonstrated that light and the base are essential for the observed reactivity (entries 9 and 10).

**Table 1:**
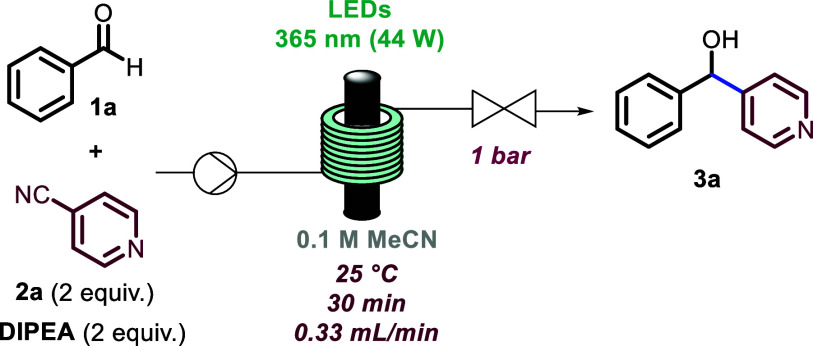
Optimization of the Reaction Conditions^a^

entry	deviations from the above conditions	yield of **3a** (%)^b^
1	none	95
2	365 nm (20 W)	43
3	385 nm (70 W)	34
4	15 min	71
5	MeCN/H_2_O (20:1) as solvent	90
6	1,4-dioxane as solvent	42
7	0.4 M	57
8	TEA as base	60
9	dark	–
10	no base	–

a Reaction conditions: **1a** (0.15 mmol), **2a** (2 equiv), DIPEA (2 equiv), MeCN (1.5 mL), Vapourtec reactor 44 W (λ = 365 nm, reactor volume = 10 mL), 25 °C, 30 min.

b Calculated by ^1^H NMR analysis using trichloroethylene as an internal standard.

Having established the functional setup for benzaldehyde as our model substrate, we also demonstrated the scalability of this reaction, performing two different long runs at different scales (1 and 7 mmol) with similar yields and reaching a throughput of 1.5 mmol/h. With these conditions in hand, we next embarked on investigating the reaction scope by using several aromatic aldehydes (Scheme 2).

**Scheme 2:**
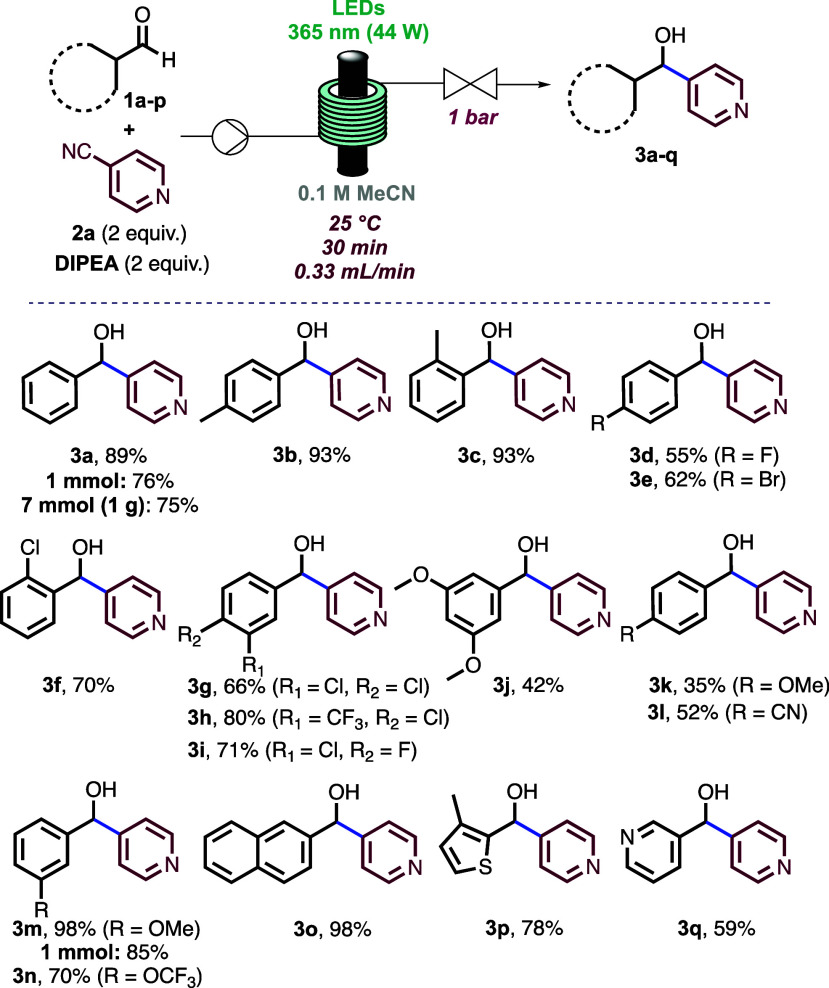
Scope of Aldehydes with 4-Cyanopyridine

First, *p*- and *o*-tolualdehyde afforded the corresponding alcohols (**3b** and **3c**) in excellent yields. Notably, our method is also amenable to halogen atoms present at the *para* and *ortho* positions, which are useful synthetic handles for further chemical transformations (**3d**–**3f**). Disubstituted aldehydes bearing halogens and trifluoromethyl groups gave the desired products in good yields (**3g**–**3i**). The reaction proceeded slowly with certain electron-donating substituents (**3j** and **3k**); however, the reversion of this trend was achieved when employing 3-methoxybenzaldehyde. The corresponding product (**3m**) was obtained in 98% yield, and the long run on a 1 mmol scale furnished similar results. The method tolerated the presence of other electron-withdrawing and neutral substituents (**3l**–**3o**) as well as heteroaryl aldehydes such as 4-methylthiophene-2-carboxaldehyde and 3-pyridinecarboxaldehyde (**3p** and **3q**).

Initially, when evaluating the scope of this process using 2-cyanopyridine as an acceptor, poor results were obtained due to incomplete substrate conversion and/or formation of 1,2-diphenylethane-1,2-diol (**6**) as a product of pinacol coupling of benzaldehyde (see the Supporting Information (SI) for details). When 2-cyanopyridine was used under the previously optimized conditions, a yield of 55% for product **4a** was achieved (Table 2, entry 1). A brief optimization study was undertaken to suppress the formation of side product **6**. Shorter or longer residence times of 10 and 50 min (entries 2 and 3) did not improve the results, due to low conversion or high yield of **6**, nor did increasing the equivalents of base (entry 4). However, the undesired pinacol pathway was almost entirely suppressed by the addition of methanol to the reaction mixture, the use of 3 equiv of 2-cyanopyridine, and performing the reaction under higher input power (entry 5).

**Table 2:**
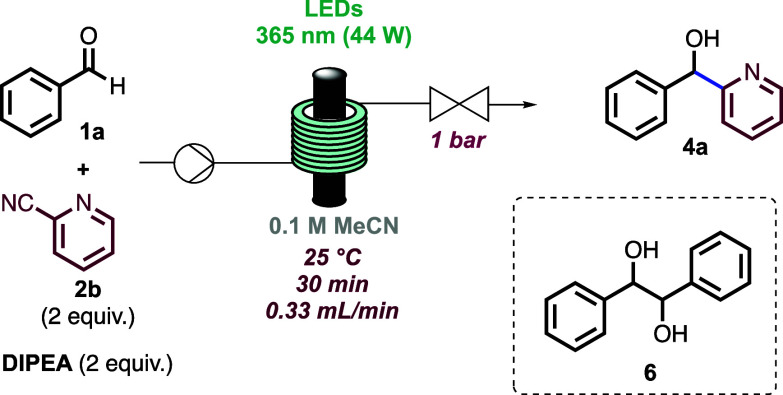
Reaction Optimization Using 2-Cyanopyridine

entry	deviations from above conditions	yield of **4a** (%)^a^
1	none	55
2	10 min	37
3	50 min	43
4	3 equiv of DIPEA	47
5	3 equiv of **2b**, 6 equiv of MeOH, 66 W	65

a Calculated by ^1^H NMR analysis using trichloroethylene as an internal standard.

Next, a small set of different benzaldehydes were evaluated with 2-cyanopyridine as the reaction partner. *Para*- and *ortho*-substituted benzaldehydes served as suitable coupling partners, generating the ketyl radicals toward the corresponding alcohols in good yields (Scheme 3, **4b**–**4d**). Using other cyanopyridines or pyrimidines as coupling partners was not well-tolerated (see the SI for details).

**Scheme 3:**
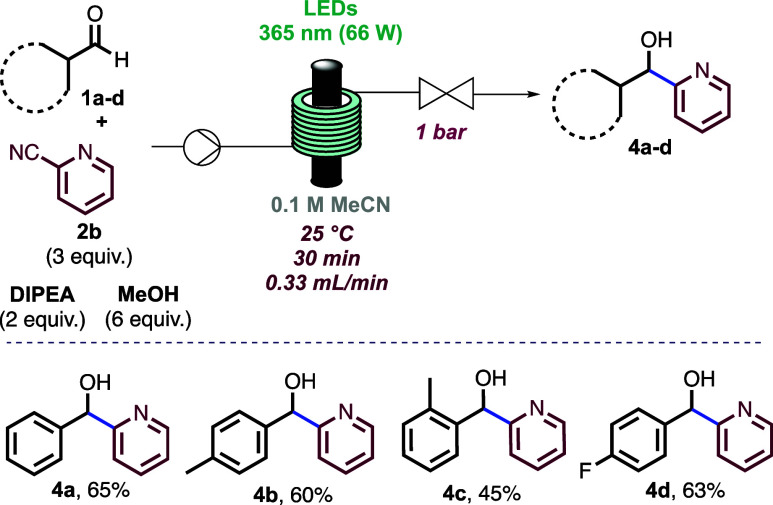
Scope of Aldehydes with 2-Cyanopyridine

Based on recent literature,^11^ a mechanism is proposed as shown in Scheme 4. Upon absorption of light (365 nm), the mixture of aromatic aldehyde and DIPEA furnishes the crucial ketyl radical intermediate **V** through SET and proton transfer pathways. Concomitant reduction of cyanopyridine **2** generates the radical anion **VI** through SET reduction, which undergoes radical–radical cross-coupling with **V** to give anion **VII**. Based on literature,^17^ the last step involves the elimination of the nitrile anion, which is quenched by reaction with the oxidized form of DIPEA to provide the final compound **3**.

**Scheme 4:**
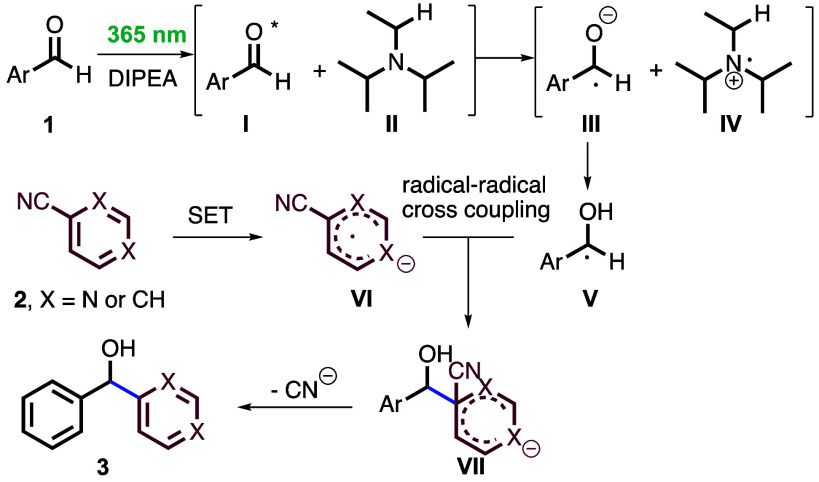
Proposed Mechanism

Guided by the importance of benzophenone and (thio)xanthone as photosensitizers,^4^ we decided to explore the use of the secondary alcohol products as substrates for a subsequent oxidation process yielding analogous carbonyl products. The introduction of a pyridine moiety adjacent to an aromatic ketone was already explored by Murafuji and co-workers^17^ in two different reactions, which highlighted the improved performance of unsubstituted benzoylpyridine compared to benzophenone. Capitalizing on the initial photochemical step, we sought to investigate a telescoped flow approach that avoided intermediate isolation and purification. Upon exiting the photochemical reactor, the reaction mixture containing the alcohol product **3** was then combined with a second stream containing potassium permanganate in water (0.1 M, 5 equiv).^18^ The combined stream was then directed into a second reactor coil (PFA, i.d. = 1/16 in., *V* = 10 mL) where the oxidation took place within a short residence time of 7.6 min (see the SI for details). The crude product was collected in a flask containing a quench solution (10% sodium sulfite). Using this telescoped approach, a small library of five target compounds was synthesized, exploring electron-donating and -withdrawing groups, in good to excellent yields (**5a**–**5e**, Scheme 5). As part of this study, we also demonstrated the stability of compound **5a** under photochemical conditions (365 nm, 50 W, 30 min exposure), recovering the ketone in stoichiometric amounts. To the best of our knowledge, this synthetic approach represents the first flow protocol for the rapid and easily scalable assembly of arylbenzoylpyridine cores bearing electron-donating or -withdrawing substituents.

**Scheme 5:**
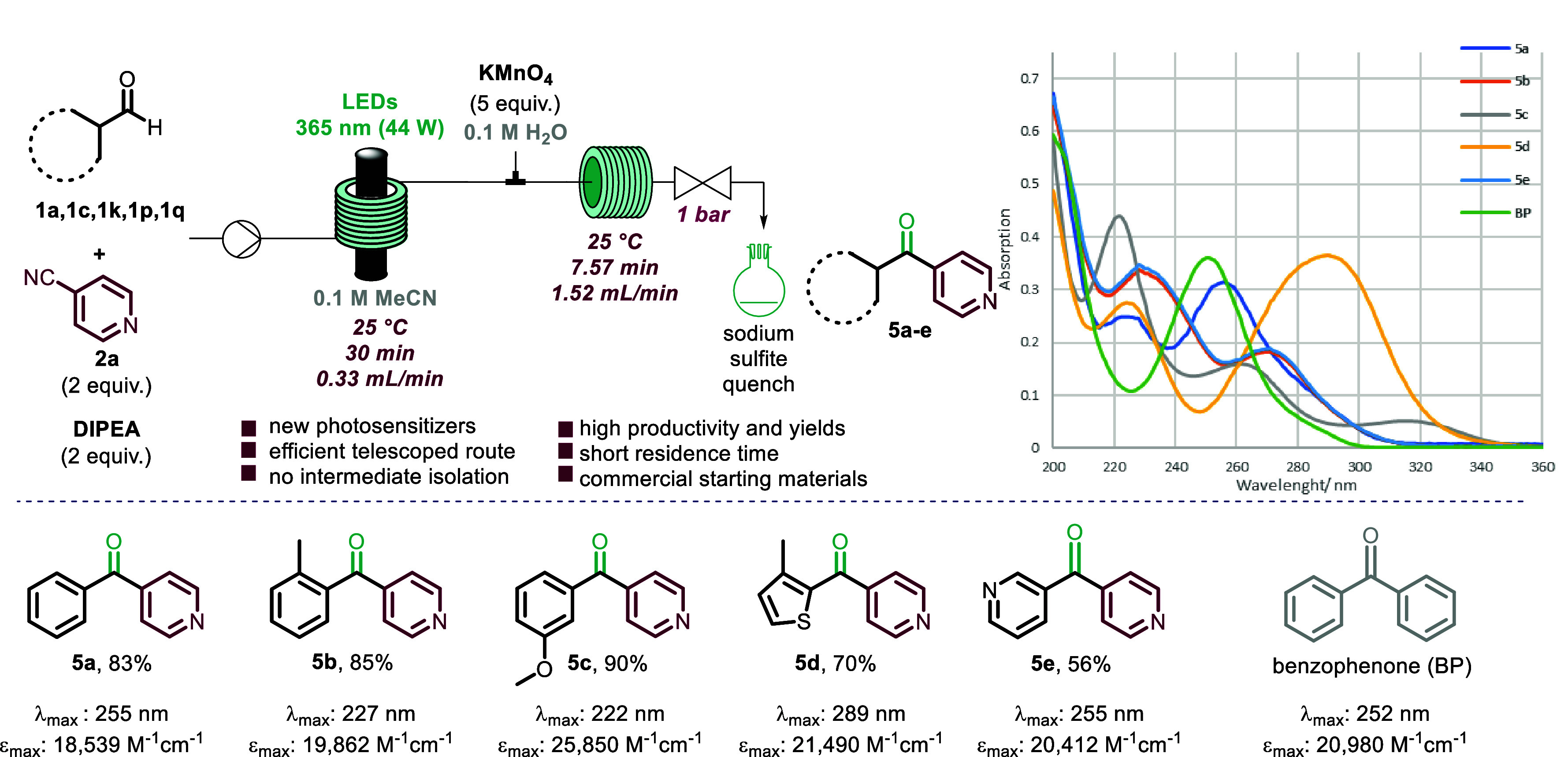
Telescoped Approach for the Synthesis of Bisaryl Ketones

Next, UV–vis absorption spectra for the ketone products were recorded at a concentration of 0.017 mM in acetonitrile. Data for compounds **5a**–**5e** and benzophenone are represented in composite spectra for comparison. In line with the literature,^19^ benzophenone strongly absorbed light within the UV region (λ_max_ = 252 nm) and showed a maximum molar absorption coefficient of around 21,000 M^–1^ cm^–1^. Compound **5a** presented a slight shift (λ_max_ = 255 nm) compared to the parent benzophenone, while the effect is more pronounced for thiophene derivative **5d** (λ_max_ = 290 nm), as indicated by a shift of +38 nm. Substituents at the 2- or 3-position of the benzene ring induced a blue shift (**5b**, λ_max_ = 227 nm; **5c**, λ_max_ = 222 nm), which is similar for the bispyridyl species **5e** (λ_max_ = 227 nm). The λ_max_ values of all ketone products are effectively distributed across the UV-B/C range. All compounds showed excellent absorption properties, having maximum molar absorption coefficients at the maximum absorption wavelength (ε_max_) similar to that of benzophenone.

In conclusion, we report an efficient flow approach to access electronically differentiated benzoylpyridines from cheap and readily available starting materials. The first step features an attractive catalyst-free reductive arylation process under photochemical conditions, followed by a telescoped oxidation using KMnO_4_ as a benign oxidant. The utility of this methodology was demonstrated by coupling a variety of aromatic and heteroaromatic aldehydes with different cyanopyridines in a continuous flow process that is characterized by high efficiency as well as reproducibility. The resulting flow sequence enables a straightforward entry into sets of new photosensitizers benefiting from high throughput, high yields, and short residence time, which render this approach suitable for further industrial development.

## Data Availability

The data underlying this study are available in the published article and its Supporting Information.

## Supporting Information Available

Complete experimental procedures and compound characterization (PDF)
FAIR data, including the primary NMR FID files, for compounds **3a**–**3q**, **4a**–**4d**, and **5a**–**5e** (ZIP)

## References

[ref1] aHoffmann N. Photochemical Reactions as Key Steps in Organic Synthesis. Chem. Rev. 2008, 108, 1052–1103. 10.1021/cr0680336. 18302419

[ref2] aBonfield H. E.; Knauber T.; Lévesque F.; Moschetta E. G.; Susanne F.; Edwards L. J. Photons as a 21st century reagent. Nat. Commun. 2020, 11, 804 10.1038/s41467-019-13988-4. 32029723 PMC7004975

[ref3] aYoon T. P.; Ischay M. A.; Du J. Visible Light Photocatalysis as Greener Approach to Photochemical Synthesis. Nat. Chem. 2010, 2, 527–32. 10.1038/nchem.687. 20571569

[ref4] aBeckett A.; Porter G. Primary photochemical processes in aromatic molecules. Trans. Faraday Soc. 1963, 59, 2038–2050. 10.1039/TF9635902038.

[ref5] Elliott L. D.; Kayal S.; George M. W.; Booker-Milburn K. Rational Design of Triplet Sensitizers for the Transfer of Excited State Photochemistry from UV to Visible. J. Am. Chem. Soc. 2020, 142, 14947–14956. 10.1021/jacs.0c05069. 32786778

[ref6] aBuglioni L.; Raymenants F.; Slattery A.; Zondag S. T. A.; Noël T. Technological Innovations in Photochemistry for Organic Synthesis: Flow Chemistry, High-Throughput Experimentation, Scale-up, and Photoelectrochemistry. Chem. Rev. 2022, 122, 2752–2906. 10.1021/acs.chemrev.1c00332. 34375082 PMC8796205

[ref7] Liu Z.; Nan X.; Lei T.; Zhou C.; Wang Y.; Liu W.; Chen B.; Tung C.; Wu L. Photo induced reductive cross coupling of aldehydes, ketones and imines with electron deficient arenes to construct aryl substituted alcohols and amines. Chin. J. Catal. 2018, 39, 487–494. 10.1016/S1872-2067(17)62896-1.

[ref8] Chen M.; Zhao X.; Yang C.; Xia W. Visible-Light-Triggered Directly Reductive Arylation of Carbonyl/Iminyl Derivatives through Photocatalytic PCET. Org. Lett. 2017, 19, 3807–3810. 10.1021/acs.orglett.7b01677. 28696124

[ref9] Zhang X.; Yang C.; Gao H.; Wang L.; Guo L.; Xia W. Reductive Arylation of Aliphatic and Aromatic Aldehydes with Cyanoarenes by Electrolysis for the Synthesis of Alcohols. Org. Lett. 2021, 23, 3472–3476. 10.1021/acs.orglett.1c00920. 33861088

[ref10] Zhang S.; Li L.; Li J.; Shi J.; Xu K.; Gao W.; Zong L.; Li G.; Findlater M. Electrochemical Arylation of Aldehydes, Ketones, and Alcohols: from Cathodic Reduction to Convergent Paired Electrolysis. Angew. Chem., Int. Ed. 2021, 60, 7275–7282. 10.1002/anie.202015230. 33377262

[ref11] Yan Y.; Li G.; Ma J.; Wang C.; Xiao J.; Xue D. Photoinduced generation of ketyl radicals and application in C-C coupling without external photocatalyst. Green Chem. 2023, 25, 4129–4136. 10.1039/D3GC00054K.

[ref12] Hite G.; Barouh V.; Dall H.; Patel D. Stereochemical aspects of antihistamine action. 4. Absolute configuration of carbinoxamine antipodes. J. Med. Chem. 1971, 14, 834–836. 10.1021/jm00291a014. 4400865

[ref13] Kida T.; Fujii A.; Sakai O.; Iemura M.; Atsumi I.; Wada T.; Sakaki H. Bepotastine besilate, a highly selective histamine H(1) receptor antagonist, suppresses vascular hyperpermeability and eosinophil recruitment in in vitro and in vivo experimental allergic conjunctivitis models. Exp. Eye Res. 2010, 91, 85–91. 10.1016/j.exer.2010.04.006. 20412793

[ref14] De Martino G.; La Regina G.; Di Pasquali A.; Ragno R.; Bergamini A.; Ciaprini C.; Sinistro A.; Maga G.; Crespan E.; Artico M.; Silvestri R. Novel 1-[2-(Diarylmethoxy)ethyl]-2-methyl-5-nitroimidazoles as HIV-1 Non-Nucleoside Reverse Transcriptase Inhibitors. A Structure-Activity Relationship Investigation. J. Med. Chem. 2005, 48, 4378–4388. 10.1021/jm050273a. 15974590

[ref15] Yin L.; Hu Q.; Hartmann R. W. Tetrahydropyrroloquinolinone Type Dual Inhibitors of Aromatase/Aldosterone Synthase as a Novel Strategy for Breast Cancer Patients with Elevated Cardiovascular Risks. J. Med. Chem. 2013, 56 (2), 460–470. 10.1021/jm301408t. 23281812

[ref16] Zeng W. M.; He Y. H.; Guan Z. Direct Reductive Arylation of Imines with Electron-Deficient (Hetero) Arenes via Electrosynthesis to Access Benzylic Amines. Org. Lett. 2022, 24, 7178–7182. 10.1021/acs.orglett.2c02900. 36148976

[ref17] aKamijo S.; Takao G.; Kamijo K.; Hirota M.; Tao K.; Murafuji T. Photo-induced Substitutive Introduction of the Aldoxime Functional Group to Carbon Chains: A Formal Formylation of Non-Acidic C(sp^3^)-H Bonds. Angew. Chem. 2016, 128, 9847–9851. 10.1002/ange.201603810. 27356038

[ref18] Sedelmeier J.; Ley S. V.; Baxendale I. R.; Baumann M. KMnO_4_-Mediated oxidation as a continuous flow process. Org. Lett. 2010, 12, 3618–3621. 10.1021/ol101345z. 20704404

[ref19] Alnafisah A. S.; Alqrairy E.; Tar H.; Alminderej F. M.; Aroua L. M.; Graff B.; Lalevee J. Light-Assisted Synthesis of Silver and Gold Nano particles by New Benzophenone Derivatives. ACS Omega 2023, 8, 3207–3220. 10.1021/acsomega.2c06655. 36713746 PMC9878651

