# Supplementary material for: Modular Synthesis of Benzoylpyridines Exploiting a Reductive Arylation Strategy
Source: Org Lett. 2023 Dec 22;26(14):2847–51. doi: 10.1021/acs.orglett.3c03833 (PMC11020167; doi:10.1021/acs.orglett.3c03833)
Supplement: Supplementary file 1 — ol3c03833_si_001.pdf [file ol3c03833_si_001.pdf]

Modular Synthesis of Benzoylpyridines Exploiting a Reductive Arylation Strategy

Antonella Ilenia Alfano[a], Megan Smyth[b], Scott Wharry[b], Thomas S. Moody[b,c] and Marcus Baumann*[,a]

[a] School of Chemistry, University College Dublin, Science Centre South, Dublin 4, Ireland

[b] Almac Sciences, Technology Department, Craigavon BT63 5QD, United Kingdom

c Arran Chemical Company, Monksland Industrial Estate, Roscommon N37 DN24, Ireland

Table of contents

General materials and methods S2

General experimental protocols S4

Optimization tables S6

Characterization data S14

Unsuccessful substrates S28

References S29

Pictures of the flow equipment S30

Copies of NMR Spectra S32

General materials and methods

Unless otherwise stated, all solvents were purchased from Fisher Scientific and used without further purification. Also, unless otherwise stated, all substrates and reagents were purchased from Fluorochem or Sigma-Aldrich and used as received.

^{1}H NMR spectra were recorded on 400 and 500 MHz instruments and are reported relative to the residual solvent: $CHCl_3$ (δ 7.26 ppm) or DMSO-d_6 (δ 2.50 ppm). ^{13}C{1H} NMR spectra were recorded on the same instruments (100 and 125 MHz) and are reported relative to $CHCl_3$ (δ 77.0 ppm) or DMSO-d_6 (δ 39.52 ppm). ^{19}F NMR were recorded at 376 MHz. Data for ^{1}H NMR are reported as follows: chemical shift (δ/ ppm) (integration, multiplicity, coupling constant (Hz)). Multiplicities are reported as follows: s = singlet, d = doublet, t = triplet, q = quartet, p = pentet, m = multiplet, br s = broad singlet, app = apparent. Data for ^{13}C{1H} NMR are reported in terms of chemical shift (δ/ppm) and multiplicity (C, CH, CH_2, or CH_3). COSY and HSQC experiments were used in the structural assignment.

High-resolution mass spectrometry was performed using the indicated techniques on a micromass LCT orthogonal time-of-flight mass spectrometer with leucine-enkephalin (Tyr-Gly- Phe-Leu) as an internal lock mass.

Spectra were recorded on a Waters GCT Premier Mass Spectrometer calibrated using perfluorotributylamine (PFTBA / FC-43) purchased from Sigma Aldrich. Chromatography was carried out with an Agilent 7890A GC equipped with a Agilent DB-5MS capillary column (15m x 0.250mm). A gradient from 40 to 300 °C was used, with an inlet temperature of 160 °C and a split ratio of 1:10.

For UV/Vis measurements, a Shimadzu UV-1800 UV spectrophotometer was used.

Melting points were recorded with a Stuart SMP10 melting point apparatus and are uncorrected.

Continuous flow experiments were performed on a Vapourtec E-series system equipped with peristaltic pumps and a dynamic BPR achieved through utilisation of a peristaltic pump in a reverse direction (1 bar, Vapourtec). For photochemical experiments the UV-150 module was used in combination with a high-power LED (365 nm) regulated between 33-66 W and cooled to 25-30 °C by passing a stream of compressed air through the reactor unit. Reactor coils were made of PFA tubing (i.d. 1/16 inch) with a volume of 10 mL and 14 mL.

TLC was performed on Merck pre-coated Silica gel 60 F254 aluminium plates with realisation by UV irradiation at 254 nm, $KMnO_4$ and vanillin stain. Flash chromatography was performed using Macherey-Nagel silica gel 60 M, with a particle range of 0.04 - 0.063 mm.

.

General experimental protocols

General procedure A: Synthesis of secondary alcohol library containing para-substituted pyridine.

A solution of aldehyde (0.15 mmol), 4-cyanopyridine (0.30 mmol, 2 equiv.), DIPEA (0.30 mmol, 2 equiv.) in 1.5 mL of MeCN is prepared. Once total solubility is achieved, the solution is degassed with N_2 for 30 seconds. Then, the solution is placed in the reaction tube inlet and the valve is switched to inject the sample, using 0.333 mL/ min as flow rate. Beforehand, all the system is stabilized with the corresponding conditions of intensity, flow rate, and back pressure for 5 minutes. When all the volume is injected, the valve is switched again to the solvent inlet with MeCN. After 30 min of residence time, the solution is collected at the outlet of the reactor in a flask, the solvent is evaporated *in vacuo* and the yield is calculated with qNMR (trichloroethylene as internal standard). The crude is purified through chromatography column (pentane/ethyl acetate) to obtain the clean product.

General procedure B: Synthesis of secondary alcohol library containing ortho-substituted pyridine.

A solution of aldehyde (0.15 mmol), 2-cyanopyridine (0.45 mmol, 3 equiv.), DIPEA (0.30 mmol, 2 equiv.), MeOH (0.9 mmol, 6 equiv.) in 1.5 mL of MeCN is prepared. Once total solubility is achieved, the solution is degassed with N_2 for 30 seconds. Then, the solution is placed in the reaction tube inlet and the valve is switched to inject the sample, using 0.333 mL/ min as flow rate. Beforehand, all the system is stabilized with the corresponding conditions of light intensity, flow rate, and back pressure for 5 minutes. When all the volume is injected, the valve is switched again to the solvent inlet with MeCN. After 30 min of residence time, the solution is collected at the outlet of the reactor in a flask, the solvent is evaporated *in vacuo* and the yield is calculated with qNMR (trichloroethylene as internal standard). The crude is purified through chromatography column (pentane/ethyl acetate) to obtain the clean product.

General procedure C: Synthesis of benzophenone derivatives.

A solution of clean alcohol (0.15 mmol) and $KMnO_4$ (0.45 mmol, 3 equiv.) in 1.5 mL of MeCN/H_2O (1:1) is prepared and placed in the reaction tube inlet. The valve is switched to inject the sample, using the chosen flow rate. When all the volume is injected, the valve is switched again to the solvent inlet with MeCN. The solution is collected at the outlet of the reactor in a flask containing a 10% solution of sodium sulfite. Once all the MeCN/H_2O is evaporated, the residue is redissolved in 1:1 mixture of ethyl acetate and brine. Both phases are separated and the organic layer is washed (2x5

mL) with brine. The organic layer is then dried with sodium sulphate and the solvent evaporated *in vacuo*. The yield is calculated with qNMR (trichloroethylene as internal standard).

General procedure D: Telescoped approach for the synthesis of benzophenone derivatives.

A solution of aldehyde (0.15 mmol), 4-cyanopyridine (0.30 mmol, 2 equiv.), DIPEA (0.30 mmol, 2 equiv.) in MeCN (0.1 M) is prepared. Once total solubility is achieved, the solution is degassed with N_2 for 30 seconds. Then, the solution is placed in the reaction tube inlet and the valve is switched to inject the sample, using 0.333 mL/ min as flow rate. Beforehand, all the system is stabilized with the corresponding conditions of light intensity, flow rate, and back pressure for 5 minutes. When all the volume is injected, the valve is switched again to the solvent inlet with MeCN. After 30 min of residence time, a second solution containing $KMnO_4$ (5 equiv.) in H_2O (0.1 M) is pumped in a second coil (14 mL) with flow rate of 1.52 mL/min. After 7.57 min, the solution is quenched and collected at the outlet of the reactor in a flask containing a 10% solution of sodium sulfite. Once all the MeCN/H_2O is evaporated, the residue is redissolved in 1:1 mixture of ethyl acetate and brine. Both phases are separated and the organic layer is extracted (2x5 mL) with brine. The organic layer is then dried with sodium sulphate and the solvent evaporated *in vacuo*. The yield is calculated with qNMR trichloroethylene as internal standard). The crude is purified through chromatography column (DCM/MeOH) to obtain the clean product.

Optimization tables

Screening conditions for the synthesis of compound **3a**

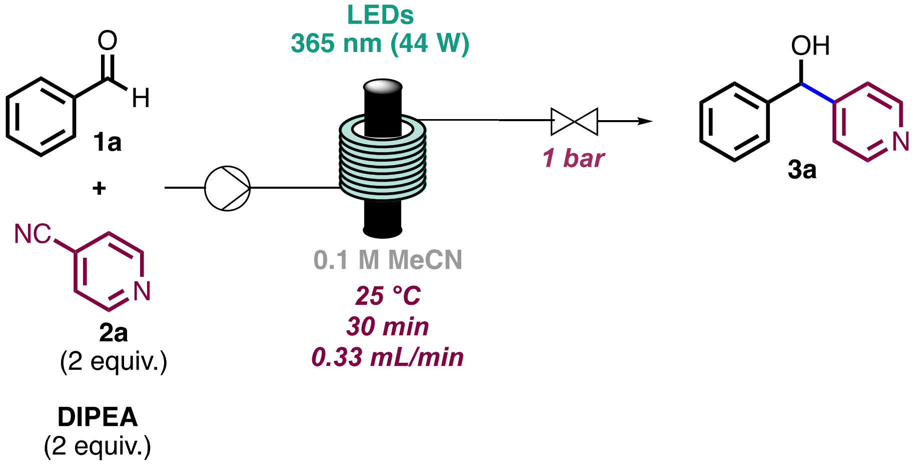

Entry	Deviations from above conditions	[a] Yield % 3a
**1**	**none**	**95**
2	MeCN/H_2O (7:1), 385 nm 70 W	17
3	385 nm, 70 W	34
4	20 min	91
5	dark	-
6	15 min	71
7	15 min, 68 W	66
8	20 min, 68 W	76
9	30 min, 20 W	43
10	420 nm	-
11	45 min	65

Table S1: All reactions were carried out module equipped with a coil reactor (10 mL, PFA). One adjustable back pressure regulator (BPR) was used for all reactions. [a]Yields of **3a** are calculated by ^{1}H NMR using trichloroethylene as internal standard.

Solvent screening for the synthesis of compound **3a**

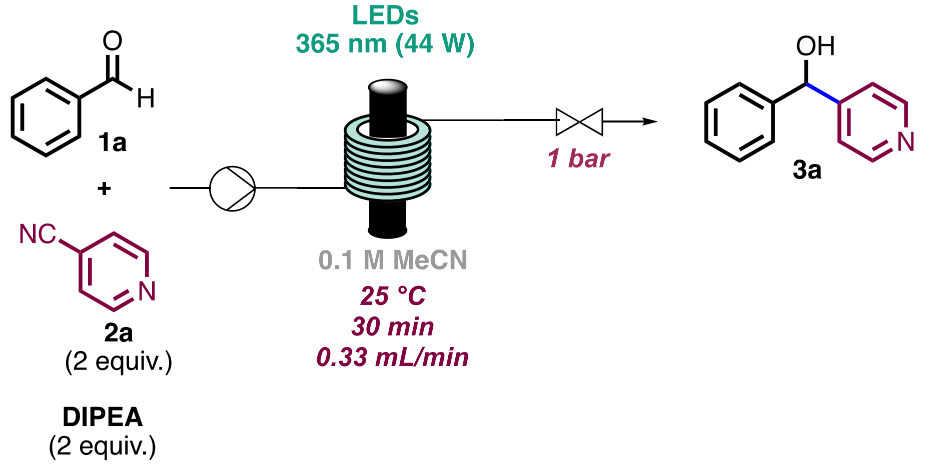

Entry	Deviations from above conditions	[a] Yield % 3a
**1**	**none**	**95**
2	DCM	52
3	MeOH	78
4	MeCN/H_2O (20:1)	90
5	1,4-dioxane	42
6	THF	57

Table S2: All reactions were carried out module equipped with a coil reactor (10 mL, PFA). One adjustable back pressure regulator (BPR) was used for all reactions. [a]Yields of **3a** are calculated by ^{1}H NMR using trichloroethylene as internal standard.

Base screening for the synthesis of compound **3a**

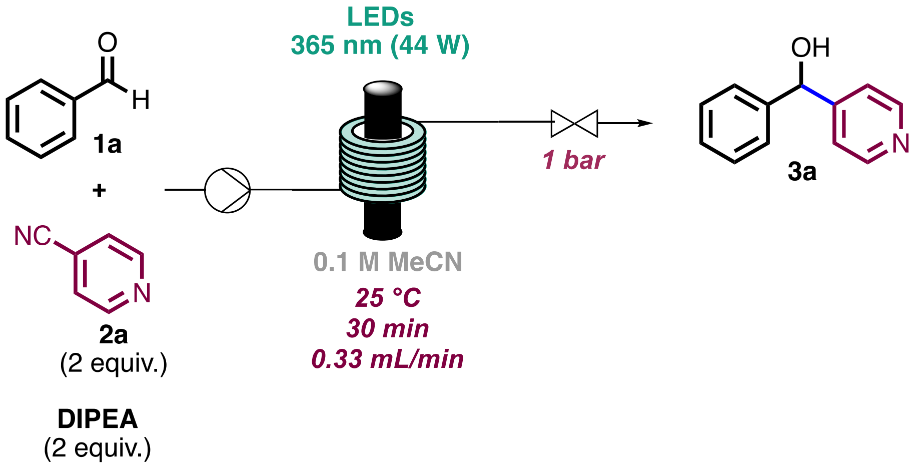

Entry	Deviations from above conditions	[a] Yield % 3a
**1**	**none**	**95**
2	No base	-
3	TEA	60
4	DBU	-
5	1.1 equiv. DIPEA	32

Table S3: All reactions were carried out module equipped with a coil reactor (10 mL, PFA). One adjustable back pressure regulator (BPR) was used for all reactions. [a]Yields of **3a** are calculated by ^{1}H NMR using trichloroethylene as internal standard.

Concentration screening for the synthesis of compound **3a**

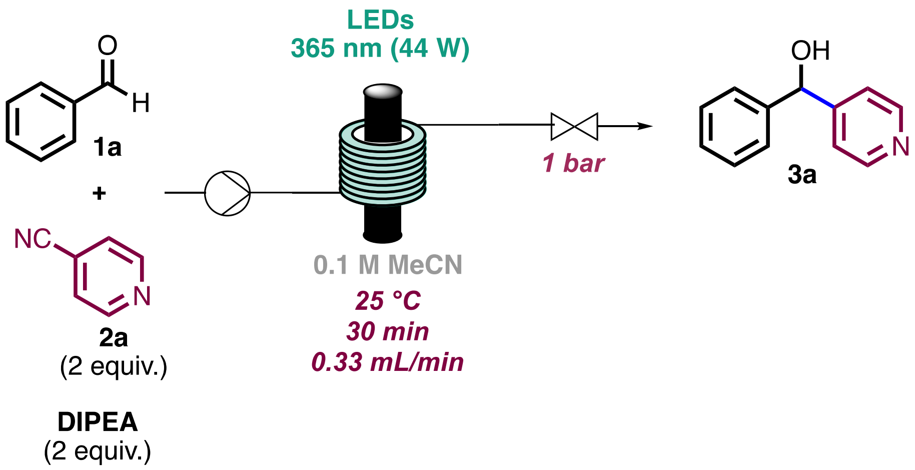

Entry	Deviations from above conditions	[a] Yield % 3a
**1**	**none**	**95**
2	0.2 M	60
3	0.2 M, 50 min	75
4	0.4 M	57
5	0.25 M, 385 nm	41

Table S4: All reactions were carried out module equipped with a coil reactor (10 mL, PFA). One adjustable back pressure regulator (BPR) was used for all reactions [a]Yields of **3a** are calculated by ^{1}H NMR using trichloroethylene as internal standard.

Residence time screening for the synthesis of compound **4a**

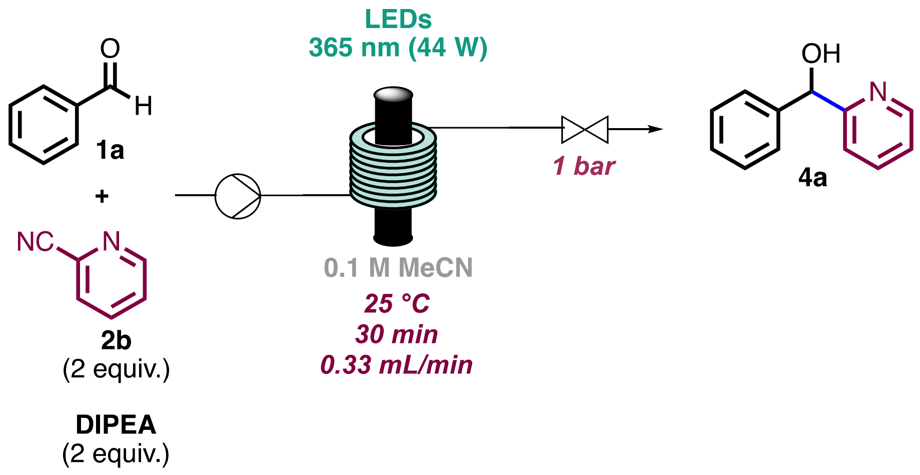

Entry	Deviations from above conditions	[a] Yield % 4a
1	none	55
2	10	37
3	20	40
4	50	41
5	50, 0.2 M	43

Table S5: All reactions were carried out module equipped with a coil reactor (10 mL, PFA). One adjustable back pressure regulator (BPR) was used for all reactions [a]Yields of **4a** are calculated by ^{1}H NMR using trichloroethylene as internal standard.

Solvent, stoichiometry and watt screening for the synthesis of compound **4a**

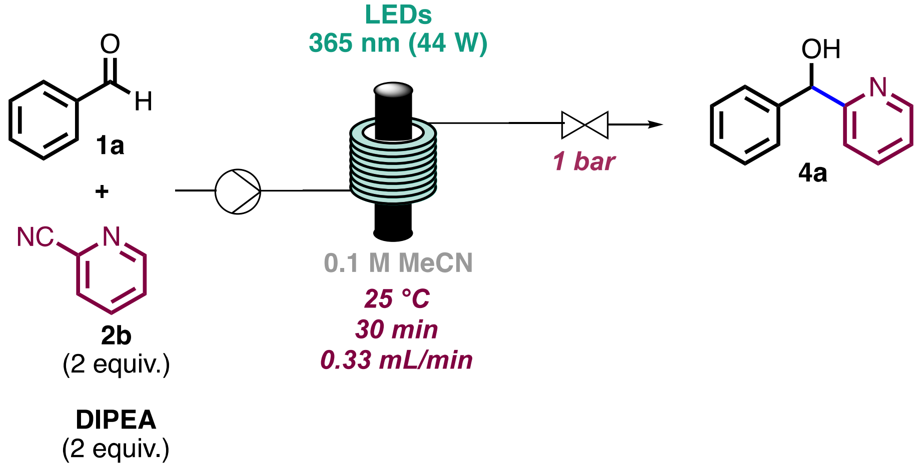

Entry	Deviations from above conditions	[a] Yield % 4a
1	none	55
2	3 equiv. DIPEA, 3 equiv. 2b	47
3	3 equiv. DIPEA, 45 min	48
4	2 equiv. 1a, 1 equiv. 2b	31
5	MeCN/H_2O (3:1), 3 equiv. 2b	53
6	MeOH, 3 equiv. 2b	64
7	MeCN/H_2O (1:1), 3 equiv. 2b	55
8	MeOH, 3 equiv. 2b, 33 W	55
9	MeOH, 3 equiv. 2b, 66 W	68
10	1.5 equiv. MeOH, 3 equiv. 2b, 66 W	54
11	3 equiv. MeOH, 3 equiv. 2b, 66 W	59
**12**	**6 equiv. MeOH, 3 equiv. 2b, 66 W**	**65**
13	20 equiv. MeOH, 3 equiv, 66 W	64
14	MeCN/MeCN (3:1), 3 equiv. 2b, 66 W	56

Table S6: All reactions were carried out module equipped with a coil reactor (10 mL, PFA). One adjustable back pressure regulator (BPR) was used for all reactions. [a]Yields of 4**a** are calculated by ^1H NMR using trichloroethylene as internal standard.

Screening for the synthesis of compound **5a** from **3a**

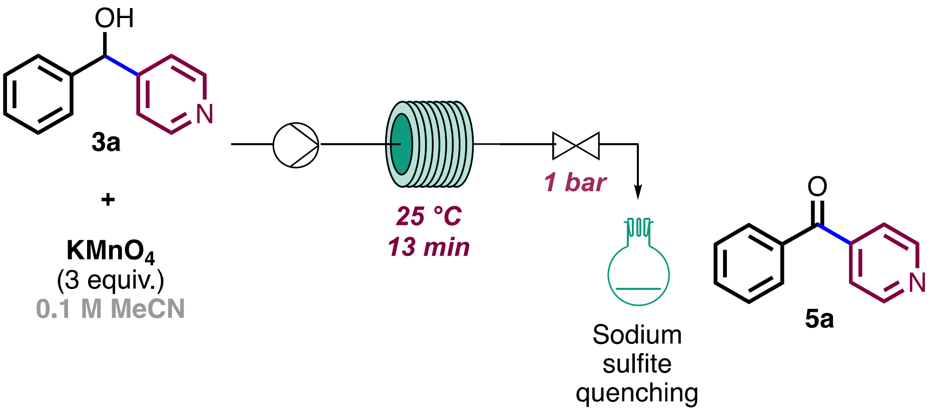

Entry	Deviations from above conditions	[a] Yield % 5a
1	none	70
2	Fed batch, 3 equiv. of $KMnO_4$, MeCN 0.1 M, 30 min,	quantitative
3	2 equiv. TBHP, 0.8 equiv. TFA instead $KMnO_4$, MeCN 0.1 M, 15 min	14
4	H_2O/MeCN 0.1 M	90
**5**	**5 equiv. of $KMnO_4$, H_2O/MeCN (1:1) 0.05 M**	**95**
6	5 min	88
10	10 min	90

Table S7: All reactions were carried out module equipped with a coil reactor (14 mL, PFA). One adjustable back pressure regulator (BPR) was used for all reactions. . [a]Yields of 5**a** are calculated by ^{1}H NMR using trichloroethylene as internal standard.

Optimization for the synthesis of compound **5a** from **1a** and **2a**

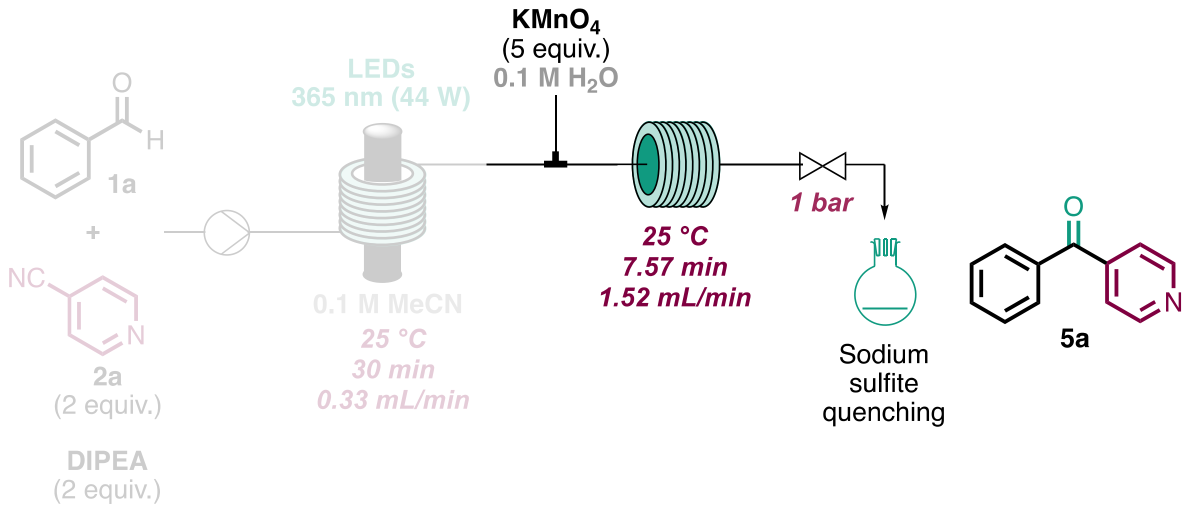

Entry	Deviations from above conditions	[a] Yield % 5a
**1**	**none**	**89**
2	3 equiv. $KMnO_4$, H_2O 0.27 M, 4.54 min	10
3	3 equiv. $KMnO_4$, 4.54 min	37
4	5.17 min	54
5	0.2 M H_2O	80
6	4 equiv., 13 min	62
10[b]	4 equiv., 0.3 M	73

Table S7: All reactions were carried out module equipped with two coil reactors (10 mL and 14 mL, PFA). One adjustable back pressure regulator (BPR) was used for all reactions. . [a]Yields of **5a** are calculated by ^1H NMR using trichloroethylene as internal standard. [b] The reactor is clogged.

Characterization data

3a: Phenyl(pyridin-4-yl)methanol

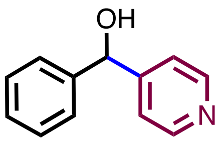

3a

Conditions: Flow rate: 333 μL/min, residence time: 30 min, light intensity: 44 Watt, solvent: MeCN.

Following the general protocol A, using 15 μL of benzaldehyde (0.15 mmol), 31.23 mg of 4-cyanopyridine (0.30 mmol), 52.25 μL of DIPEA, 25.0 mg (89% yield) of **3a** were obtained as white solid, isolated after column chromatography using silica gel and pentane/ethyl acetate 6:4 as eluent. The same reaction was also performed on 1 mmol and 7 mmol scale, furnishing 140.5 mg (76% yield) and 972.4 mg (75% yield) respectively. The spectroscopic data are in line with the previous reported in literature.[1]

M.p 119-120 °C.

^{1}H NMR (400 MHz, $CDCl_3$) δ 8.42 (d, *J* = 6.0 Hz, 2H), 7.36 – 7.28 (m, 7H), 5.77 (s, 1H).

^{13}C NMR (101 MHz, $CDCl_3$) δ 153.2 (C), 149.5 (2 CH), 143.0 (C), 128.9 (2 CH), 128.3 (CH), 126.9 (2 CH), 121.5 (2 CH), 75.0 (CH).

HR- MS (TOF-ES+) calc for $C_{12}H_{12}NO$ 186.0919 $(M+H)^+$, found 186.0919 $(M+H)^+$.

3b: Pyridin-4-yl(p-tolyl)methanol

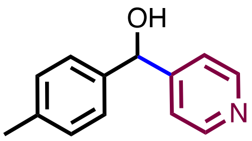

3b

Conditions: Flow rate: 333 μL/min, residence time: 30 min, light intensity: 44 Watt, solvent: MeCN. Following the general protocol A, using 18 μL of p-tolualdehyde (0.15 mmol), 31.23 mg of 4-cyanopyridine (0.30 mmol), 52.25 μL of DIPEA, 27.6 mg (93% yield) of **3b** were obtained as yellowish

solid, isolated after column chromatography using silica gel and pentane/ethyl acetate 6:4 as eluent. The spectroscopic data are in line with the previous reported in literature.[1]

M.p. 113-115°C.

^{1}H NMR (400 MHz, $CDCl_3$) δ 8.45 (d, *J* = 6.1 Hz, 2H), 7.31 – 7.29 (m, 2H), 7.23 – 7.20 (m, 2H), 7.15 (d, *J* = 7.8 Hz, 2H), 5.74 (s, 1H), 2.33 (s, 3H).

^{13}C NMR (101 MHz, $CDCl_3$) δ 153.2 (C), 149.6 (2 CH), 140.1 (CH), 138.2 (CH), 129.6 (2 CH), 126.9 (2 CH), 121.4 (2 CH), 74.9 (CH), 21.3 (CH_3).

HR- MS (TOF-ES+) calc for $C_{13}H_{14}NO$ 200. 1070 $(M+H)^+$, found 200.1070 $(M+H)^+$.

3c: Pyridin-4-yl(o-tolyl)methanol

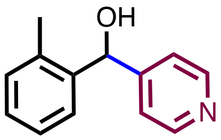

3c

Conditions: Flow rate: 333 µL/min, residence time: 30 min, light intensity: 44 Watt, solvent: MeCN.

Following the general protocol A, using 18 µL of 2-tolualdehyde (0.15 mmol), 31.23 mg of 4-cyanopyridine (0.30 mmol), 52.25 µL of DIPEA, 27.3 mg (93% yield) of **3c** were obtained as white solid, isolated after column chromatography using silica gel and pentane/ethyl acetate 6:4 as eluent. The spectroscopic data are in line with the previous reported in literature.[1]

M.p. 120-122°C.

^{1}H NMR (400 MHz, $CDCl_3$) δ 8.49 (d, *J* = 6.1 Hz, 2H), 7.35 (dd, *J* = 6.8, 2.4 Hz, 1H), 7.33 – 7.29 (m, 2H), 7.28 – 7.24 (m, 2H), 7.24 – 7.19 (m, 1H), 6.03 (s, 1H), 2.35 (s, 3H).

^{13}C NMR (101 MHz, $CDCl_3$) δ 152.8 (C), 149.9 (2 CH), 140.9 (C), 136.2 (C), 131.4 (CH), 128.6 (CH), 127.7 (2 CH), 126.9 (CH), 122.2 (CH), 72.5 (CH), 19.8 (CH_3).

HR- MS (TOF-ES+) calc for $C_{13}H_{14}NO$ 200.1070 $(M+H)^+$, found 200.1071 $(M+H)^+$.

3d: (4-Fluorophenyl)(pyridin-4-yl)methanol

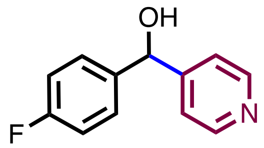

3d

Conditions: Flow rate: 333 µL/min, residence time: 30 min, light intensity: 44 Watt, solvent: MeCN.

Following the general protocol A, using 16 µL of 4-fluorobenzaldehyde (0.15 mmol), 31.23 mg of 4-cyanopyridine (0.30 mmol), 52.25 µL of DIPEA, 19.6 mg (64% yield) of **3d** were obtained as yellowish solid, isolated after column chromatography using silica gel and pentane/ethyl acetate 6:4 as eluent. The spectroscopic data are in line with the previous reported in literature.[1]

M.p. 132-134 °C**.**

^{1}H NMR (400 MHz, CDCl$_3$) δ 8.50 (d, *J* = 6.1 Hz, 2H), 7.34 – 7.28 (m, 4H), 7.06 – 7.01 (m, 2H), 5.79 (s, 1H).

^{13}C NMR (101 MHz, CDCl$_3$) δ 162.7 (d, *J* = 247.1 Hz, CF), 152.5 (C), 149.9 (2 CH), 138.7 (d, *J* = 2.9 Hz, C), 128.72 (d, *J* = 8.2 Hz, 2 CH), 121.31 (2 CH), 115.88 (d, *J* = 21.7 Hz, 2 CH), 74.44 (CH).

^{19}F NMR (470 MHz, CDCl$_3$) δ -113.53 (tt, *J* = 8.6, 5.4 Hz).

HR- MS (TOF-ES+) calc for $C_{12}H_{11}FNO$ 204.0746 $(M+H)^+$, found 204.0736 $(M+H)^+$.

3e: (4-Bromophenyl)(pyridin-4-yl)methanol

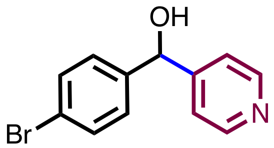

3e

Conditions: Flow rate: 333 µL/min, residence time: 30 min, light intensity: 44 Watt, solvent: MeCN.

Following the general protocol A, using 27.7 mg of 4-bromobenzaldehyde (0.15 mmol), 31.23 mg of 4-cyanopyridine (0.30 mmol), 52.25 µL of DIPEA, 25 mg (62% yield) of **3e** were obtained as yellowish solid, isolated after column chromatography using silica gel and pentane/ethyl acetate 6:4 as eluent.

M.p. 130-133 °C.

^{1}H NMR (400 MHz, CDCl$_3$) δ 8.47 (d, *J* = 6.1 Hz, 2H), 7.47 (d, *J* = 8.5 Hz, 2H), 7.29 (d, *J* = 6.2 Hz, 2H), 7.22 (d, *J* = 8.4 Hz, 2H), 5.75 (s, 1H).

^{13}C NMR (101 MHz, CDCl$_3$) δ 152.5 (C), 149.6 (2 CH), 141.8 (C), 132.1 (2 CH), 129.0 (2 CH), 122.3 (2 CH), 121.4 (C), 74.4 (CH).

HR- MS (TOF-ES+) calc for $C_{12}H_{11}BrNO$ 264.0019 $(M+H)^+$, found 264.0020 and 266.0000 $(M+H)^+$.

3f: (2-Chlorophenyl)(pyridin-4-yl)methanol

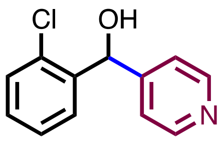

3f

Conditions: Flow rate: 333 µL/min, residence time: 30 min, light intensity: 44 Watt, solvent: MeCN.

Following the general protocol A, using 21.0 mg of 2-chlorobenzaldehyde (0.15 mmol), 31.23 mg of 4-cyanopyridine (0.30 mmol), 52.25 µL of DIPEA, 23 mg (70% yield) of **3f** were obtained as yellowish solid, isolated after column chromatography using silica gel and pentane/ethyl acetate 6:4 as eluent.

M.p. 125-127 °C.

^{1}H NMR (400 MHz, CDCl$_3$) δ 8.48 (d, *J* = 6.2 Hz, 2H), 7.48 (dd, *J* = 7.5, 2.0 Hz, 1H), 7.37 (dd, *J* = 7.7, 1.5 Hz, 1H), 7.34 (d, *J* = 7.0 Hz, 2H), 7.31 – 7.22 (m, 2H), 6.25 (s, 1H).

^{13}C NMR (101 MHz, CDCl$_3$) δ 151.7 (C), 149.7 (2 CH), 140.2 (C), 132.7 (C), 129.8 (CH), 129.5 (CH), 128.6 (CH), 127.6 (CH), 121.7 (2 CH), 71.2 (CH).

HR- MS (TOF-ES+) calc for $C_{12}H_{11}ClNO$ 220.0524 $(M+H)^+$, found 220.0524 $(M+H)^+$.

3g: (3,4-Dichlorophenyl)(pyridin-4-yl)methanol

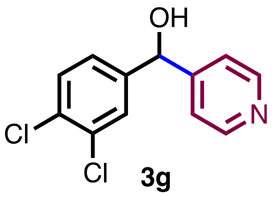

3g

Conditions: Flow rate: 333 µL/min, residence time: 30 min, light intensity: 44 Watt, solvent: MeCN.

Following the general protocol A, using 26.0 mg of 3,4-dichlorobenzaldehyde (0.15 mmol), 31.23 mg of 4-cyanopyridine (0.30 mmol), 52.25 µL of DIPEA, 25.3 mg (66% yield) of **3g** were obtained as yellowish solid, isolated after column chromatography using silica gel and pentane/ethyl acetate 6:4 as eluent.

M.p. 138-140 °C.

^{1}H NMR (400 MHz,$CDCl_3$) δ 8.49 (d, *J* = 6.2 Hz, 2H), 7.47 (d, *J* = 2.1 Hz, 1H), 7.41 (d, *J* = 8.3 Hz, 1H), 7.29 (ddd, *J* = 4.5, 1.7, 0.8 Hz, 2H), 7.17 (ddd, *J* = 8.3, 2.1, 0.6 Hz, 1H), 5.74 (s, 1H).

^{13}C NMR (101 MHz, $CDCl_3$) δ 152.0 (C), 149.9 (2 CH), 142.9 (C), 133.1 (C), 132.4 (C), 130.9 (CH), 128.8 (2 CH), 126.1 (CH), 121.4 (CH), 73.9 (CH).

HR- MS (TOF-ES+) calc for $C_{12}H_{10}Cl_2NO$ 254.0134 $(M+H)^+$, found 254.0134 $(M+H)^+$.

3h: (4-Chloro-3-(trifluoromethyl)phenyl)(pyridin-4-yl)methanol

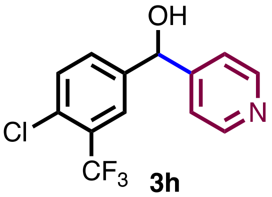

3h

Conditions: Flow rate: 333 µL/min, residence time: 30 min, light intensity: 44 Watt, solvent: MeCN.

Following the general protocol A, using 31.28 mg of 4-chloro-3-(trifluoromethyl)benzaldehyde (0.15 mmol), 31.23 mg of 4-cyanopyridine (0.30 mmol), 52.25 µL of DIPEA, 34.5 mg (80% yield) of **3h** were obtained as yellowish solid, isolated after column chromatography using silica gel and pentane/ethyl acetate 6:4 as eluent.

M.p. 138-140 °C**.**

^{1}H NMR (400 MHz, CDCl$_3$) δ 8.57 (s, 2H), 7.72 (d, *J* = 2.7 Hz, 1H), 7.49 (d, *J* = 8.4 Hz, 1H), 7.45 (dd, *J* = 8.3, 2.1 Hz, 1H), 7.30 (d, *J* = 6.0 Hz, 2H), 5.84 (s, 1H).

^{13}C NMR (126 MHz, CDCl$_3$) δ 151.3 (C), 150.4 (2 CH), 141.6 (C), 132.1 (2 CH), 131.1 (2 CH), 129.34 (C), 125.9 (q, *J* = 5.2 Hz, C), 123.85 (CH), 121.24 (q, *J* = 261 Hz, CF$_3$), 74.08 (CH).

^{19}F NMR (470 MHz, CDCl$_3$) δ -62.55.

HR- MS (TOF-ES+) calc for $C_{13}H_{10}ClF_3NO$ 288.0325, found 288.0328.

3i: (3-Chloro-4-fluorophenyl)(pyridin-4-yl)methanol

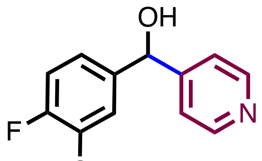

3i

Conditions: Flow rate: 333 µL/min, residence time: 30 min, light intensity: 44 Watt, solvent: MeCN.

Following the general protocol A, using 24 mg 3-chloro-4-fluorobenzaldehyde (0.15 mmol), 31.23 mg of 4-cyanopyridine (0.30 mmol), 52.25 µL of DIPEA, 25.5 mg (71% yield) of **3i** were obtained as yellowish solid, isolated after column chromatography using silica gel and pentane/ethyl acetate 6:4 as eluent.

M.p. 143-145 °C**.**

^{1}H NMR (400 MHz, CDCl$_3$) δ 8.56 (d, *J* = 6.2 Hz, 2H), 7.42 (dd, *J* = 6.9, 2.6 Hz, 1H), 7.29 (ddd, *J* = 4.5, 1.7, 0.7 Hz, 2H), 7.21 (dddd, *J* = 8.5, 4.6, 2.2, 0.6 Hz, 1H), 7.12 (t, *J* = 8.6 Hz, 1H), 5.76 (s, 1H).

^{13}C NMR (151 MHz, CDCl$_3$) δ 157.9 (d, *J* = 250.3 Hz, CF), 152.1 (C), 150.0 (2 CH), 139.8 (3 CH), 129.1 (C) 126.6 (d, *J* = 7.5 MHz, CH), 121.3 (C), 117.03 (d, *J* = 26.6 Hz, CH) 73.94 CH).

^{19}F NMR (470 MHz, CDCl$_3$) δ -115.69 (ddd, *J* = 9.0, 6.9, 4.5 Hz).

HR- MS (TOF-ES+) calc for $C_{12}H_{10}ClFNO$ 238.0429 $(M+H)^+$, found 238.0431 $(M+H)^+$.

3j: (3,5-Dimethoxyphenyl)(pyridin-4-yl)methanol

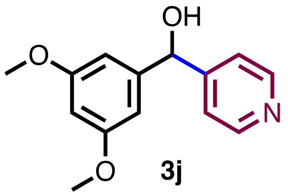

3j

Conditions: Flow rate: 333 µL/min, residence time: 30 min, light intensity: 44 Watt, solvent: MeCN.

Following the general protocol A, using 24.9 mg of 3,5-dimethoxybenzaldehyde (0.15 mmol), 31.23 mg of 4-cyanopyridine (0.30 mmol), 52.25 µL of DIPEA, 15.7 mg (42% yield) of **3j** were obtained as yellowish solid, isolated after column chromatography using silica gel and pentane/ethyl acetate 4:6 as eluent. The spectroscopic data are in line with the previous reported in literature.[2]

M.p. 139-140 °C.

^{1}H NMR (500 MHz, DMSO) δ 8.47 (d, J = 6.0 Hz, 2H), 7.39 – 7.37 (m, 2H), 6.56 (d, *J* = 2.3 Hz, 2H), 6.35 (t, *J* = 2.3 Hz, 1H), 6.11 (d, *J* = 4.2 Hz, 1H), 5.62 (d, *J* = 4.2 Hz, 1H), 3.70 (s, 6H).

^{13}C NMR (126 MHz, DMSO) δ 160.7 (2 C), 154.2 (C), 149.7 (2 CH), 147.1 (C), 121.5 (2 CH), 104.6 (2 CH), 99.1 (CH), 73.3 (CH), 55.5 (2 CH_3).

HR- MS (TOF-ES+) calc for $C_{14}H_{16}NO_3$ 246.1125 $(M+H)^+$, found 246.1125 $(M+H)^+$.

<u>**3k**: (4-Methoxyphenyl)(pyridin-4-yl)methanol</u>

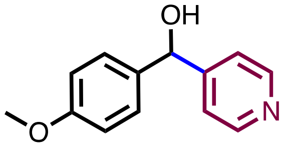

3k

Conditions: Flow rate: 333 µL/min, residence time: 30 min, light intensity: 44 Watt, solvent: MeCN.

Following the general protocol A, using 19 µL of 4-methoxybenzaldehyde (0.15 mmol), 31.23 mg of 4-cyanopyridine (0.30 mmol), 52.25 µL of DIPEA, 12.5 mg (35% yield) of **3k** were obtained as white solid, isolated after column chromatography using silica gel and pentane/ethyl acetate 4:6 as eluent. The spectroscopic data are in line with the previous reported in literature.[1]

M.p. 135-137 °C.

^{1}H NMR (400 MHz, $CDCl_3$) δ 8.51 (d, *J* = 6.1 Hz, 2H), 7.31 (d, *J* = 7.0 Hz, 2H), 7.25 (d, *J* = 8.7 Hz, 2H), 6.88 (d, *J* = 8.9 Hz, 2H), 5.76 (s, 1H), 3.80 (s, 3H).

^{13}C NMR (101 MHz, CDCl$_3$) δ 159.7 (C), 152.8 (C), 149.8 (2 CH), 135.0 (2 CH), 128.3 (2 CH), 121.2 (C), 114.3 (2 CH), 74.7 (CH), 55.4 (CH$_3$).

HR- MS (TOF-ES+) calc for $C_{13}H_{14}NO_2$ 216.1019 $(M+H)^+$, found 216.1020 $(M+H)^+$.

3l: 4-(Hydroxy(pyridin-4-yl)methyl)benzonitrile

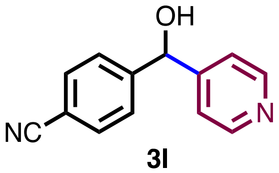

3l

Conditions: Flow rate: 333 µL/min, residence time: 30 min, light intensity: 44 Watt, solvent: MeCN.

Following the general protocol A, using 20 mg of 4-formylbenzonitrile (0.15 mmol), 31.23 mg of 4-cyanopyridine (0.30 mmol), 52.25 µL of DIPEA, 16.5 mg (52% yield) of **3l** were obtained as yellowish solid, isolated after column chromatography using silica gel and pentane/ethyl acetate 4:6 as eluent.

M.p. 140-142 °C.

^{1}H NMR (400 MHz, CDCl$_3$) δ 8.55 (d, J = 6.1 Hz, 2H), 7.65 (d, J = 8.7 Hz, 2H), 7.50 (d, J = 8.7 Hz, 2H), 7.29 (d, J = 5.4 Hz, 2H), 5.85 (s, 1H).

^{13}C NMR (101 MHz, CDCl$_3$) δ 151.3 (C), 150.2 (2 CH), 147.5 (C), 132.7 (2 CH), 127.3 (2 CH), 121.2 (2 CH), 118.5 (C), 112.2 (C), 74.4 (CH).

HR- MS (TOF-ES+) calc for $C_{13}H_{11}N_2O$ 211.0866 $(M+H)^+$, found 211.0868 $(M+H)^+$.

3m: (3-Methoxyphenyl)(pyridin-4-yl)methanol

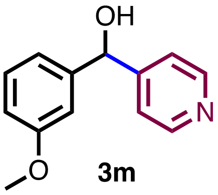

3m

Conditions: Flow rate: 333 µL/min, residence time: 30 min, light intensity: 44 Watt, solvent: MeCN.

Following the general protocol A, using 19 µL of 3-methoxybenzaldehyde (0.15 mmol), 31.23 mg of 4-cyanopyridine (0.30 mmol), 52.25 µL of DIPEA, 31.6 mg (98% yield) of **3m** were obtained as yellowish solid, isolated after column chromatography using silica gel and pentane/ethyl acetate 4:6 as eluent. The same reaction was also performed on 1 mmol furnishing 85% yield. The spectroscopic data are in line with the previous reported in literature.[1]

M.p. 116-118 °C.

^{1}H NMR (500 MHz, $CDCl_3$) δ 8.49 (d, *J* = 6.2 Hz, 2H), 7.32 (d, *J* = 7.0 Hz, 2H), 7.29 – 7.25 (m, 1H), 6.92 (d, *J* = 7.6 Hz, 1H), 6.91 – 6.89 (m, 1H), 6.84 (ddd, *J* = 8.3, 2.7, 1.1 Hz, 1H), 5.76 (s, 1H), 3.78 (s, 3H).

^{13}C NMR (126 MHz, $CDCl_3$) δ 160.3 (C), 152.8 (C), 150.0 (2 CH), 144.7 (C), 130.3 (CH), 121.6 (2 CH), 119.4 (CH), 114.0 (CH), 112.8 (CH), 75.3 (CH), 55.7 (CH_3).

HR- MS (TOF-ES+) calc for $C_{13}H_{14}NO_2$ 216.1019 $(M+H)^+$, found 216.1022 $(M+H)^+$.

3n: Pyridin-4-yl(3-(trifluoromethoxy)phenyl)methanol

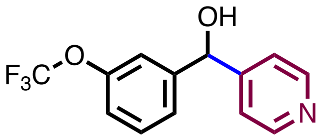

3n

Conditions: Flow rate: 333 µL/min, residence time: 30 min, light intensity: 44 Watt, solvent: MeCN.

Following the general protocol A, using 21.44 µL of 3-trifluoromethoxybenzaldehyde (0.15 mmol), 31.23 mg of 4-cyanopyridine (0.30 mmol), 52.25 µL of DIPEA, 28 mg (70% yield) of **3n** were obtained as yellowish solid, isolated after column chromatography using silica gel and pentane/ethyl acetate 4:6 as eluent.

M.p. 120-122 °C.

^{1}H NMR (400 MHz, $CDCl_3$) δ 8.48 (d, *J* = 6.1 Hz, 2H), 7.37 (t, *J* = 8.2 Hz, 1H), 7.30 (d, *J* = 6.1 Hz, 2H), 7.26 (d, *J* = 7.3 Hz, 2H), 7.17 – 7.13 (m, 1H), 5.80 (s, 1H).

^{13}C NMR (101 MHz, $CDCl_3$) δ 152.1 (C), 149.8 (2 CH), 149.6 (C), 145.1 (C), 130.3 (CH), 125.1 (2 CH), 121.3 (CH), 120.5 (q, *J* = 258 Hz, CF_3), 119.3 (2 CH), 74.3 (CH).

^{19}F NMR (376 MHz, $CDCl_3$) δ -57.7.

HR- MS (TOF-ES+) calc for $C_{13}H_{11}F_3NO_2$ 270.0736 $(M+H)^+$, found 270.0738 $(M+H)^+$.

3o: Naphthalen-2-yl(pyridin-4-yl)methanol

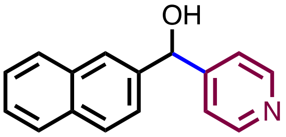

3o

Conditions: Flow rate: 333 μL/min, residence time: 30 min, light intensity: 44 Watt, solvent: MeCN.

Following the general protocol A, using 23.4 mg of naphtaldehyde (0.15 mmol), 31.23 mg of 4-cyanopyridine (0.30 mmol), 52.25 μL of DIPEA, 34.6 mg (98% yield) of **3o** were obtained as white solid, isolated after column chromatography using silica gel and pentane/ethyl acetate 7:3 as eluent. The spectroscopic data are in line with the previous reported in literature.[3]

M.p. 125-127 °C.

^{1}H NMR (400 MHz, CDCl$_3$) δ 8.51 (d, *J* = 6.1 Hz, 2H), 7.85 – 7.80 (m, 4H), 7.52 – 7.47 (m, 2H), 7.39 (dd, *J* = 8.5, 1.8 Hz, 1H), 7.36 (d, *J* = 6.8 Hz, 2H), 5.96 (s, 1H).

^{13}C NMR (101 MHz, CDCl$_3$) δ 152.6 (C), 149.7 (2 CH), 140.1 (C), 133.3 (C), 133.2 (C), 128.9 (2 CH), 128.1 (CH), 127.9 (CH), 126.6 (CH), 126.5 (CH), 125.8 (CH), 124.6 (CH), 121.5 (CH), 75.2 (CH).

HR- MS (TOF-ES+) calc for $C_{16}H_{14}NO$ 236.1070 $(M+H)^+$, found 236.1070 $(M+H)^+$.

3p: (3-Methylthiophen-2-yl)(pyridin-4-yl)methanol

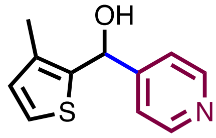

3p

Conditions: Flow rate: 333 μL/min, residence time: 30 min, light intensity: 44 Watt, solvent: MeCN.

Following the general protocol A, using 16.17 μL of 3-methyl-2-thiophenecarboxaldehyde (0.15 mmol), 31.23 mg of 4-cyanopyridine (0.30 mmol), 52.25 μL of DIPEA, 23.4 mg (78% yield) of **3p** were

obtained as white solid, isolated after column chromatography using silica gel and pentane/ethyl acetate 6:4 as eluent. The spectroscopic data are in line with the previous reported in literature.[2]

M.p. 114-116 °C.

^{1}H NMR (400 MHz, $CDCl_3$) δ 8.54 (d, *J* = 6.2 Hz, 2H), 7.35 (d, *J* = 6.1 Hz, 2H), 7.19 (d, *J* = 5.1 Hz, 1H), 6.82 (d, *J* = 5.0 Hz, 1H), 6.08 (s, 1H), 2.25 (s, 3H).

^{13}C NMR (101 MHz, $CDCl_3$) δ 151.9 (C), 150.0 (2 CH), 139.5 (C), 135.1 (C), 130.5 (2 CH), 124.7 (CH), 121.2 (CH), 69.1 (CH), 14.2 (CH_3).

HR- MS (TOF-ES+) calc for $C_{11}H_{12}NOS$ 206.0634 $(M+H)^+$, found 206.0636 $(M+H)^+$.

<u>**3o:** Pyridin-3-yl(pyridin-4-yl)methanol</u>

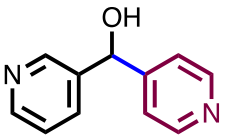

3q

Conditions: Flow rate: 333 μL/min, residence time: 30 min, light intensity: 44 Watt, solvent: MeCN.

Following the general protocol A, using 14 μL of 3-pyridinecarboxaldehyde (0.15 mmol), 31.23 mg of 4-cyanopyridine (0.30 mmol), 52.25 μL of DIPEA, 16.8 mg (59% yield) of **3q** were obtained as white solid, isolated after column chromatography using silica gel and pentane/ethyl acetate 2:8 as eluent. The spectroscopic data are in line with the previous reported in literature.[4]

M.p. 108-110 °C.

^{1}H NMR (400 MHz, $CDCl_3$) δ 8.48 (m, 4H), 7.66 (d, *J* = 7.8 Hz, 1H), 7.35 – 7.26 (m, 3H), 5.81 (s, 1H).

^{13}C NMR (101 MHz, $CDCl_3$) δ 152.3 (2 CH), 149.8 (2 CH), 149.2 (CH), 148.2 (C), 134.7 (C), 124.0 (2 CH), 121.5 (CH), 72.6 (CH).

HR- MS (TOF-ES+) calc for $C_{11}H_{11}N_2O$ 187.0866 $(M+H)^+$, found 187.0866 $(M+H)^+$.

<u>**4a:** Phenyl(pyridin-2-yl)methanol</u>

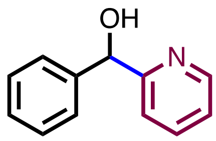

4a

Conditions: Flow rate: 333 µL/min, residence time: 30 min, light intensity: 44 Watt, solvent: MeCN.

Following the general protocol B, using 15 µL of benzaldehyde (0.15 mmol), 47 mg of 2-cyanopyridine (0.45 mmol), 52.25 µL of DIPEA, 40 µL of MeOH (0.9 mmol), 18 mg (65% yield) of **4a** were obtained as white solid, isolated after column chromatography using silica gel and pentane/ethyl acetate 6:4 as eluent. The spectroscopic data are in line with the previous reported in literature.[2,5]

M.p. 75-77 °C.

^{1}H NMR (400 MHz, $CDCl_3$) δ 8.57 (d, *J* = 4.9 Hz, 1H), 7.62 (td, *J* = 7.7, 1.7 Hz, 1H), 7.41 – 7.34 (m, 3H), 7.33 (d, *J* = 7.8 Hz, 1H), 7.28 (d, *J* = 7.2 Hz, 1H), 7.22 – 7.17 (m, 1H), 7.15 (d, *J* = 7.9 Hz, 1H), 5.75 (s, 1H), 5.27 (s, 1H).

^{13}C NMR (101 MHz, $CDCl_3$) δ 161.0 (C), 148.0 (C), 143.4 (CH), 137.0 (CH), 128.7 (2 CH), 128.0 (CH), 127.2 (2 CH), 122.6 (CH), 121.2 (CH), 75.1 (CH).

HR- MS (TOF-ES+) calc for $C_{12}H_{12}NO$ $(M+H)^+$ 186.0841, found 186.0841 $(M+H)^+$.

4b: Pyridin-2-yl(p-tolyl)methanol

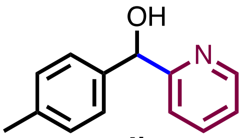

4b

Conditions: Flow rate: 333 µL/min, residence time: 30 min, light intensity: 44 Watt, solvent: MeCN.

Following the general protocol B, using 18 µL of 4-methylbenzaldehyde (0.15 mmol), 47 mg of 2-cyanopyridine (0.45 mmol), 52.25 µL of DIPEA, 40 µL of MeOH (0.9 mmol), 18 mg (60% yield) of **4b** were obtained as white solid, isolated after column chromatography using silica gel and pentane/ethyl acetate 6:4 as eluent. The spectroscopic data are in line with the previous reported in literature.[5]

M.p. 60-62 °C.

^{1}H NMR (400 MHz, CDCl$_3$) δ 8.56 (d, *J* = 3.8 Hz, 1H), 7.61 (td, *J* = 7.7, 1.7 Hz, 1H), 7.26 (d, *J* = 8.2 Hz, 2H), 7.21 – 7.17 (m, 1H), 7.14 (d, *J* = 7.8 Hz, 3H), 5.72 (s, 1H), 5.19 (s, 1H), 2.33 (s, 3H).

^{13}C NMR (101 MHz, CDCl$_3$) δ 161.2 (C), 147.9 (CH), 140.5 (C), 137.7 (C), 136.9 (CH), 129.4 (2 CH), 127.2 (2 CH), 122.5 (CH), 121.5 (CH), 74.9 (CH), 21.3 (CH$_3$).

HR- MS (TOF-ES+) calc for $C_{13}H_{14}NO$ 200.1075 $(M+H)^+$, found 200.1070 $(M+H)^+$.

4c: Pyridin-2-yl(*o*-tolyl)methanol

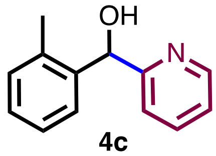

4c

Conditions: Flow rate: 333 µL/min, residence time: 30 min, light intensity: 44 Watt, solvent: MeCN.

Following the general protocol B, using 18 µL of 2-methylbenzaldehyde (0.15 mmol), 47 mg of 2-cyanopyridine (0.45 mmol), 52.25 µL of DIPEA, 40 µL of MeOH (0.9 mmol), 13.6 mg (45% yield) of **4c** were obtained as yellowish solid, isolated after column chromatography using silica gel and pentane/ethyl acetate 6:4 as eluent. The spectroscopic data are in line with the previous reported in literature.[6]

M.p. 58-60 °C.

^{1}H NMR (400 MHz, CDCl$_3$) δ 8.59 (d, *J* = 2.9 Hz, 1H), 7.61 (td, *J* = 7.6, 1.7 Hz, 1H), 7.25 – 7.14 (m, 5H), 7.02 (d, *J* = 7.0 Hz, 1H), 5.97 (s, 1H), 2.35 (s, 3H).

^{13}C NMR (101 MHz, CDCl$_3$) δ 161.3 (C), 148.3 (CH), 141.1 (CH), 137.3 (C), 136.8 (C), 131.3 (CH), 128.6 (CH), 128.3 (CH), 126.6 (CH), 122.8 (CH), 121.7 (CH), 73.3 (CH), 19.93 (CH$_3$).

HR- MS (TOF-ES+) calc for $C_{13}H_{14}NO$ 200.1075 $(M+H)^+$, found 200.1070 $(M+H)^+$.

4d: (4-Fluorophenyl)(pyridin-2-yl)methanol

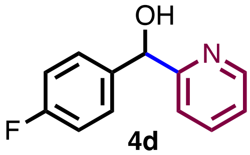

4d

Conditions: Flow rate: 333 µL/min, residence time: 30 min, light intensity: 44 Watt, solvent: MeCN.

Following the general protocol B, using 16 µL of 4-fluorobenzaldehyde (0.15 mmol), 47 mg of 2-cyanopyridine (0.45 mmol), 52.25 µL of DIPEA, 40 µL of MeOH (0.9 mmol), 19.2 mg (63% yield) of **4d** were obtained as yellowish solid, isolated after column chromatography using silica gel and pentane/ethyl acetate 6:4 as eluent. The spectroscopic data are in line with the previous reported in literature.[5]

M.p. 75-80 °C.

^{1}H NMR (500 MHz, CDCl$_3$) δ 8.57 (d, *J* = 5.0 Hz, 1H), 7.63 (td, *J* = 7.6, 1.8 Hz, 1H), 7.37 – 7.32 (m, 2H), 7.21 (dd, *J* = 6.1, 4.6 Hz, 1H), 7.11 (d, *J* = 8.7 Hz, 1H), 7.05 – 6.99 (m, 2H), 5.73 (s, 1H), 5.29 (d, *J* = 6.3 Hz, 1H).

^{13}C NMR (101 MHz, CDCl$_3$) δ 162.9 (d, *J* = 246.1 Hz, CF), 161.1 (C), 148.3 (CH), 139.5 (d, *J* = 3.4 Hz, C), 137.4 (CH), 129.2 (d, *J* = 8.2 Hz, 2 CH), 123.0 (CH), 121.7 (CH), 115.9 (d, *J* = 21.7 Hz, 2 CH), 74.7 (CH).

^{19}F NMR (376 MHz, CDCl$_3$) δ -114.63.

HR- MS (TOF-ES+) calc for $C_{12}H_{11}FNO$ 204.0746 $(M+H)^+$, found 204.0746 $(M+H)^+$.

5a: Phenyl(pyridin-4-yl)methanone

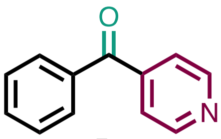

5a

Conditions: First step, flow rate: 333 µL/min, residence time: 30 min, light intensity: 44 Watt, solvent: MeCN. Second step, flow rate: 1.52 mL/min, residence time: 7.57 min, solvent: MeCN/H_2O.

Following the general protocol D, using 15 µL of benzaldehyde (0.15 mmol), 31.23 mg of 4-cyanopyridine (0.30 mmol), 52.25 µL of DIPEA in the first step and 102 mg of $KMnO_4$ (0.75 mmol) in the second step, 22.6 mg (83% yield) of **5a** were obtained as white solid, isolated after column

chromatography using silica gel and DCM/MeOH 96:4 as eluent. The spectroscopic data are in line with the previous reported in literature.[7]

M.p. 72.5-73.0 °C.

^{1}H NMR (400 MHz, $CDCl_3$) δ 8.81 (d, *J* = 6.1 Hz, 2H), 7.82 (d, *J* = 8.4 Hz, 2H), 7.68 – 7.63 (m, 1H), 7.58 (d, *J* = 6.0 Hz, 2H), 7.52 (t, *J* = 8.0 Hz, 2H).

^{13}C NMR (101 MHz,$CDCl_3$) δ 195.3 (C), 150.5 (2 CH), 144.5 (C), 136.1 (C), 133.7 (CH), 130.3 (2 CH), 128.8 (2 CH), 123.0 (2 CH).

GC- MS: calc for $C_{12}H_9NO$ 183.0684, found 183.0684, retention time 14.70 min.

5b: Pyridin-4-yl(*o*-tolyl)methanone

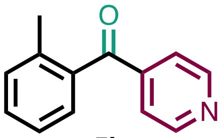

5b

Conditions: First step, flow rate: 333 µL/min, residence time: 30 min, light intensity: 44 Watt, solvent: MeCN. Second step, flow rate: 1.52 mL/min, residence time: 7.57 min, solvent: MeCN/H_2O.

Following the general protocol D, using 18 µL of 2-tolualdehyde (0.15 mmol), 31.23 mg of 4-cyanopyridine (0.30 mmol), 52.25 µL of DIPEA in the first step and 102 mg of $KMnO_4$ (0.75 mmol) in the second step, 25.0 mg (85% yield) of **5b** were obtained as white solid, isolated after column chromatography using silica gel and DCM/MeOH 96:4 as eluent. The spectroscopic data are in line with the previous reported in literature.[8]

M.p. 110-112 ° C

^{1}H NMR (500 MHz, $CDCl_3$) δ 8.80 (d, J = 5.2 Hz, 2H), 7.60 – 7.57 (m, 2H), 7.45 (td, J = 7.5, 1.5 Hz, 1H), 7.35 – 7.31 (m, 2H), 7.29 – 7.26 (m, 1H), 2.40 (s, 3H).

^{13C} NMR (126 MHz, $CDCl_3$) δ 197.4, 150.8, 144.4, 138.1, 136.7, 131.7, 131.6, 129.6, 125.5, 123.0, 20.5.

GC- MS: calc for $C_{13}H_{11}NO$ 197.0841, found 197.0841, retention time 14.85 min.

5c: (3-Methoxyphenyl)(pyridin-4-yl)methanone

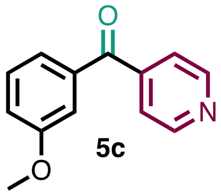

5c

Conditions: First step, flow rate: 333 µL/min, residence time: 30 min, light intensity: 44 Watt, solvent: MeCN. Second step, flow rate: 1.52 mL/min, residence time: 7.57 min, solvent: MeCN/H_2O.

Following the general protocol D, using 19 µL of 3-methoxybenzaldehyde (0.15 mmol), 31.23 mg of 4-cyanopyridine (0.30 mmol), 52.25 µL of DIPEA in the first step and 115 mg of $KMnO_4$ in the second step, 29.0 mg (90% yield) of **5c** were obtained as yellowish oil, isolated after column chromatography using silica gel and DCM/MeOH 95:5 as eluent.

M.p. 111-115 °C.

^{1}H NMR (500 MHz, $CDCl_3$) δ 8.81 (d, *J* = 6.0 Hz, 2H), 7.60 – 7.55 (m, 2H), 7.40 (t, *J* = 7.9 Hz, 1H), 7.39 – 7.37 (m, 1H), 7.32 (dt, *J* = 7.6, 1.2 Hz, 1H), 7.18 (ddd, *J* = 8.2, 2.7, 1.1 Hz, 1H), 3.87 (s, 3H).

^{13}C NMR (126 MHz, $CDCl_3$) δ 195.1 (C), 160.0 (CH), 150.5 (2 CH), 144.6 (C), 137.3 (C), 129.7 (CH), 123.2 (CH), 122.3 (CH), 120.2 (CH), 114.3 (2 CH), 55.7 (CH_3).

GC- MS: calc for $C_{13}H_{11}NO_2$ 213.0790, found 213.0790, retention time 15.63 min.

5d: (3-Methylthiophen-2-yl)(pyridin-4-yl)methanone

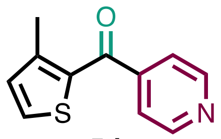

5d

Conditions: First step, flow rate: 333 µL/min, residence time: 30 min, light intensity: 44 Watt, solvent: MeCN. Second step, flow rate: 1.52 mL/min, residence time: 7.57 min, solvent: MeCN/H_2O.

Following the general protocol D, using 16.17 µL of 3-methyl-2-thiophenecarboxaldehyde (0.15 mmol), 31.23 mg of 4-cyanopyridine (0.30 mmol), 52.25 µL of DIPEA in the first step and 97 mg of $KMnO_4$ in the second step, 21.2 mg (70% yield)) of **5d** were obtained as yellowish oil, isolated after column chromatography using silica gel and DCM/MeOH 95:5 as eluent.

^{1}H NMR (500 MHz, CDCl$_3$) δ 8.79 – 8.77 (m, 2H), 7.62 – 7.59 (m, 2H), 7.56 (d, *J* = 5.0 Hz, 1H), 7.05 (d, *J* = 4.4 Hz, 1H), 2.55 (s, 3H).

^{13}C NMR (126 MHz, CDCl$_3$) δ 187.8 (C), 150.4 (2 CH), 147.9 (C), 146.9 (C), 133.7 (C), 132.9 (CH), 132.5 (CH), 122.2 (2 CH), 17.1 (CH$_3$).

GC- MS: calc for $C_{11}H_9NOS$ 203.0405, found 203.0405, retention time 15.18 min

5e: Pyridin-3-yl(pyridin-4-yl)methanone

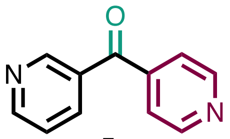

5e

Flow optimized conditions: First step, flow rate: 333 µL/min, residence time: 30 min, light intensity: 44 Watt, solvent: MeCN. Second step, flow rate: 1.52 mL/min, residence time: 7.57 min, solvent: MeCN/H_2O.

Following the general protocol D, using 14 µL of 3-pyridinecarboxaldehyde (0.15 mmol), 31.23 mg of 4-cyanopyridine (0.30 mmol), 52.25 µL of DIPEA in the first step and 97 mg of $KMnO_4$ in the second step, 15.2 mg (56% yield) of **5e** were obtained as yellowish solid, isolated after column chromatography using silica gel and DCM/MeOH 95:5 as eluent. The spectroscopic data are in line with the previous reported in literature.[9]

M.p. 70-73 °C.

^{1}H NMR (500 MHz, CDCl$_3$) δ 9.01 (d, *J* = 3.1 Hz, 1H), 8.86 (dt, *J* = 4.3, 1.9 Hz, 3H), 8.15 (dt, *J* = 7.9, 2.0 Hz, 1H), 7.61 – 7.59 (m, 2H), 7.49 (dd, *J* = 8.3, 4.4 Hz, 1H).

^{13}C NMR (126 MHz, CDCl$_3$) δ 193.8 (C), 154.0 (CH), 151.2 (CH), 150.9 (2 CH), 143.3 (C), 137.3 (CH), 131.7 (C), 123.8 (CH), 122.8 (2 CH).

GC- MS: calc for $C_{11}H_8N_2O$ 184.0637, found 184.0637, retention time 14.92 min.

Unsuccessful substrates

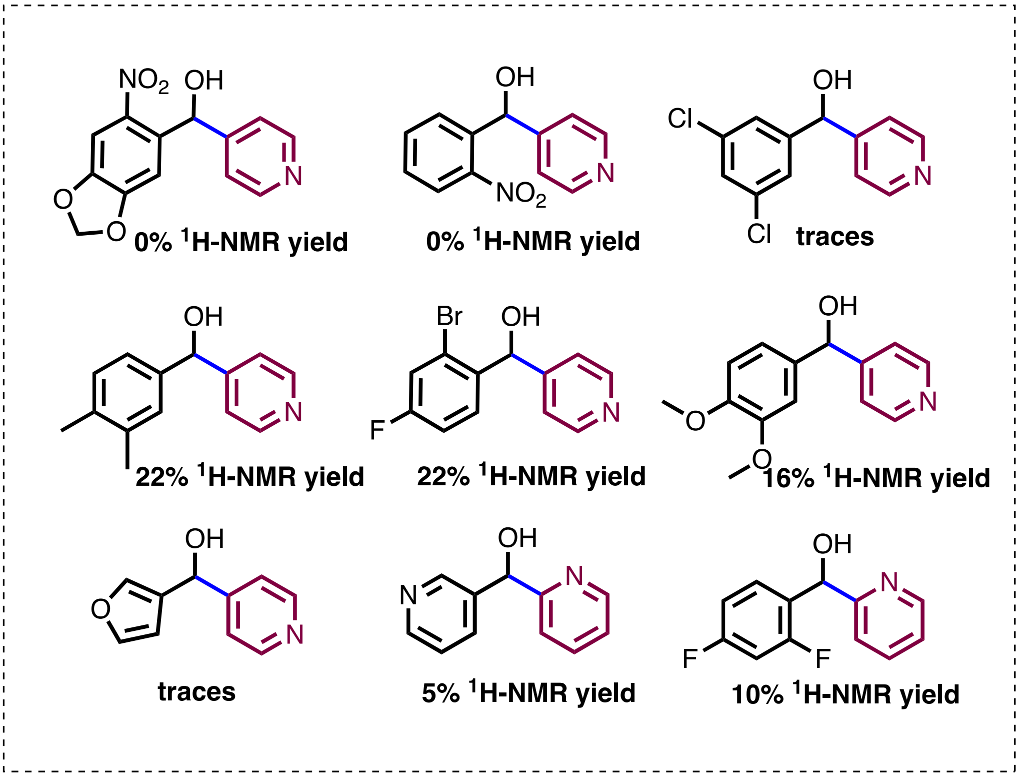

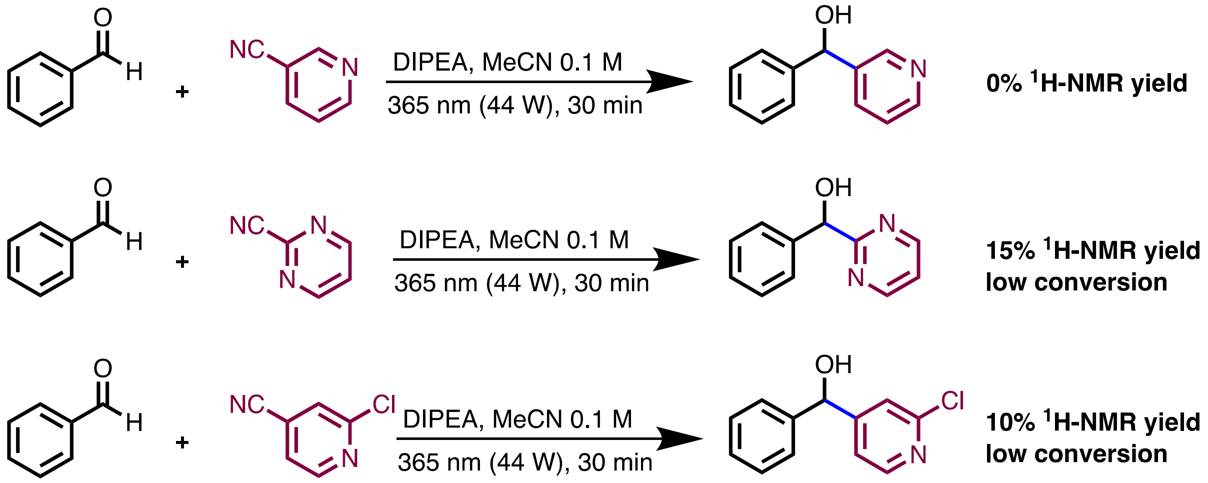

References

1. Zhang, X.; Yang, C.; Gao, H.; Wang, L.; Guo, L.; Xia, W. Reductive Arylation of Aliphatic and Aromatic Aldehydes with Cyanoarenes by Electrolysis for the Synthesis of Alcohols. *Org. Lett.* **2021**, 23, 9, 3472–3476.

2. Zhong, L-J; Wang, H-Y; Ouyang, X-H; Li, J-H; An, D-L. Benzylic C–H Heteroarylation of N-(benzyloxy)phthalimides with Cyanopyridines Enabled by Photoredox 1,2-Hydrogen Atom Transfer. *Chem. Commun*., **2020**, 56, 8671-8674.

3. Trécourt, F.; Breton, G.; Bonnet, V.; Mongin, F.; Marsais, F; Quéguiner, G. New Syntheses of Substituted Pyridines via Bromine–Magnesium Exchange. *Tetrahedron*, 2000, 56, 1349–1360.

4. Kuriyama, M.; Nakashima, S.; Miyagi, Sato, K.; Yamamoto, K.; Onomura, O. Palladium-Catalyzed Chemoselective Anaerobic Oxidation of N- Heterocycle-Containing Alcohols. *Organic Chemistry Frontiers*, **2018**, 5(15), 2364-2369.

5. Yu, R.; Zhang, X.; Hao, F.; Zhengneng, J.; Liu, G.; Dai, G.; Wu, J. Cobalt-Promoted Transfer Hydrogenation of Azaaryl Ketones by Using Formate as the Hydrogen Source. *Eur. J. Org.Chem.,* **2023**, 26, e202300071

6. Wang, B.; Zhou, H.; Lu, G.; Liu, Q.; Jiang, X. Bifunctional Oxo-Tethered Ruthenium Complex CatalyzedAsymmetric Transfer Hydrogenation of ArylN Heteroaryl Ketones. *Org.Lett*., **2017**, 19, 2094−2097.

7. Ye, R.; Cao, Y.; Xi, X.; Liu, L.; Chen, T. Metal- and Radical-free Aerobic Oxidation of Heteroaromatic Methanes: an Efficient Synthesis of Heteroaromatic Aldehydes. *Org. Biomol. Chem.*, **2019**, 17, 4220-4224.

8. Tao, X.; Li, W.; Ma, X.; Li, X.; Fan, W.; Xie, X.; Ayad, T.; Ratovelomanana-Vidal, V.; Zhang, Z. Ruthenium-Catalyzed Enantioselective Hydrogenation of Aryl-Pyridyl Ketones. *J.Org.Chem.* **2012**, 77, 612−616.

9. Roxburgh, M; McMorran, D.; Garden, A. L.; Hanton, L. R. Orientation of Ag^{I} Ions in Coordination Architectures through Ligand Conformation and Anion Binding: from Polymeric Chains to Discrete Squares. *Eur. J. Inorg. Chem*., **2018**, 4278–4285.

Pictures of the flow equipment

Flow set-up for the synthesis of alcohol derivatives

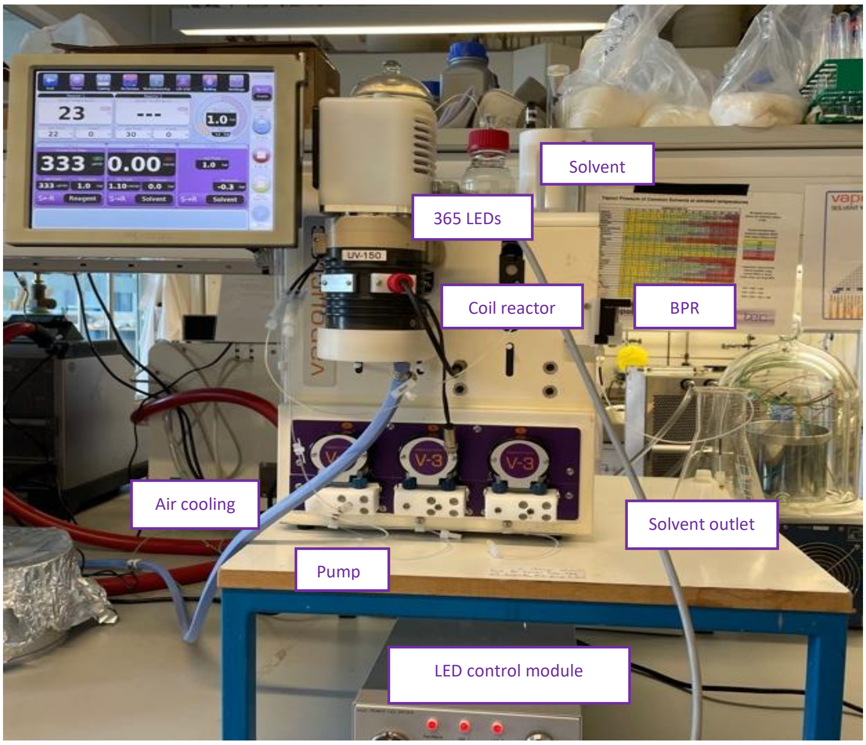

365 nm High power LEDs and 10 mL reactor coil

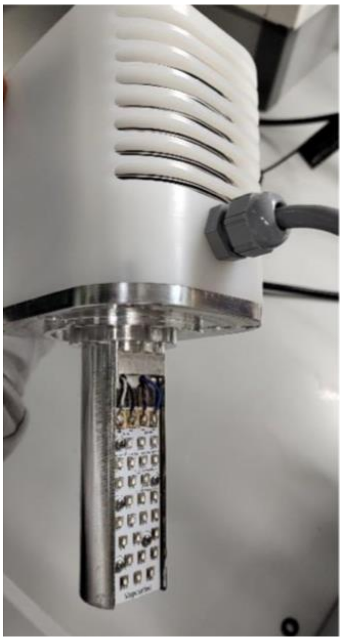

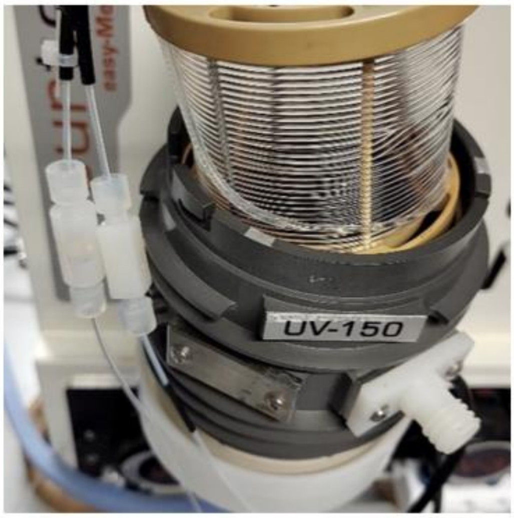

Telescoped set-up for the synthesis of ketone derivatives

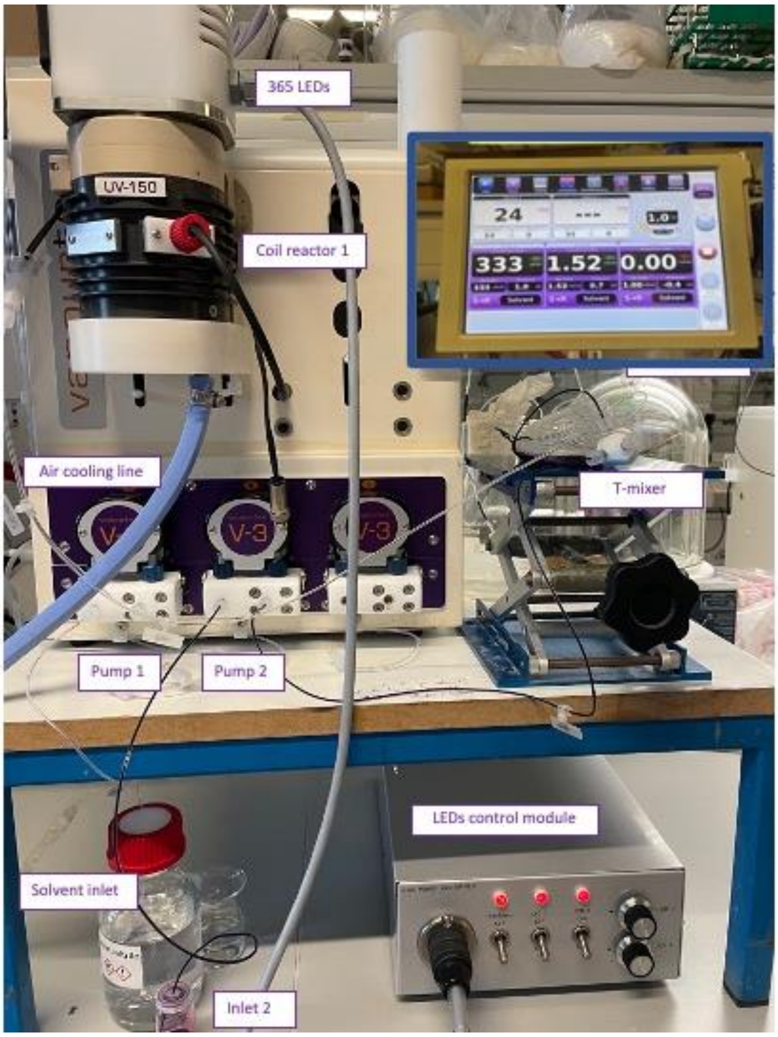

Copies of NMR Spectra

^{1}H and ^{13}C NMR spectra of compound **3a** ($CDCl_3$, 400 MHz, 101 MHz)

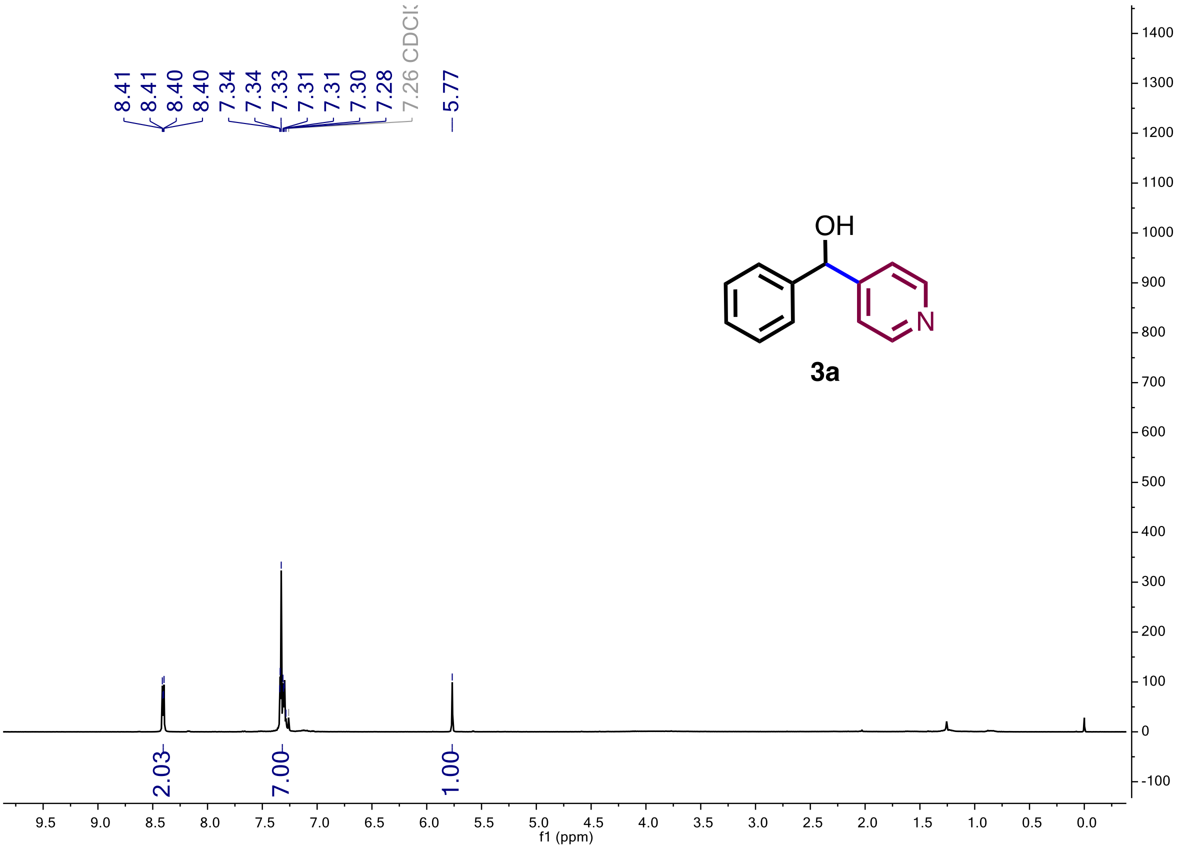

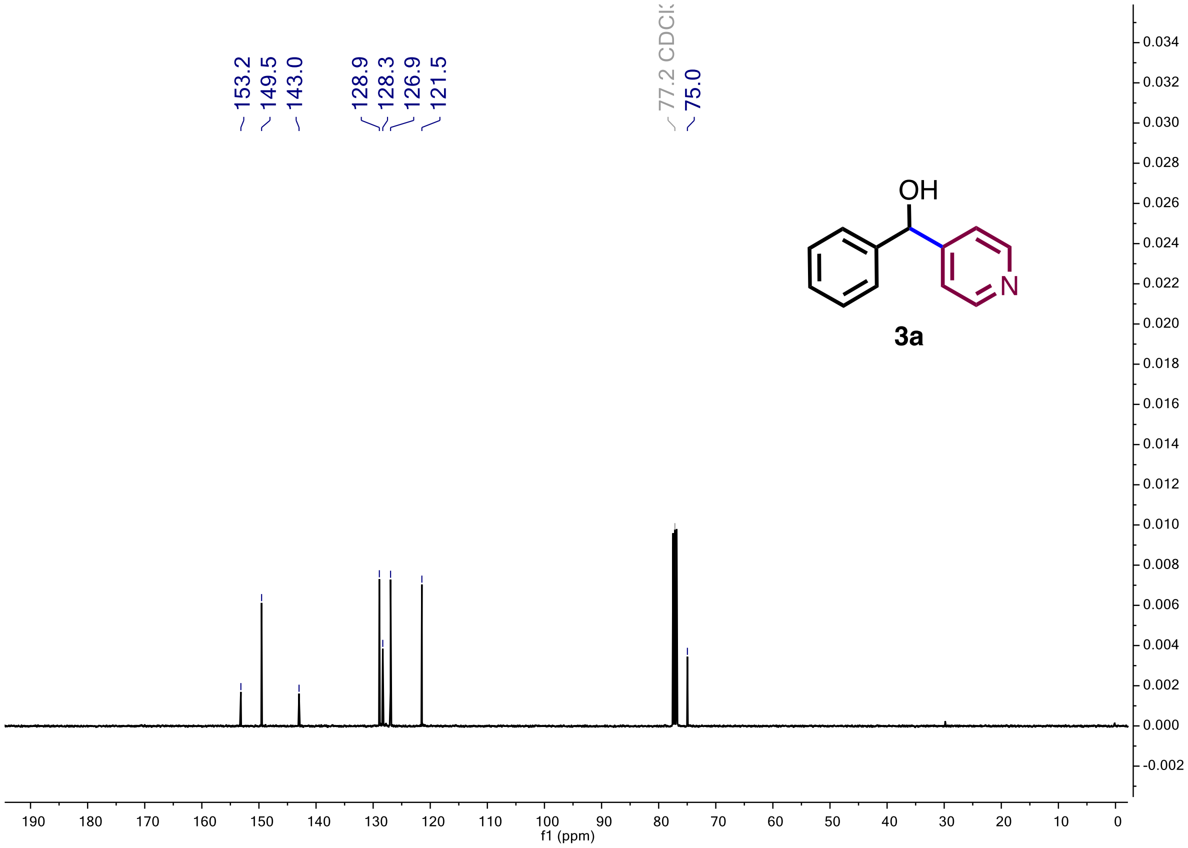

^{1}H and ^{13}C NMR spectra of compound **3b** ($CDCl_3$, 400 MHz, 101 MHz)

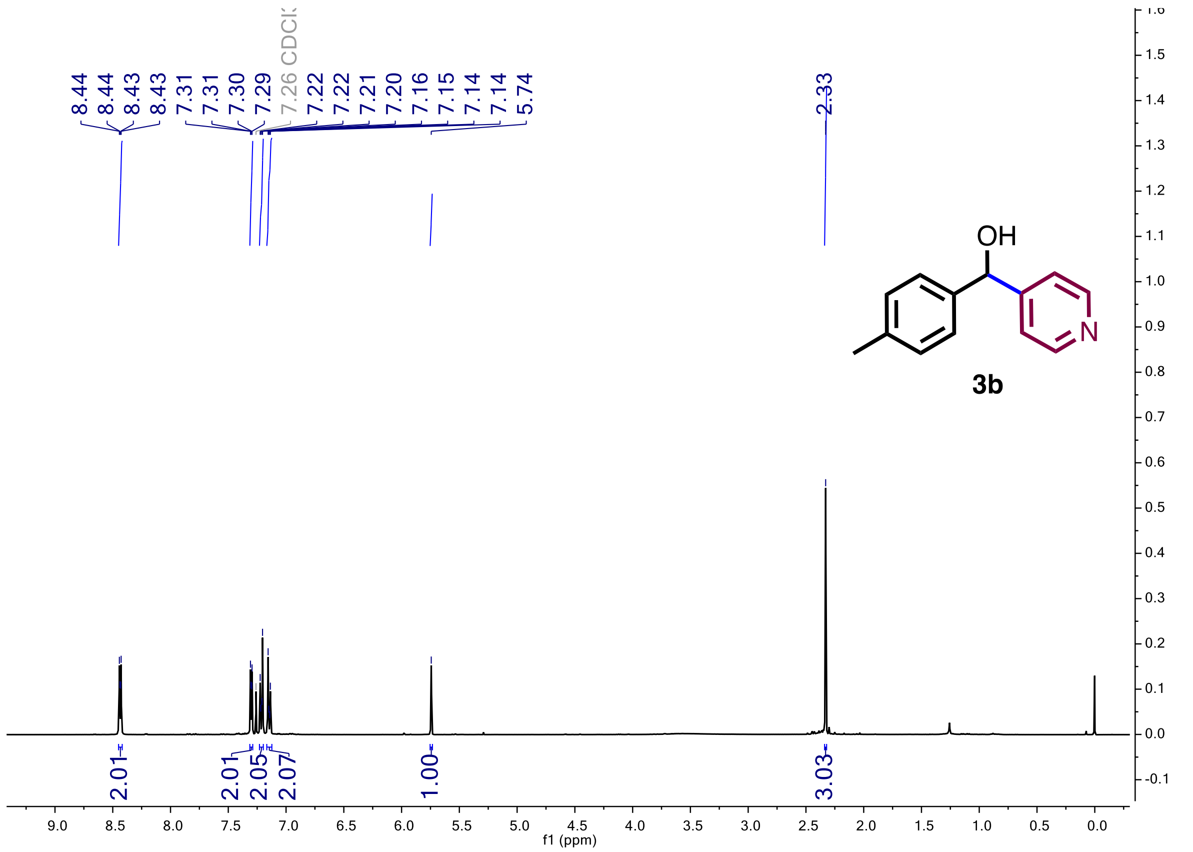

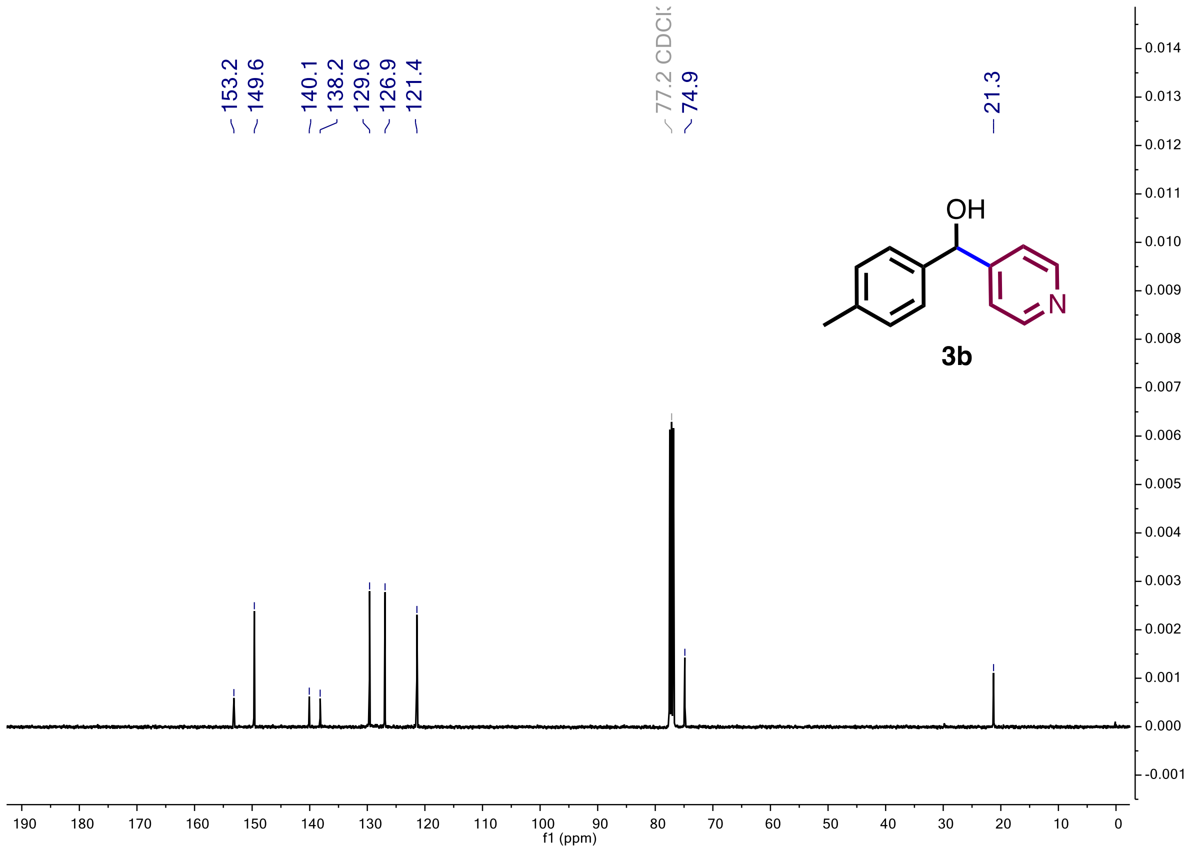

^{1}H and ^{13}C NMR spectra of compound **3c** ($CDCl_3$, 400 MHz, 101 MHz)

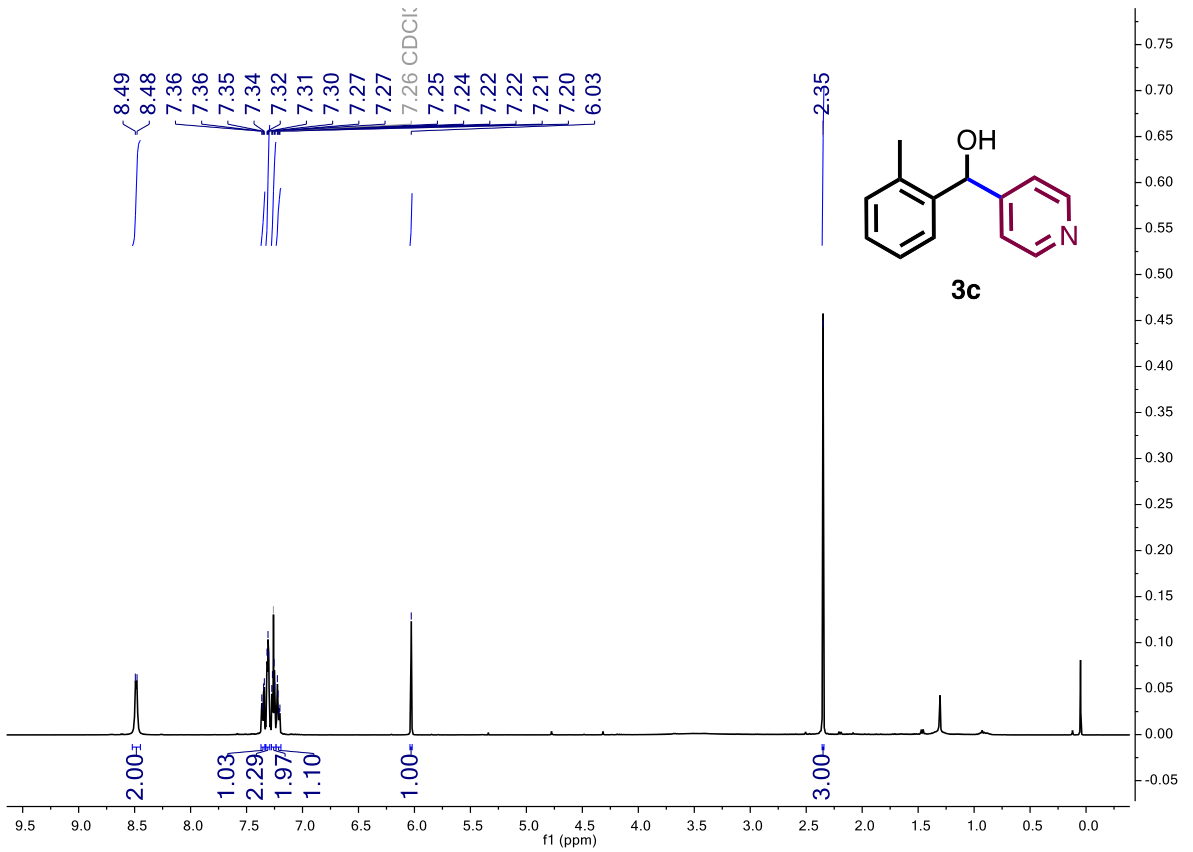

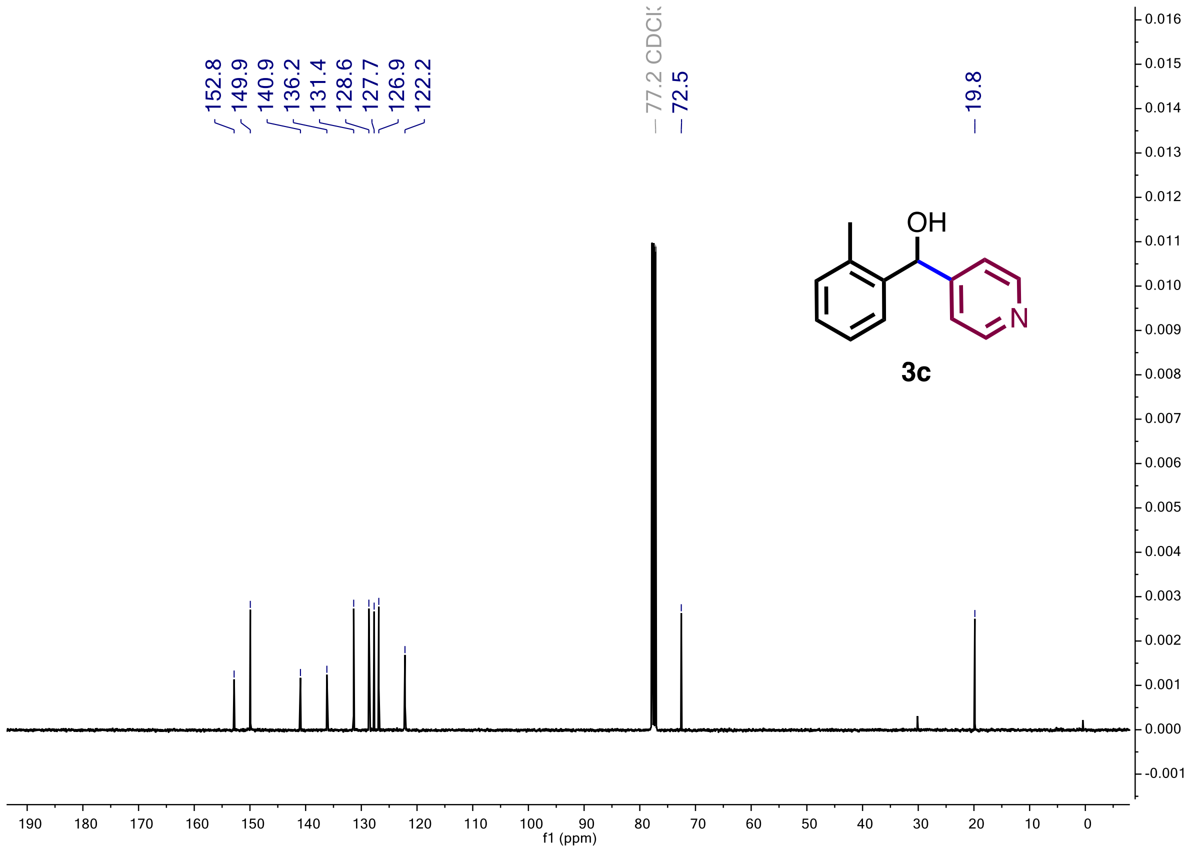

^{1}H ,^{13}C and ^{19}F NMR spectra of compound **3d** ($CDCl_3$, 400 MHz, 101 MHz, 470 MHz)

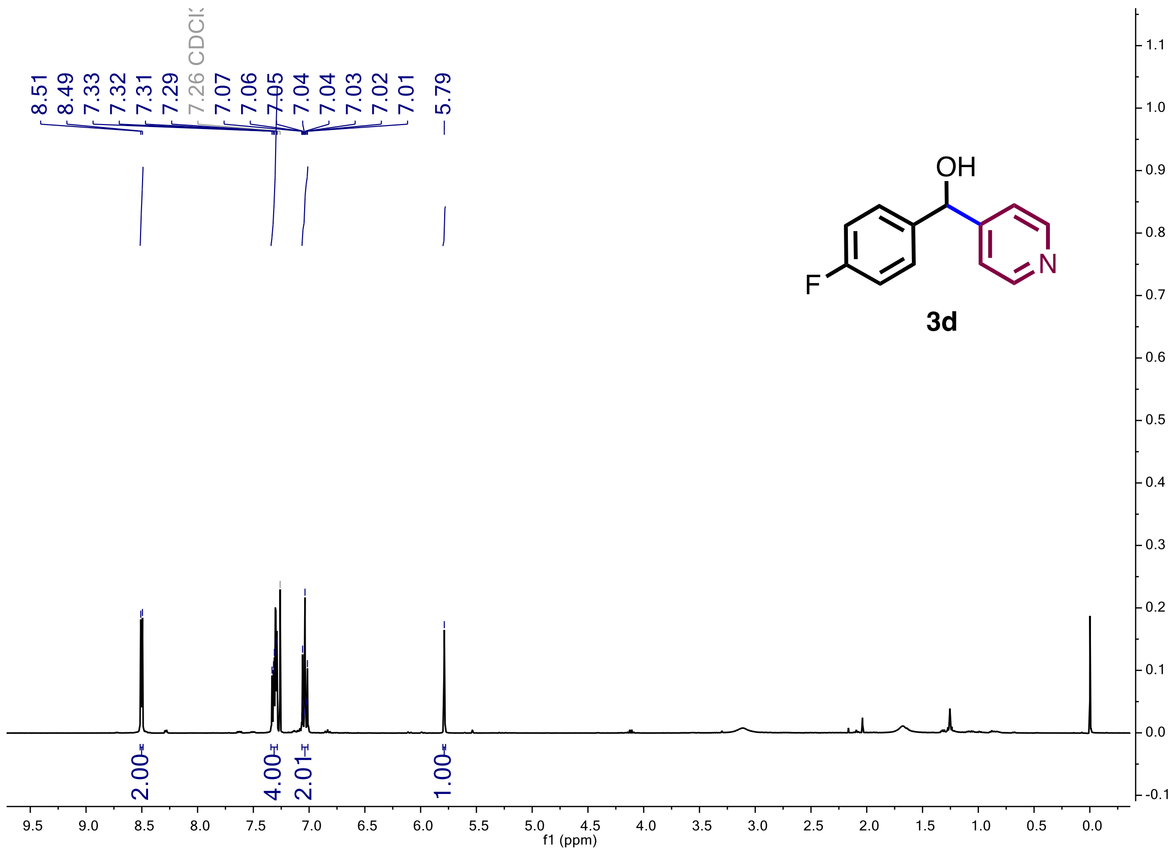

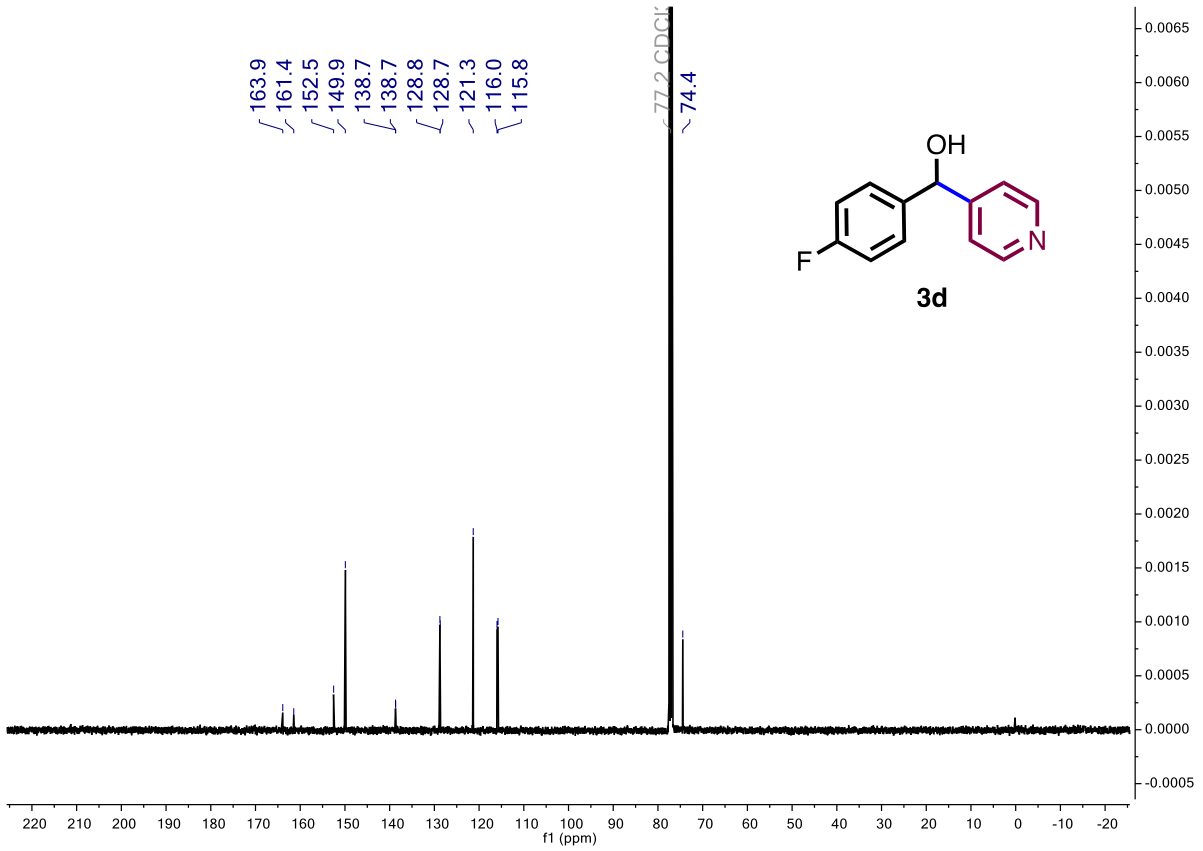

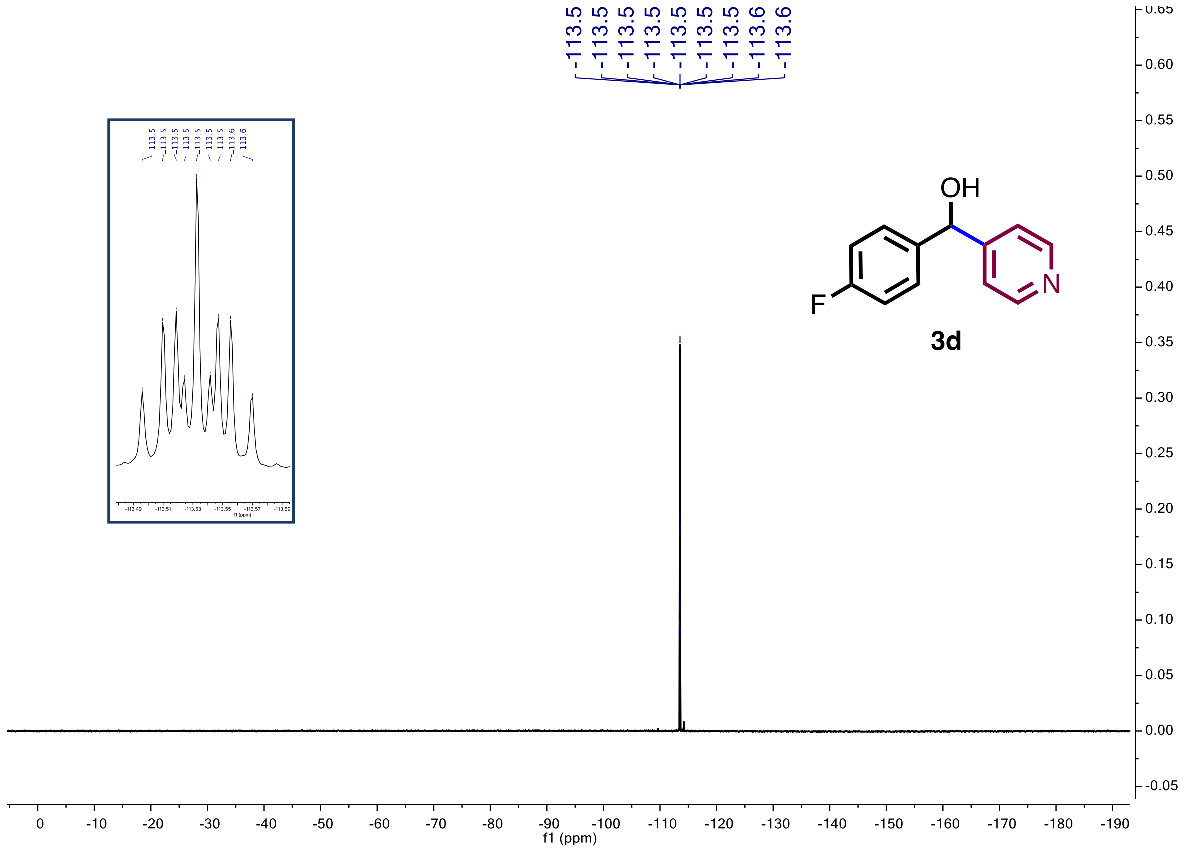

^{1}H and ^{13}C NMR spectra of compound **3e** ($CDCl_3$, 400 MHz, 101 MHz)

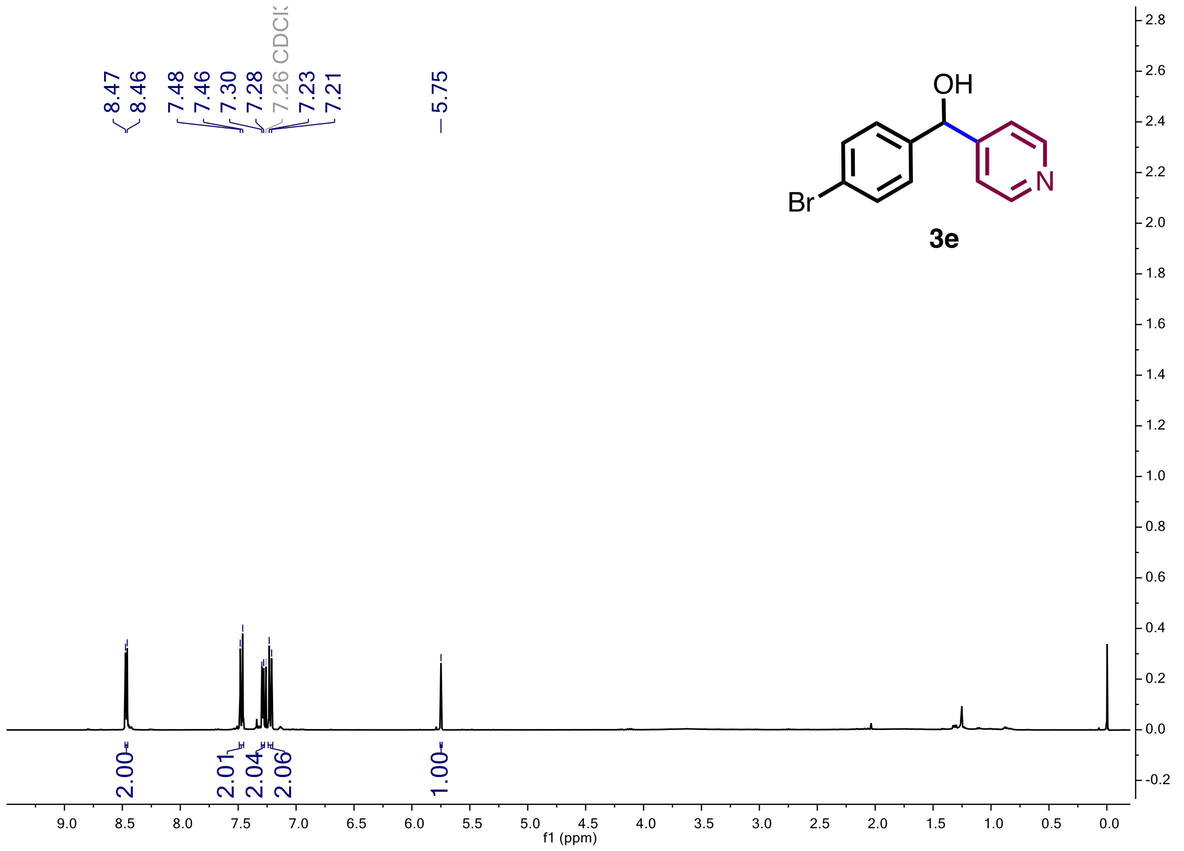

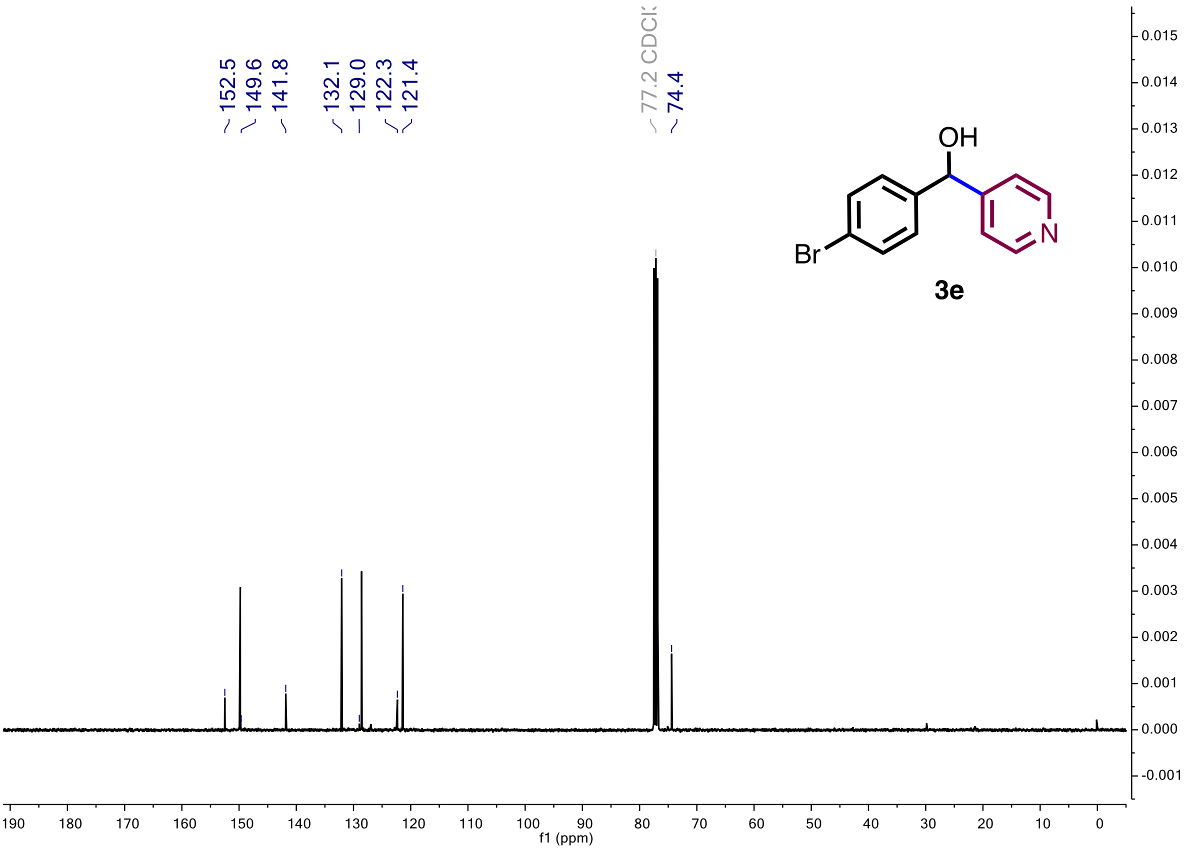

^{1}H and ^{13}C NMR spectra of compound **3f** ($CDCl_3$, 400 MHz, 101 MHz)

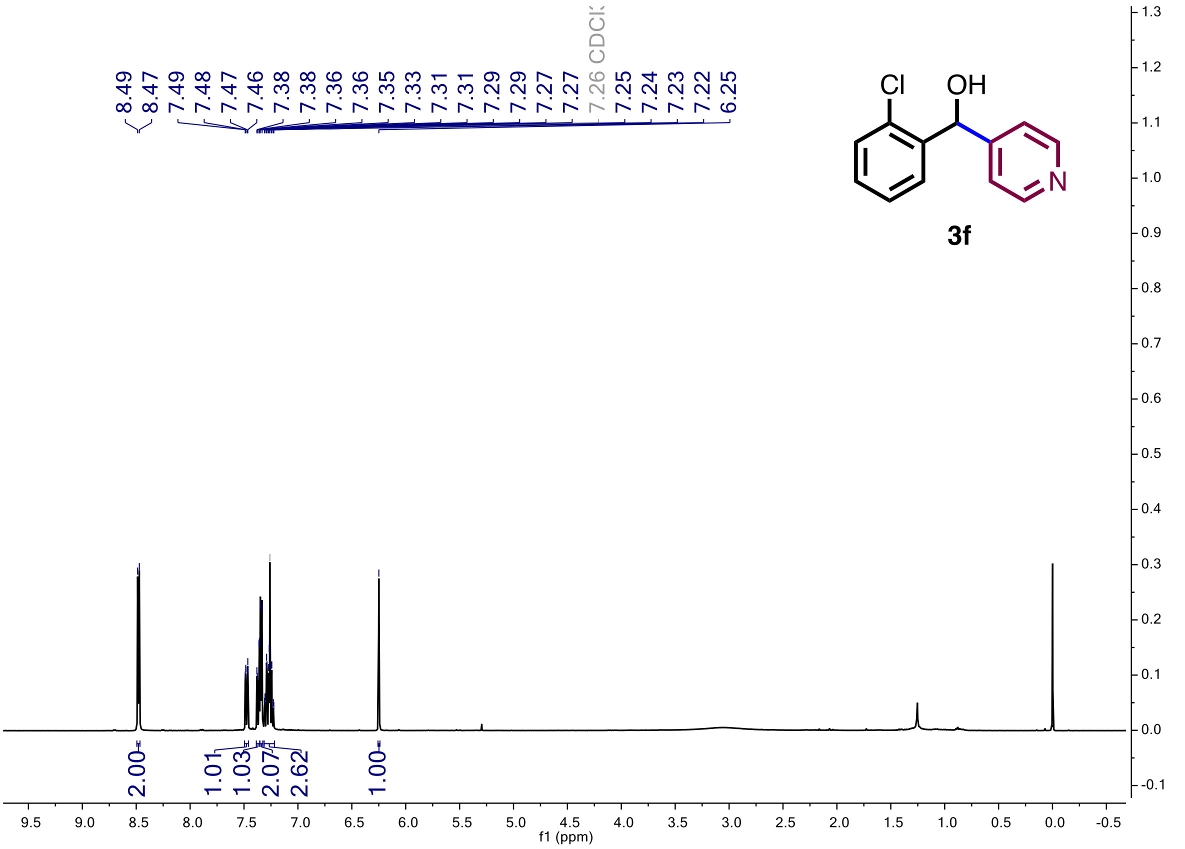

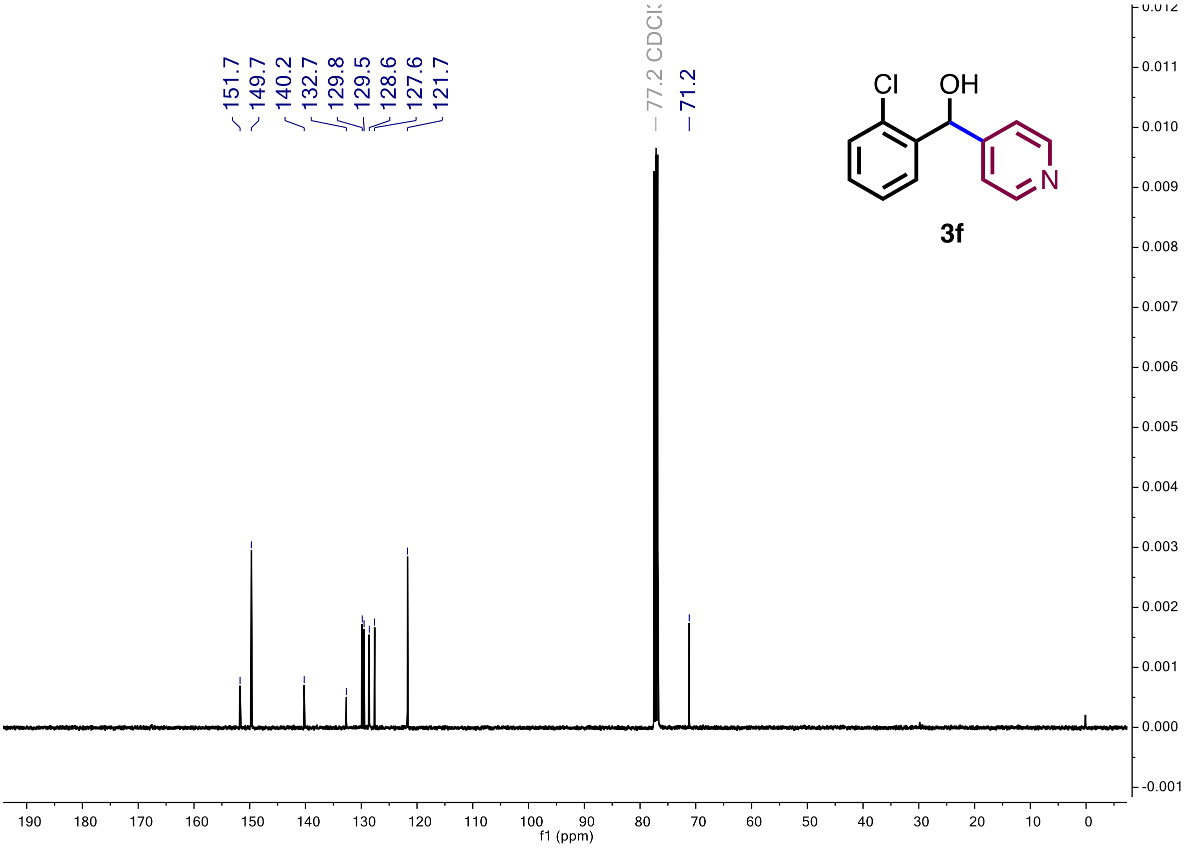

^{1}H and ^{13}C NMR spectra of compound **3g** ($CDCl_3$, 400 MHz, 101 MHz)

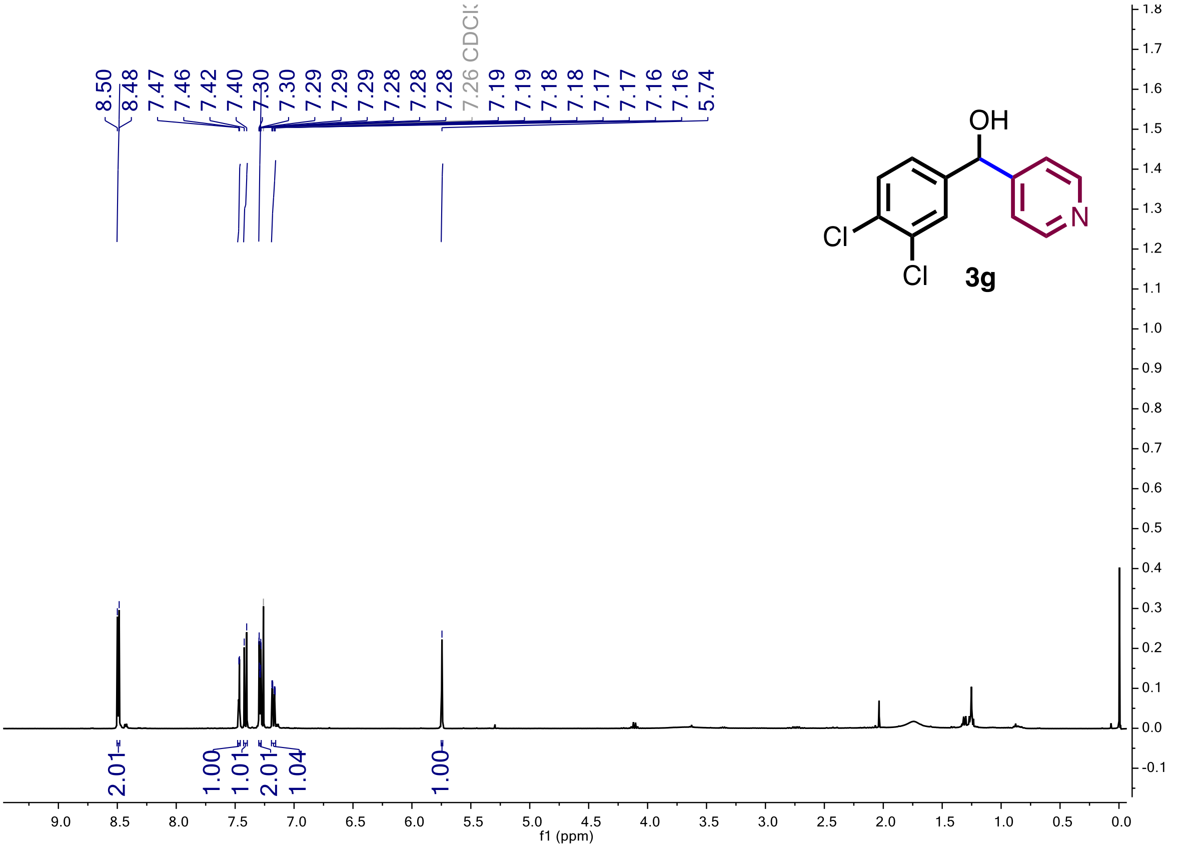

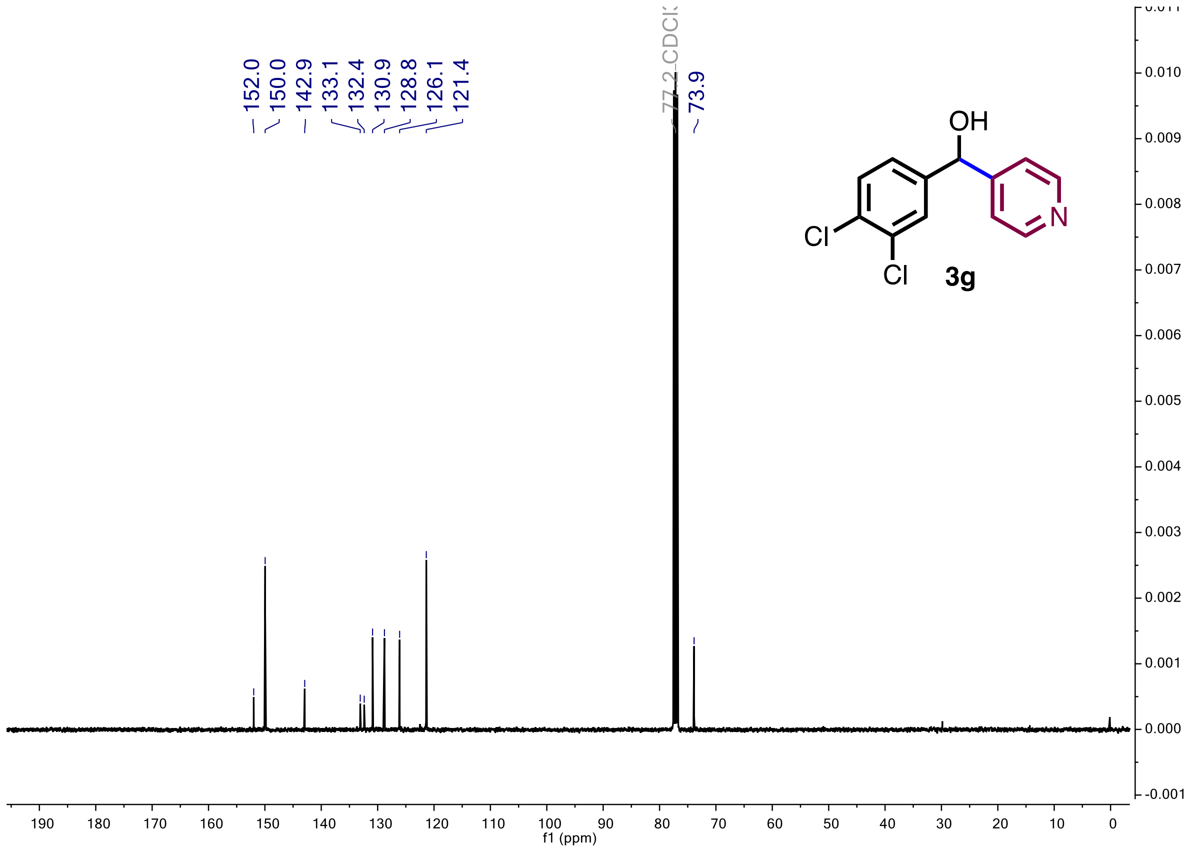

^{1}H, ^{13}C and ^{19}F NMR spectra of compound **3h** ($CDCl_3$, 400 MHz, 126 MHz, 470 MHz)

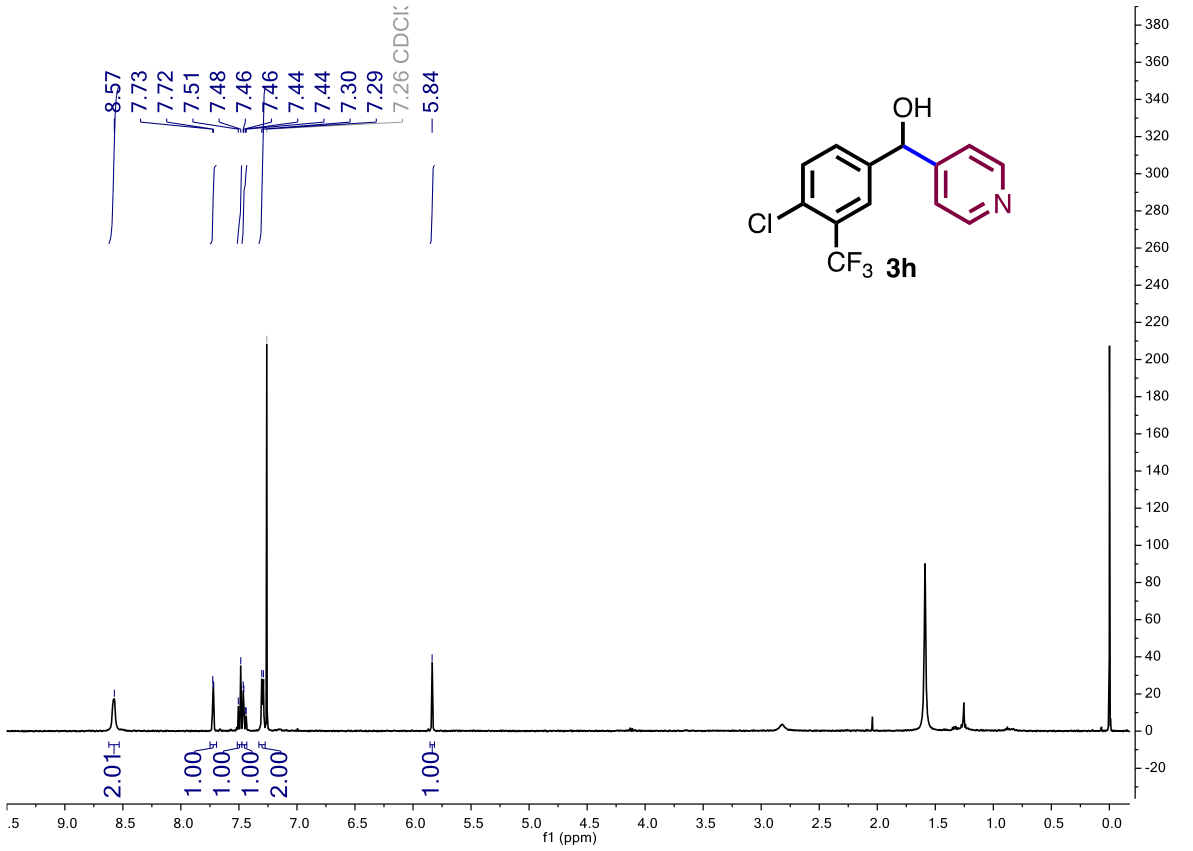

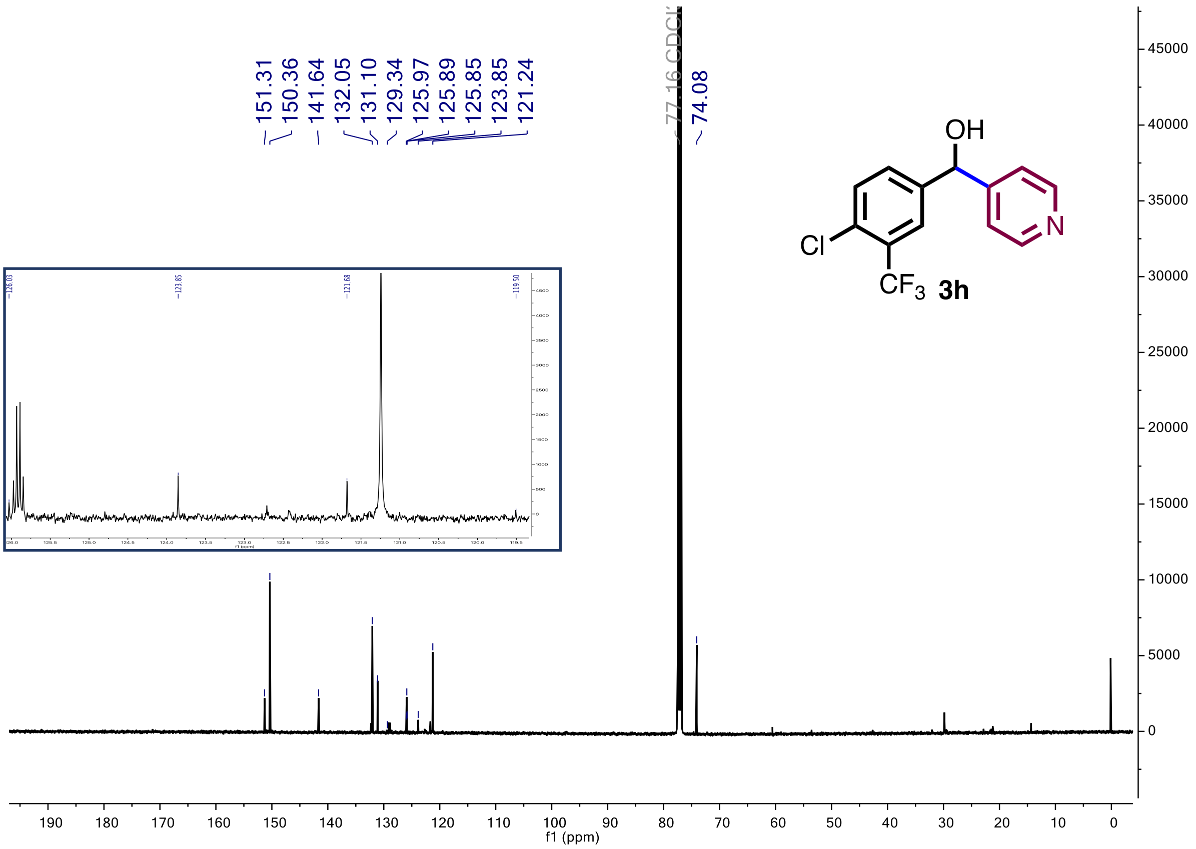
151.31
150.36
141.64
132.05
131.10
129.34
125.97
125.89
125.85
123.85
121.24
77.16 CDCl3
74.08
OH
N
Cl
CF3 3h
f1 (ppm)

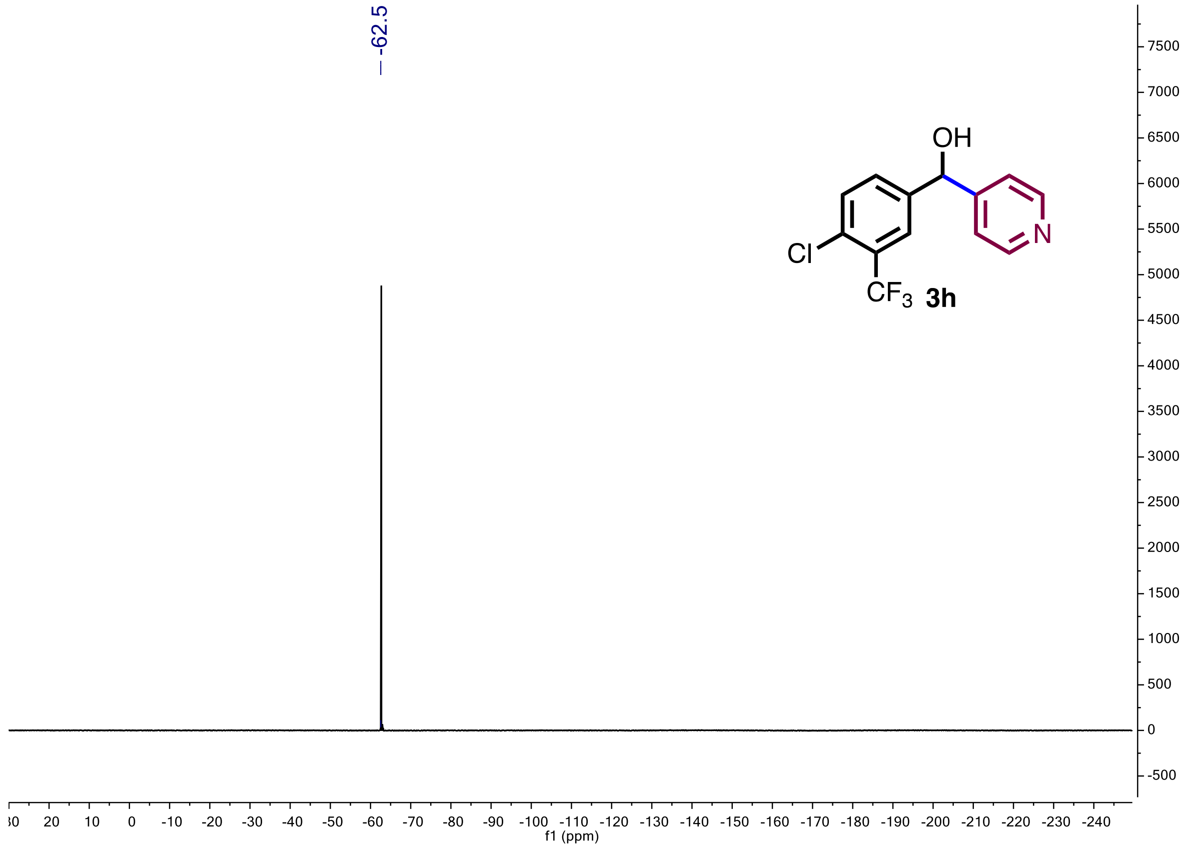
-62.5
OH
N
Cl
CF3 3h
f1 (ppm)

^{1}H, ^{13}C and ^{19}F NMR spectra of compound **3i** ($CDCl_3$, 400 MHz, 151 MHz, 470 MHz)

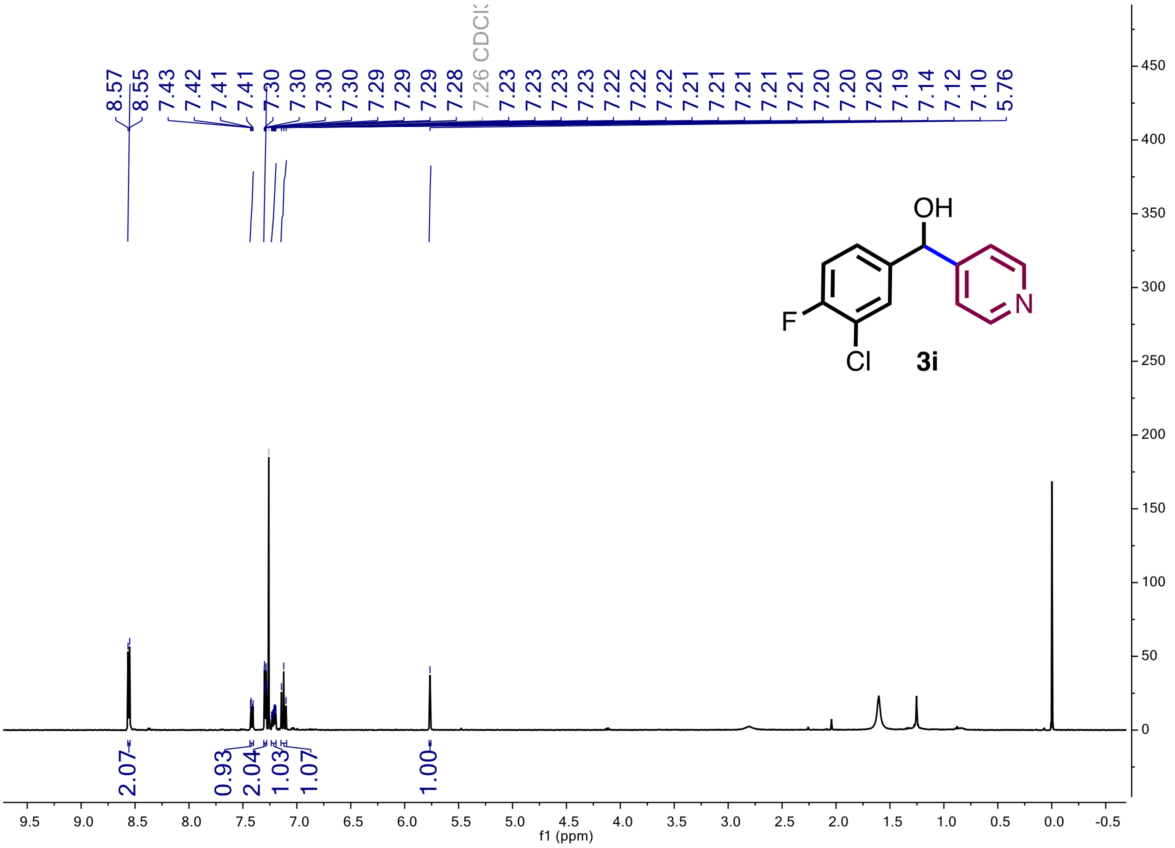

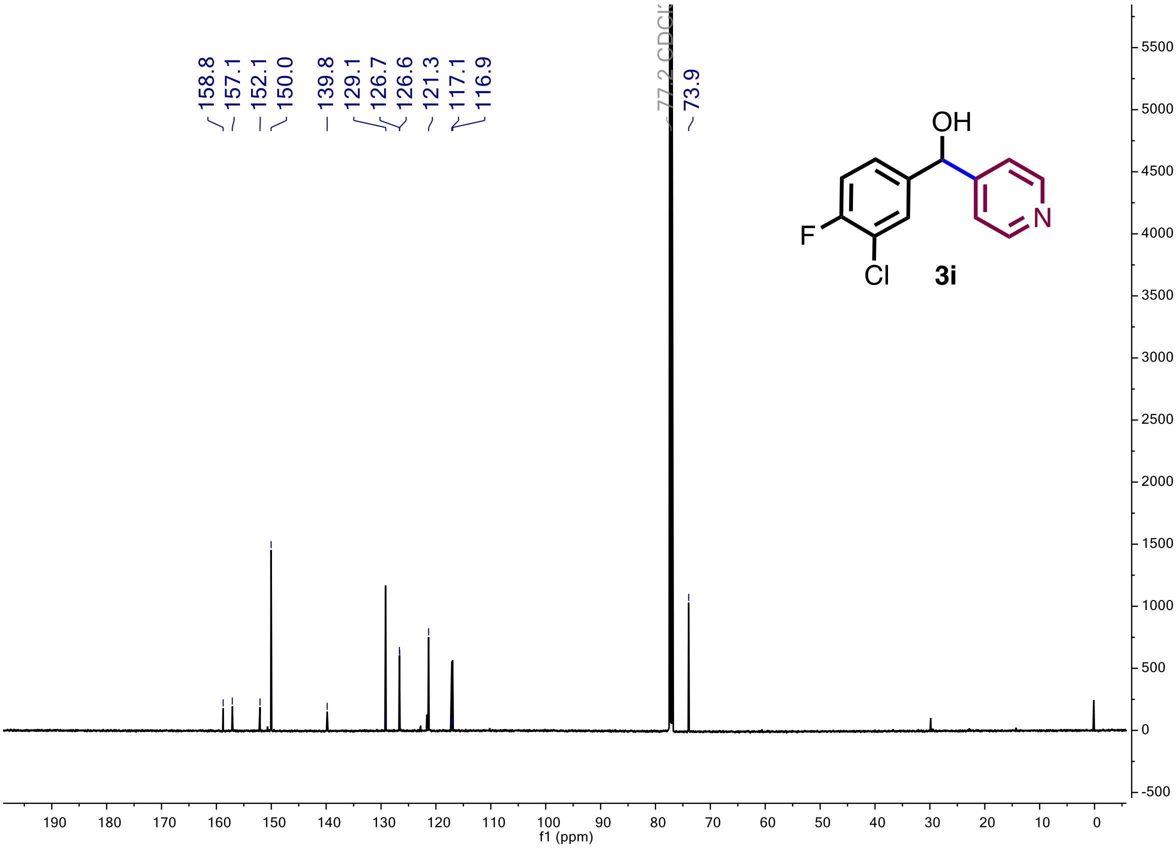

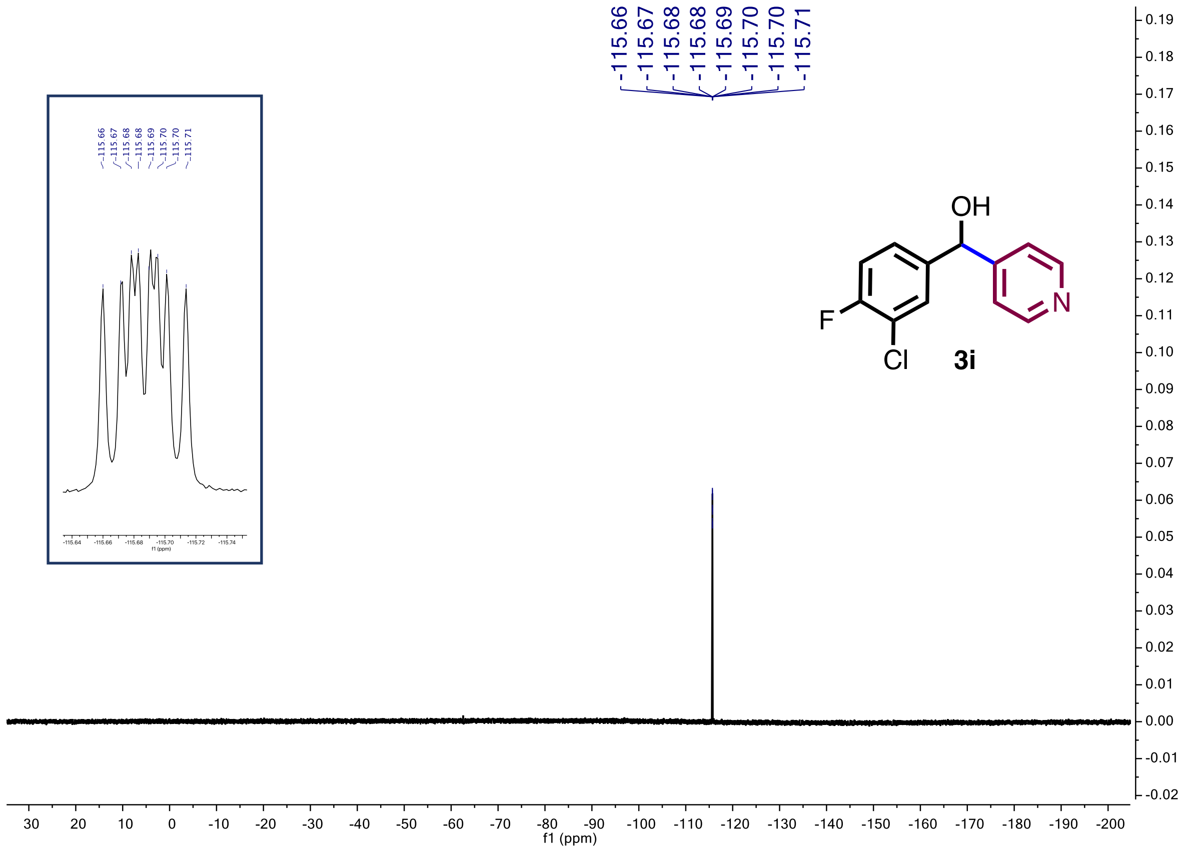

^{1}H and ^{13}C NMR spectra of compound **3j** (DMSOd$_6$, 500 MHz, 126 MHz)

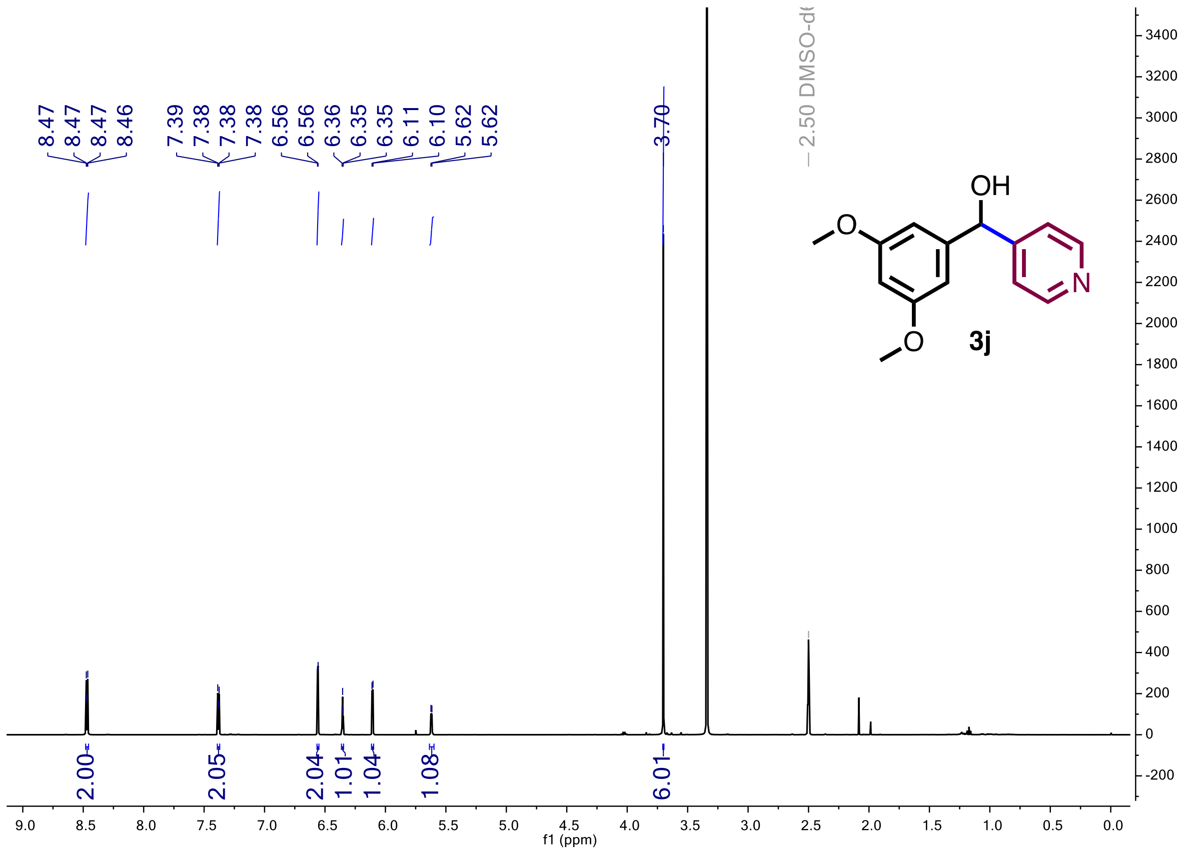

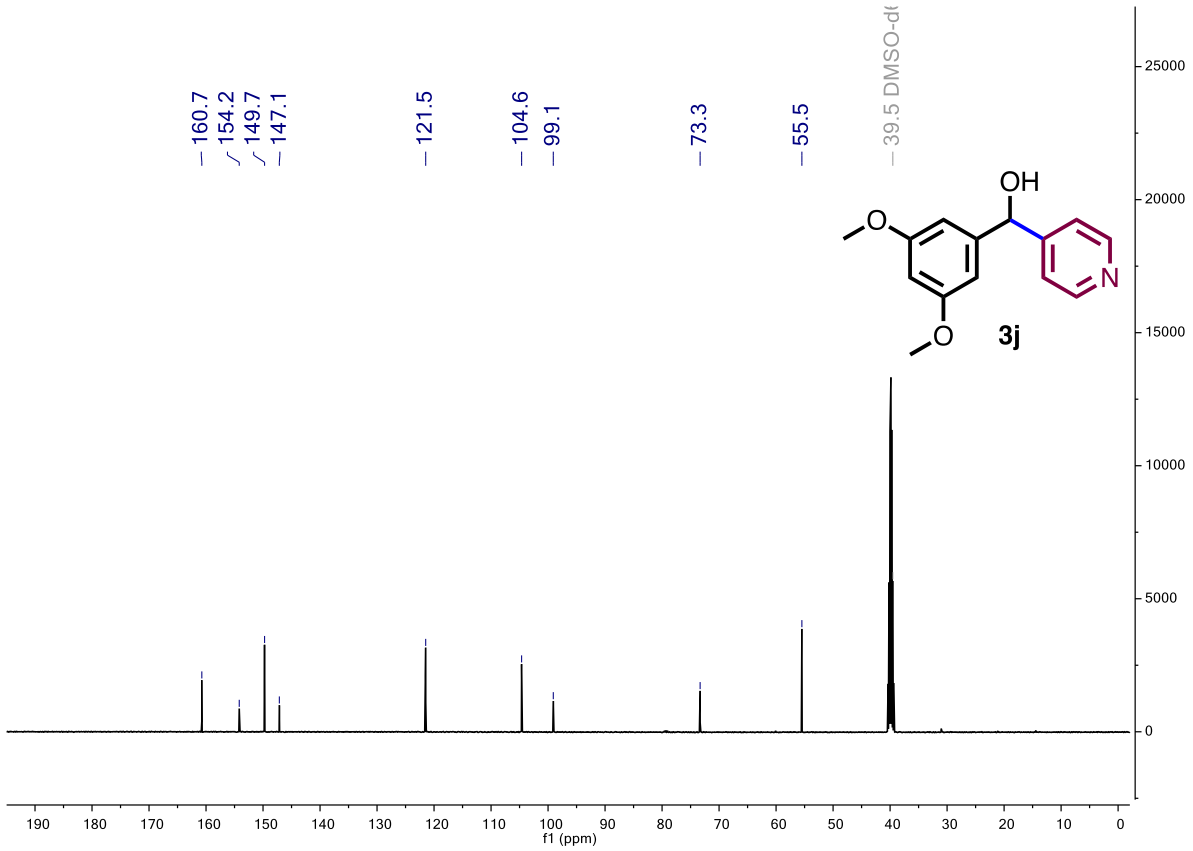

^{1}H and ^{13}C NMR spectra of compound **3k** ($CDCl_3$, 400 MHz, 101 MHz)

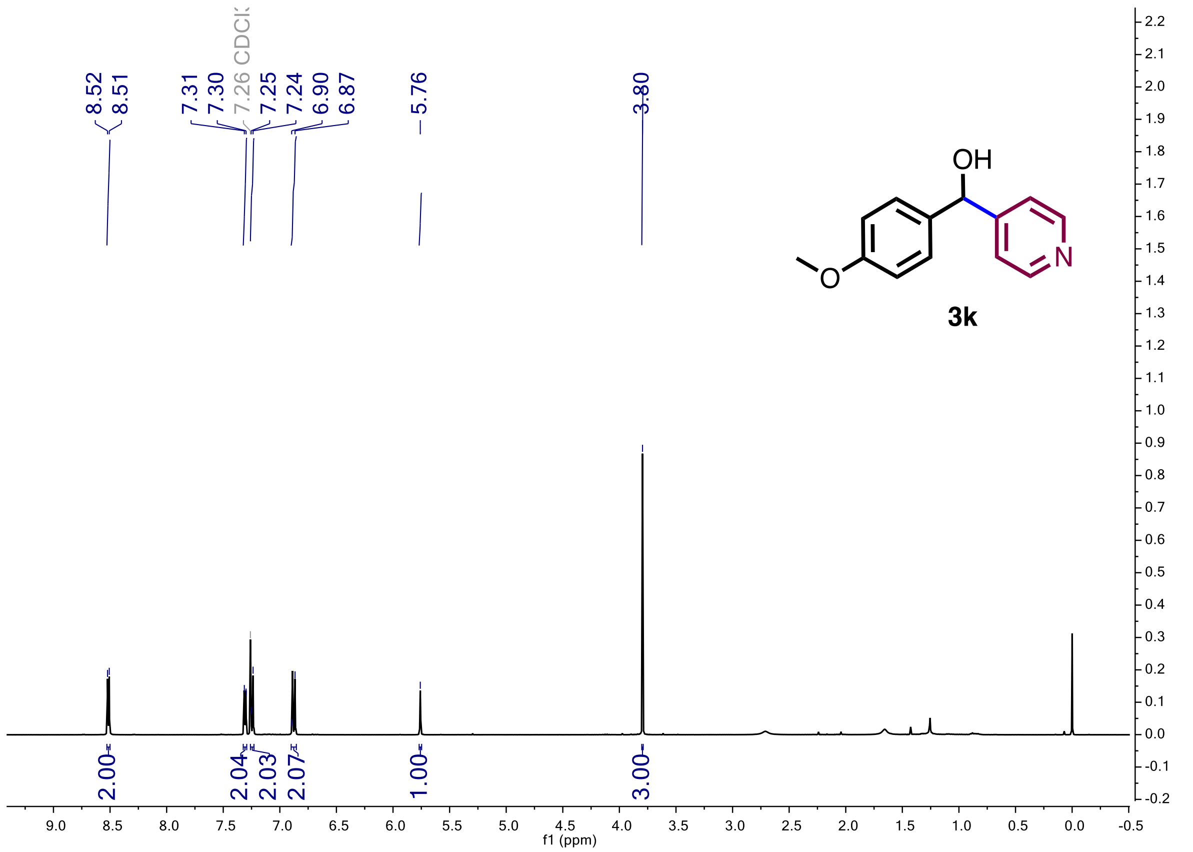

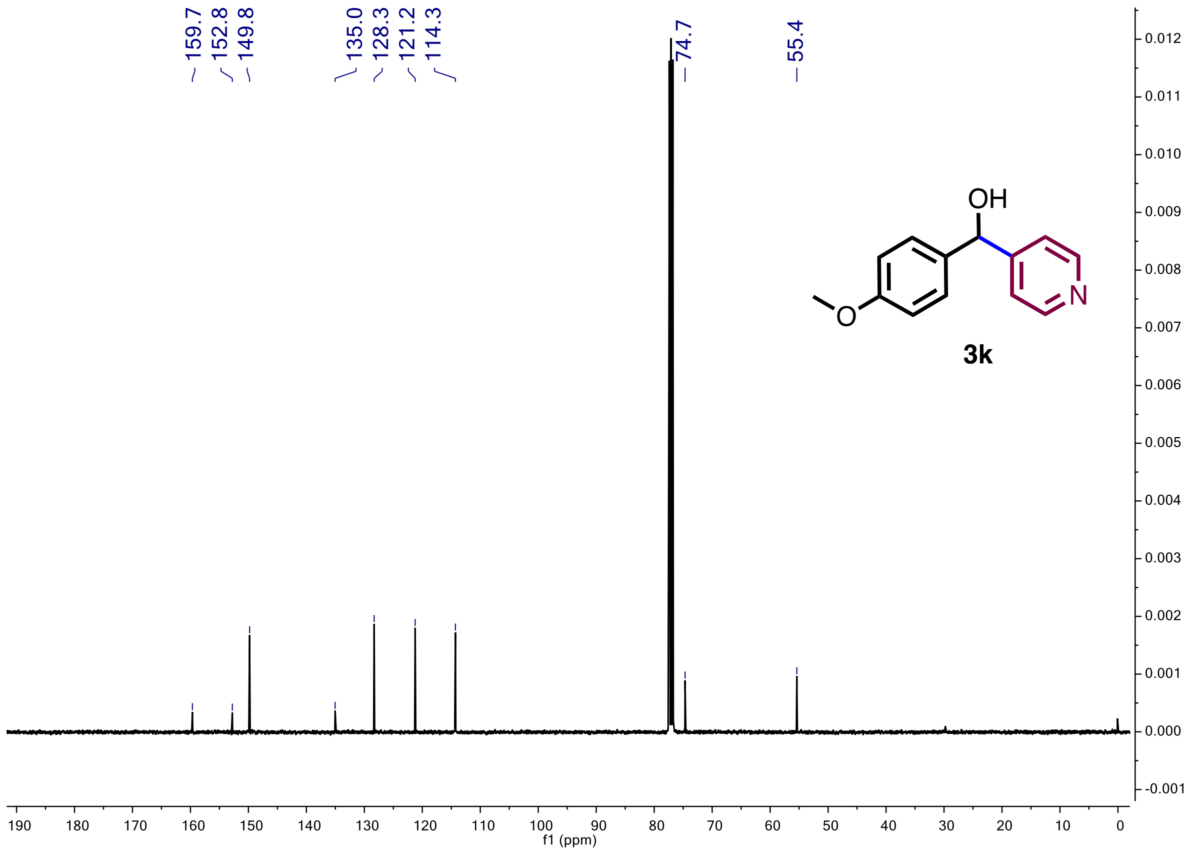

^1H and ^{13}C NMR spectra of compound **3l** ($CDCl_3$, 400 MHz, 101 MHz)

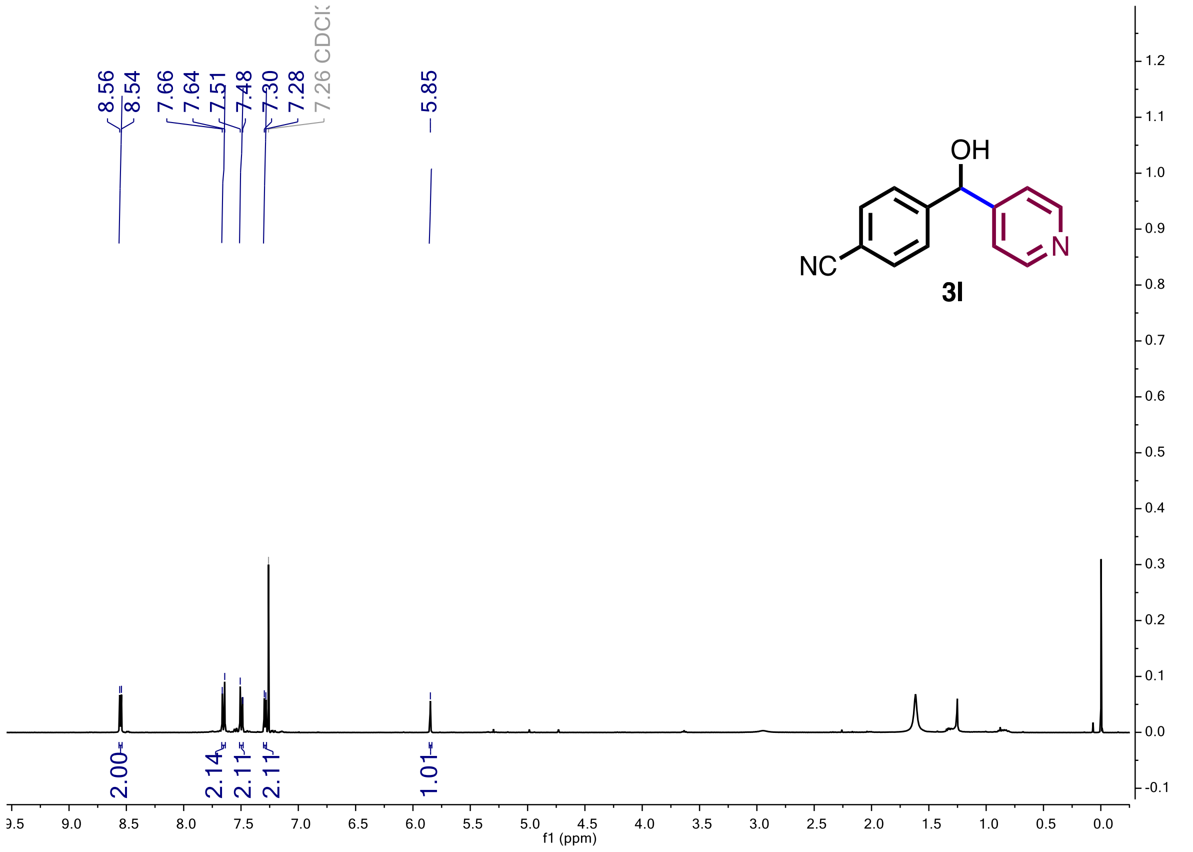

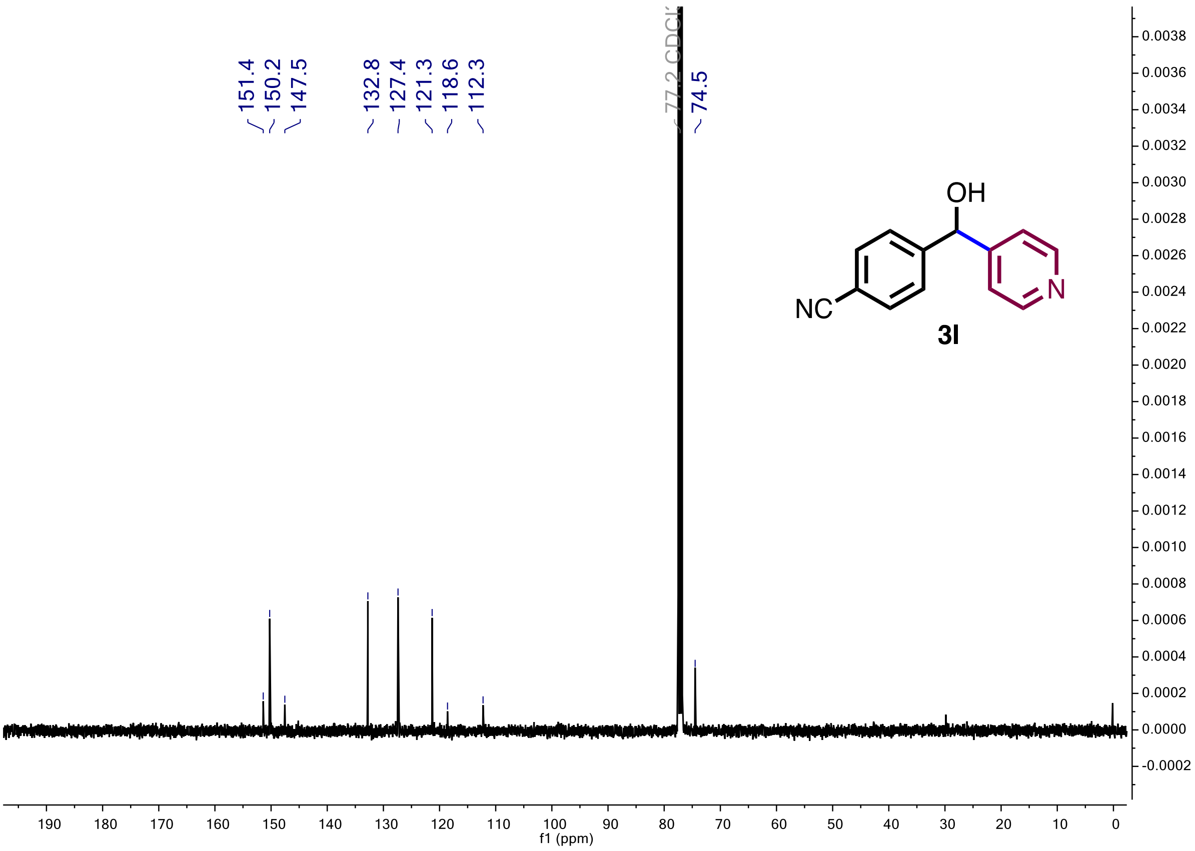

^{1}H and ^{13}C NMR spectra of compound **3m** ($CDCl_3$, 500 MHz, 126 MHz)

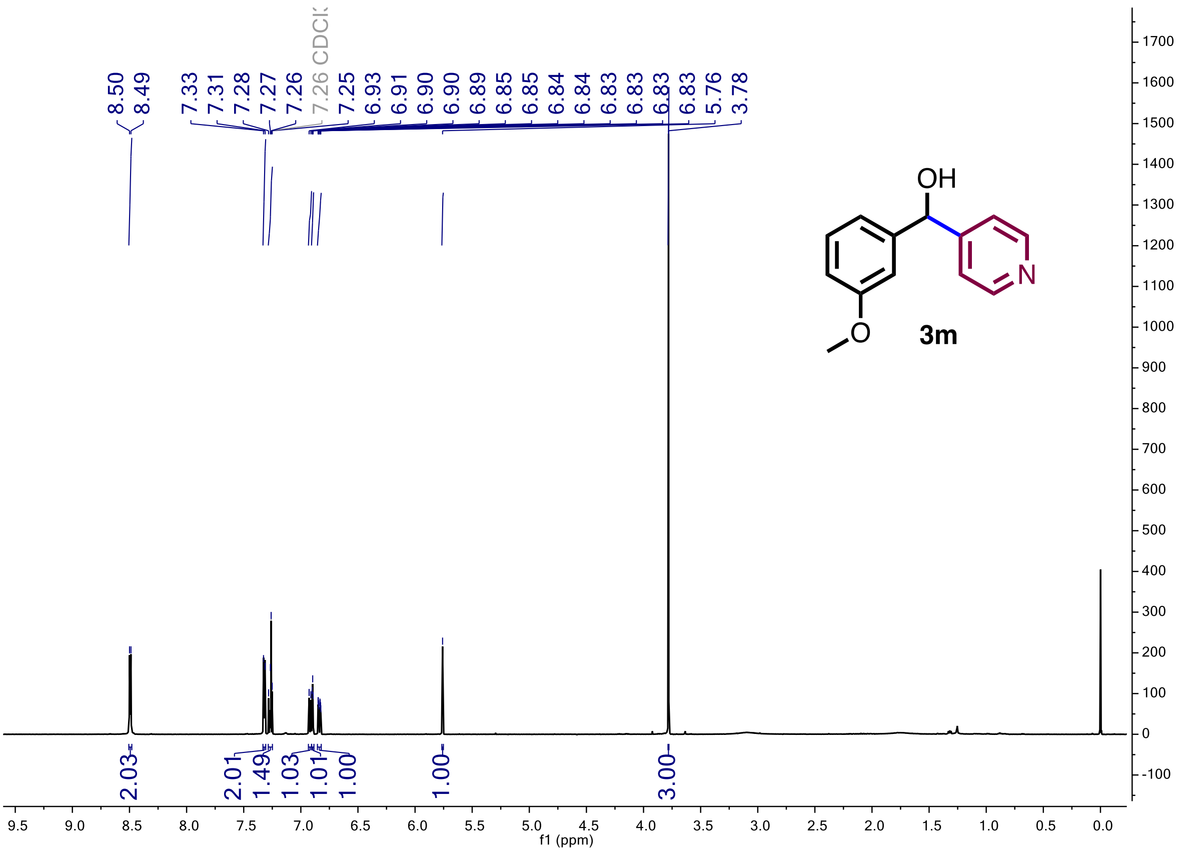

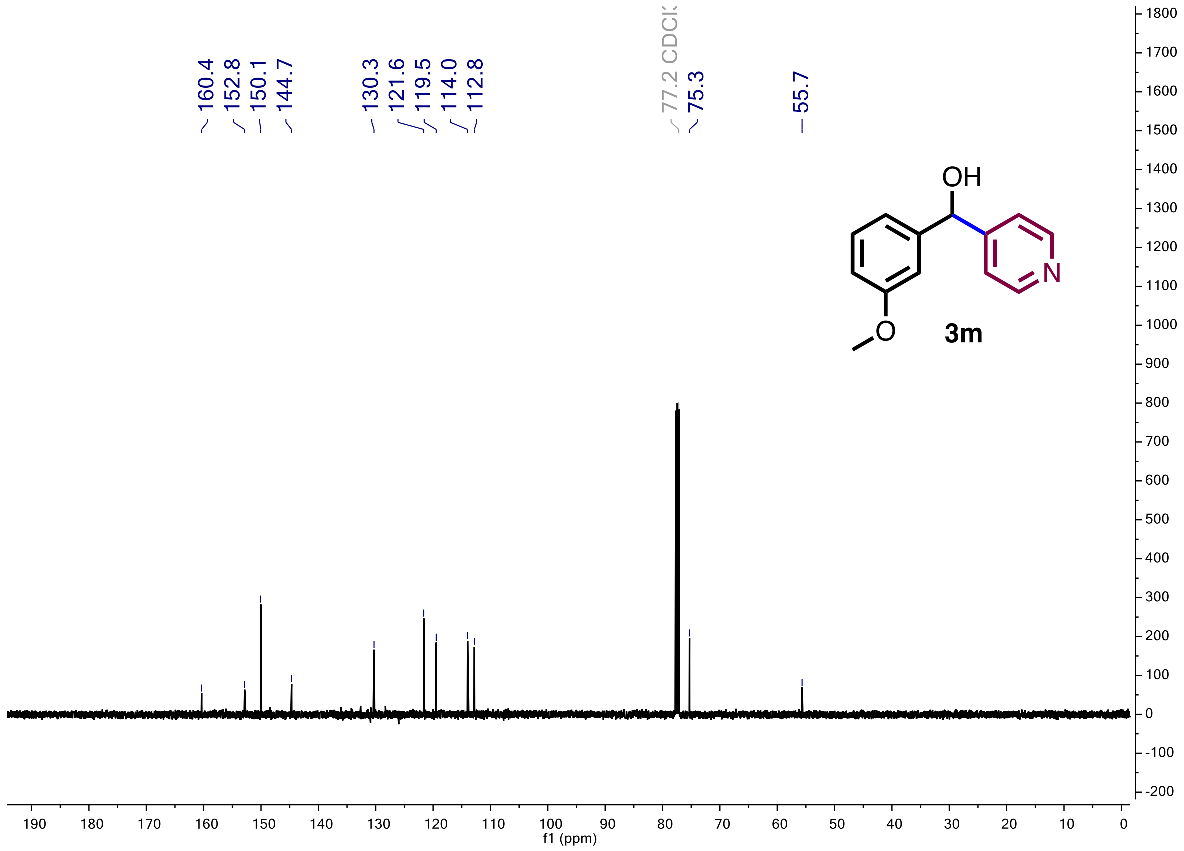

^{1}H, ^{19}F and ^{13}C NMR spectra of compound **3n** ($CDCl_3$, 400 MHz, 101 MHz, 376 MHz)

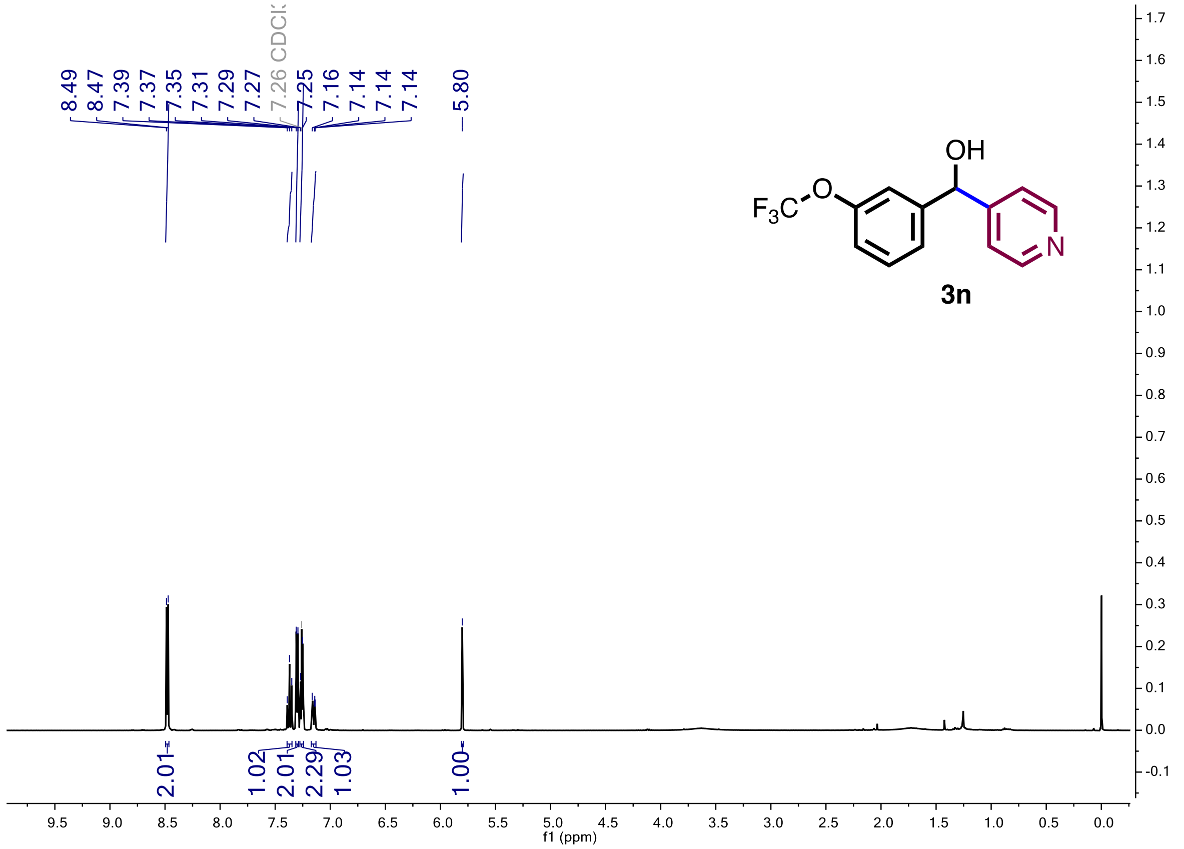

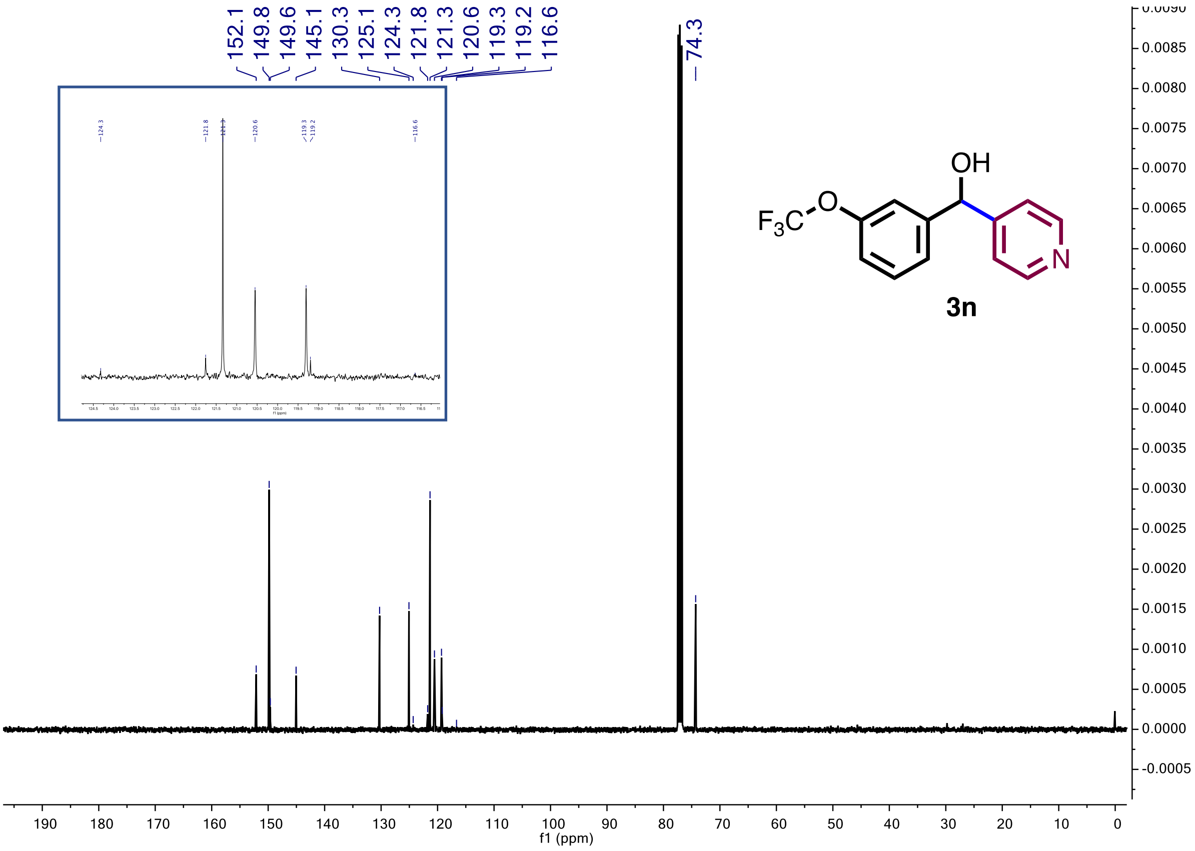
152.1
149.8
149.6
145.1
130.3
125.1
124.3
121.8
121.3
120.6
119.3
119.2
116.6
74.3
OH
F_3C
O
N
3n
f1 (ppm)

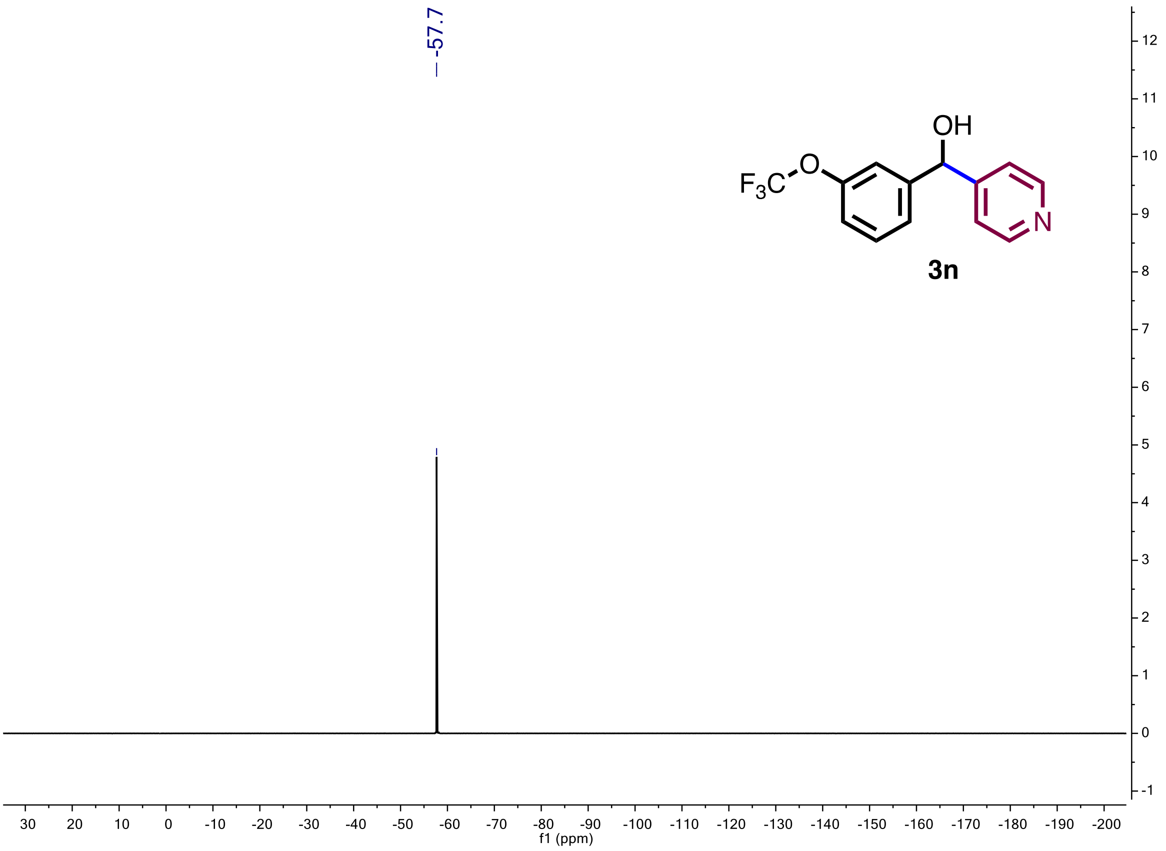
-57.7
OH
F_3C
O
N
3n
f1 (ppm)

^{1}H and ^{13}C NMR spectra of compound **3o** ($CDCl_3$, 400 MHz, 101 MHz)

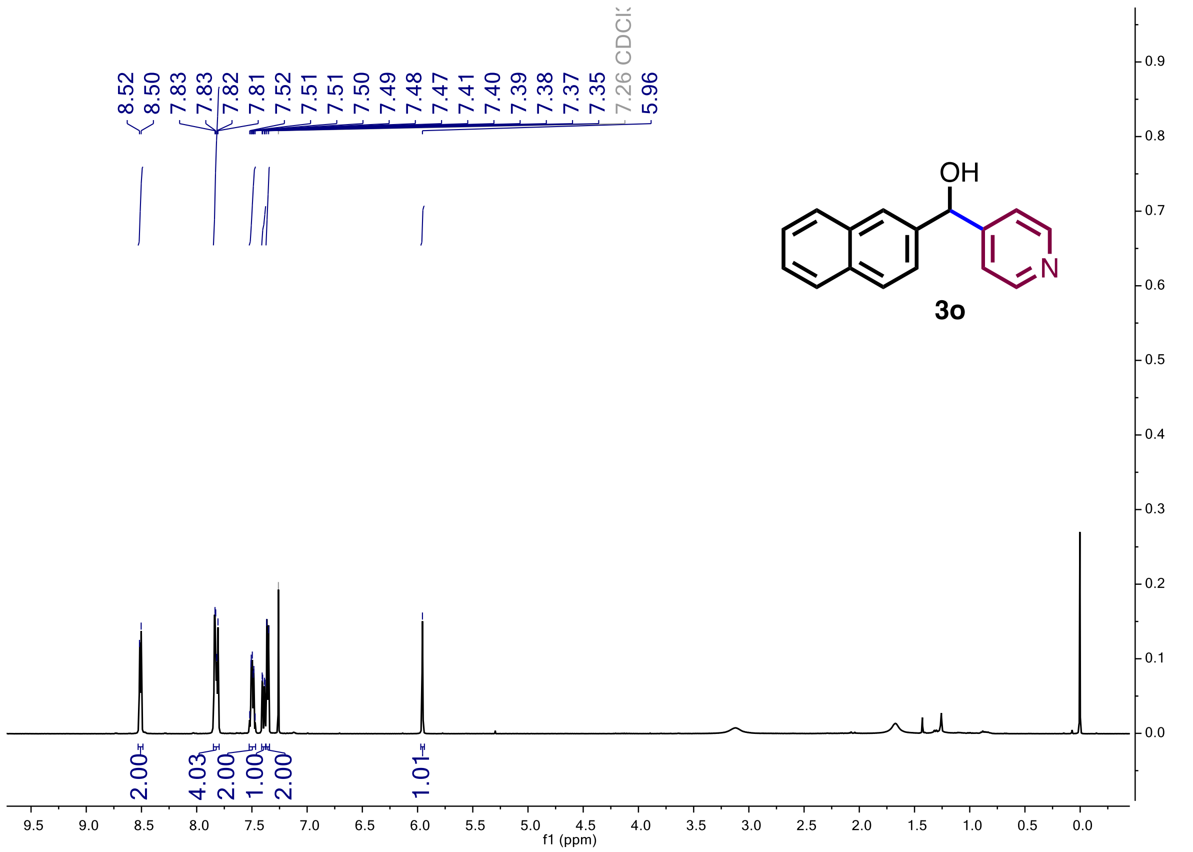

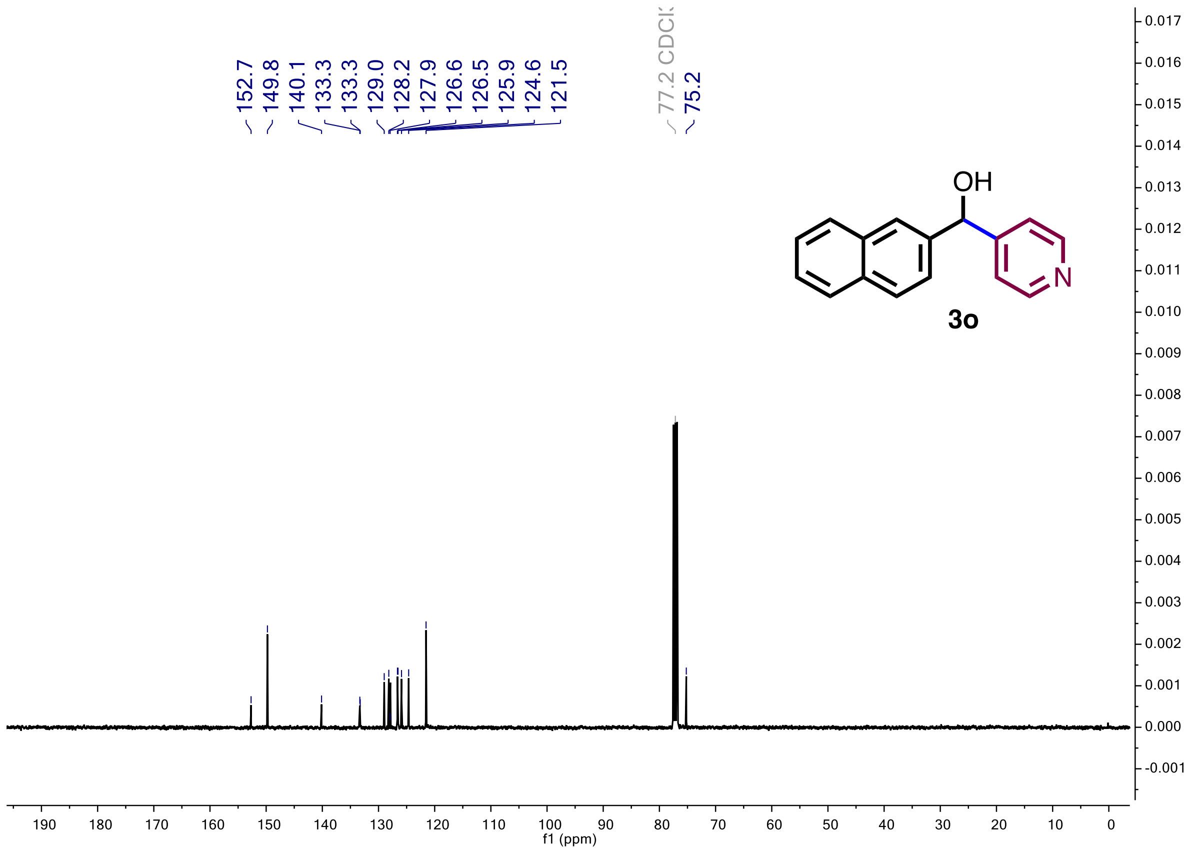

^{1}H and ^{13}C NMR spectra of compound **3p** ($CDCl_3$, 400 MHz, 101 MHz)

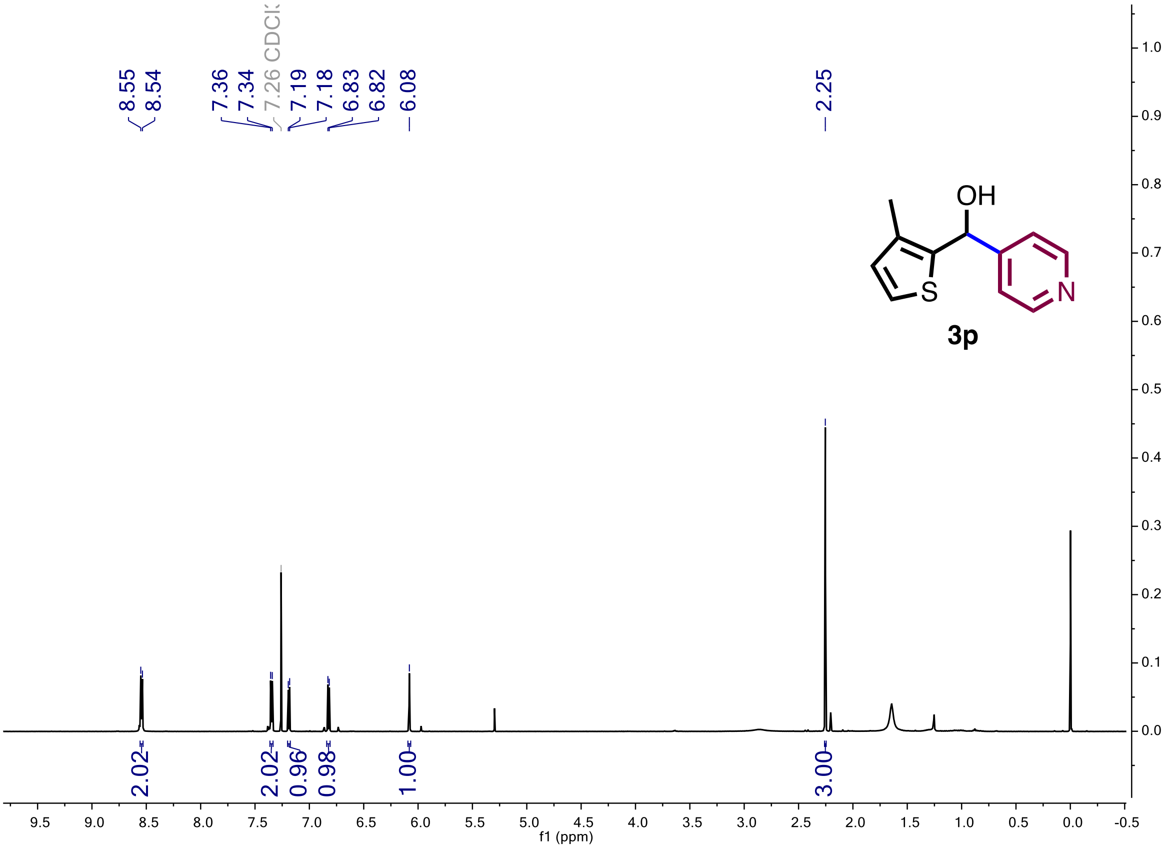

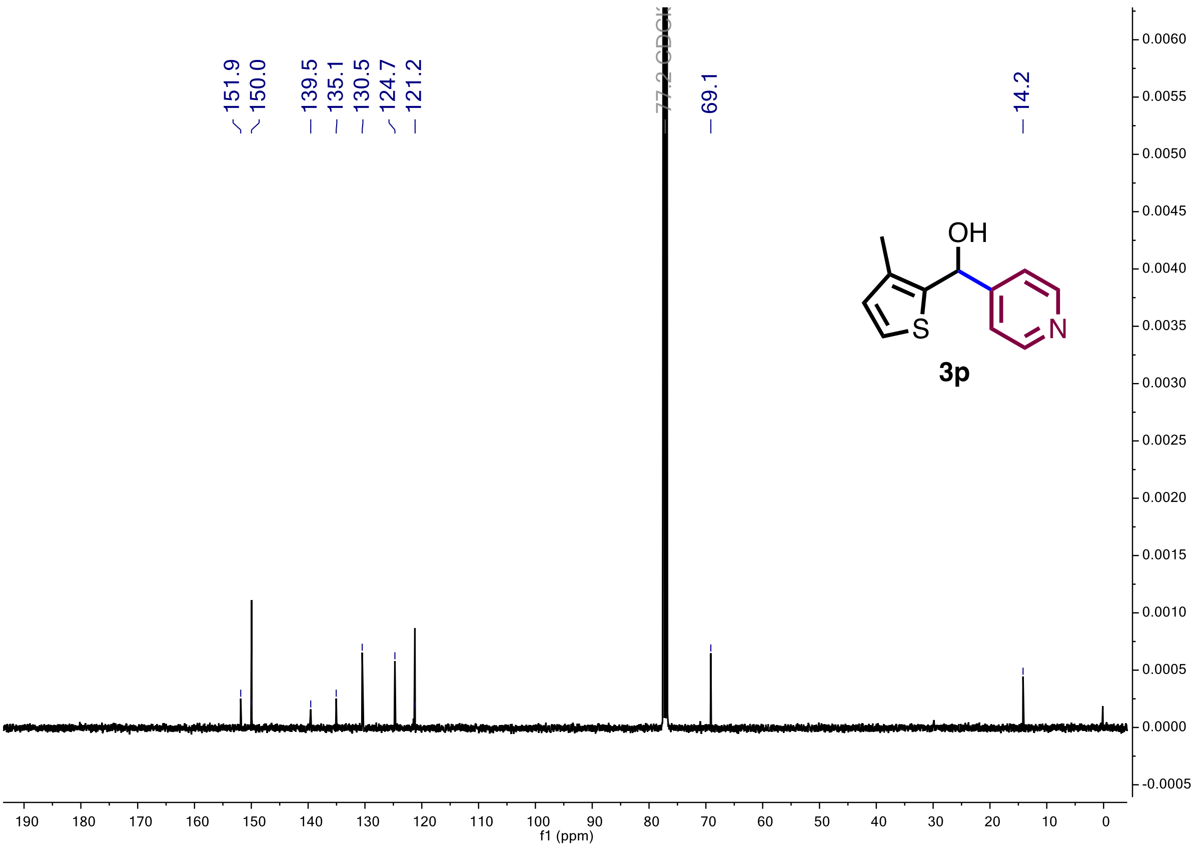

^{1}H and ^{13}C NMR spectra of compound **3q** ($CDCl_3$, 400 MHz, 101 MHz)

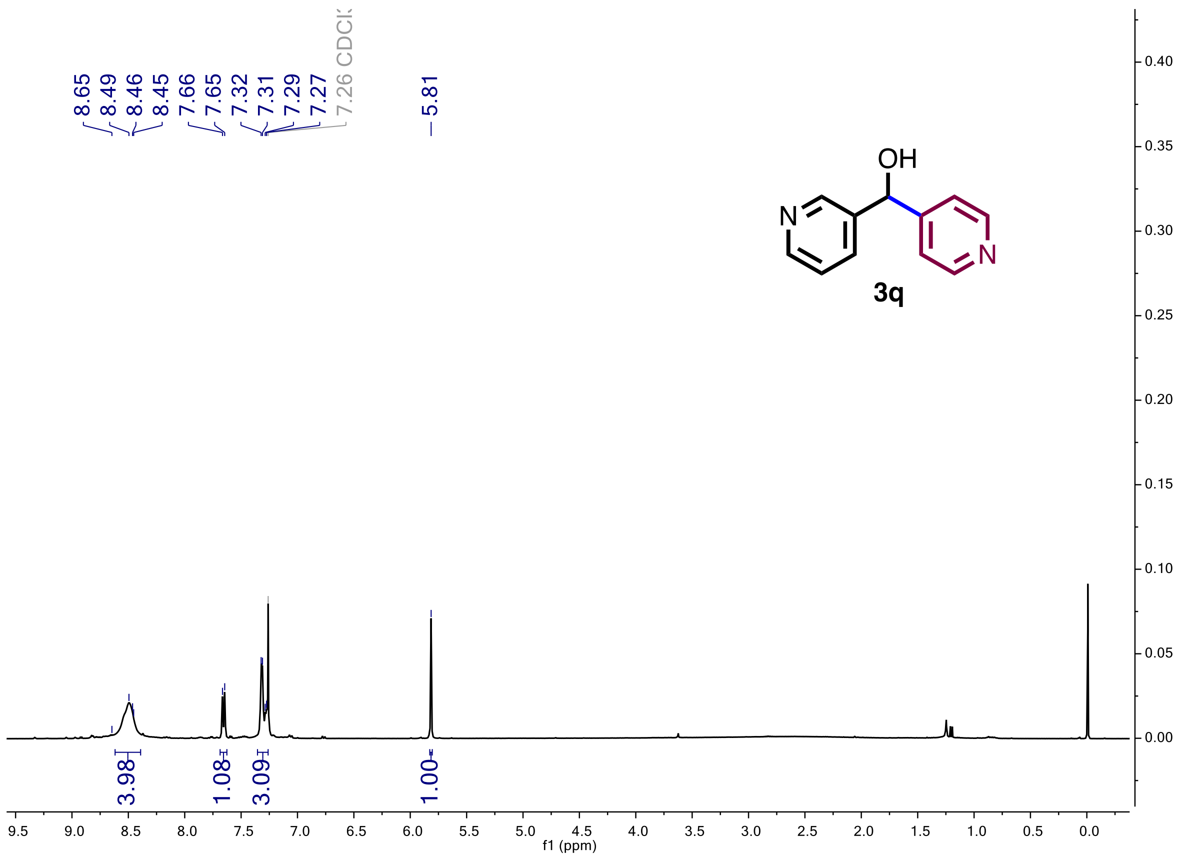

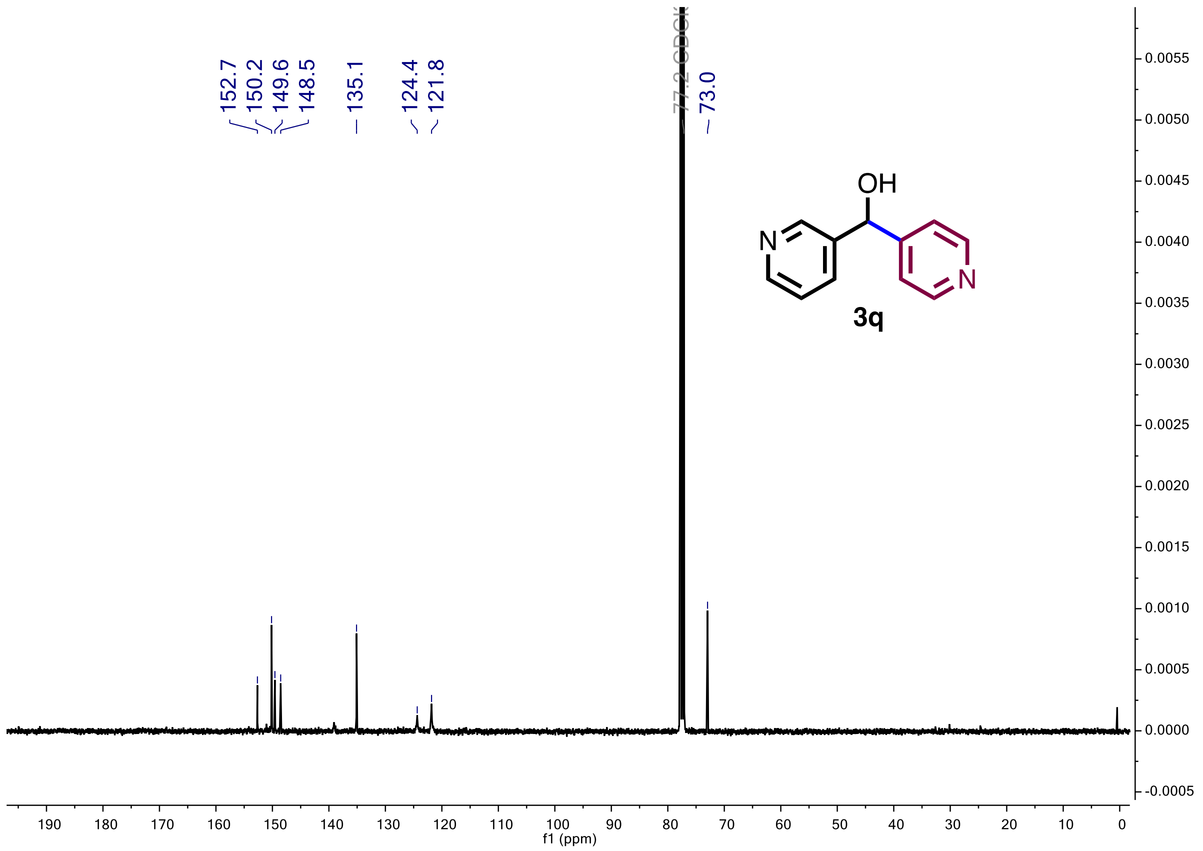

^{1}H and ^{13}C NMR spectra of compound **4a** ($CDCl_3$, 400 MHz, 101 MHz)

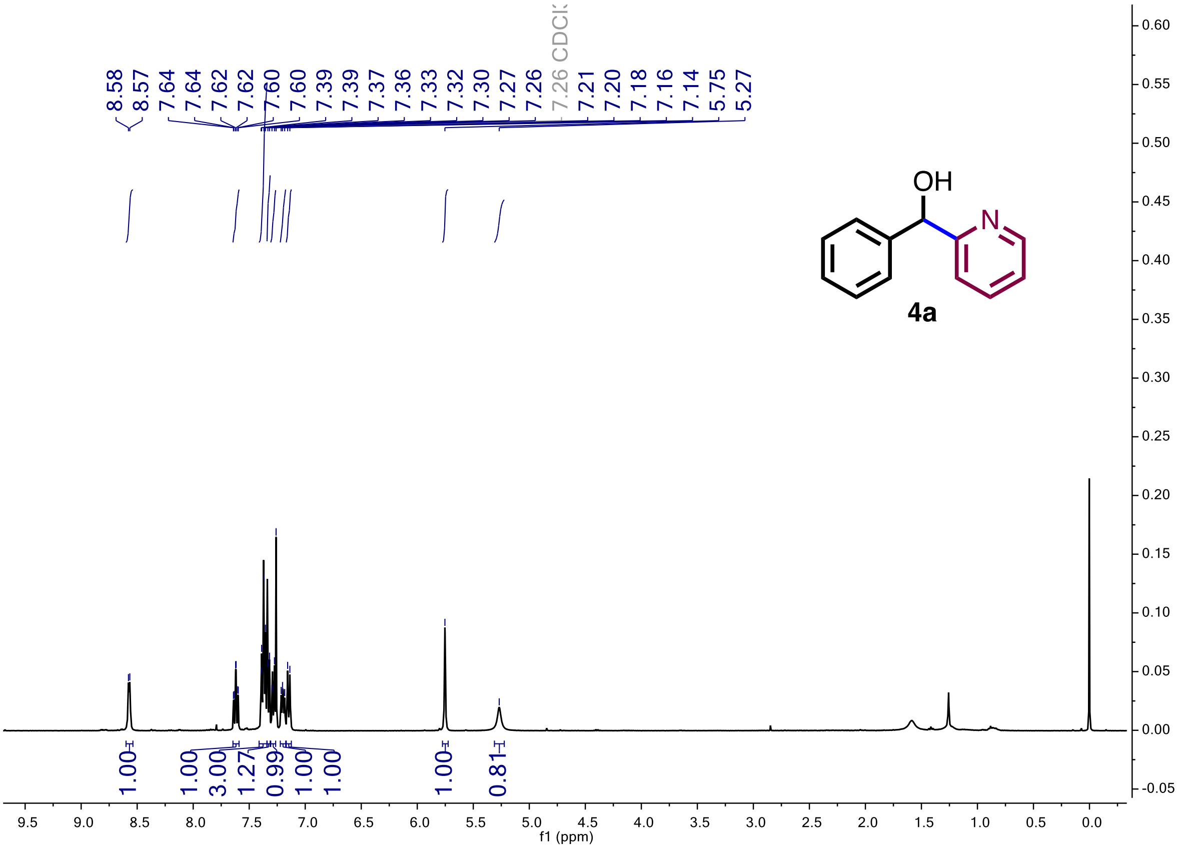

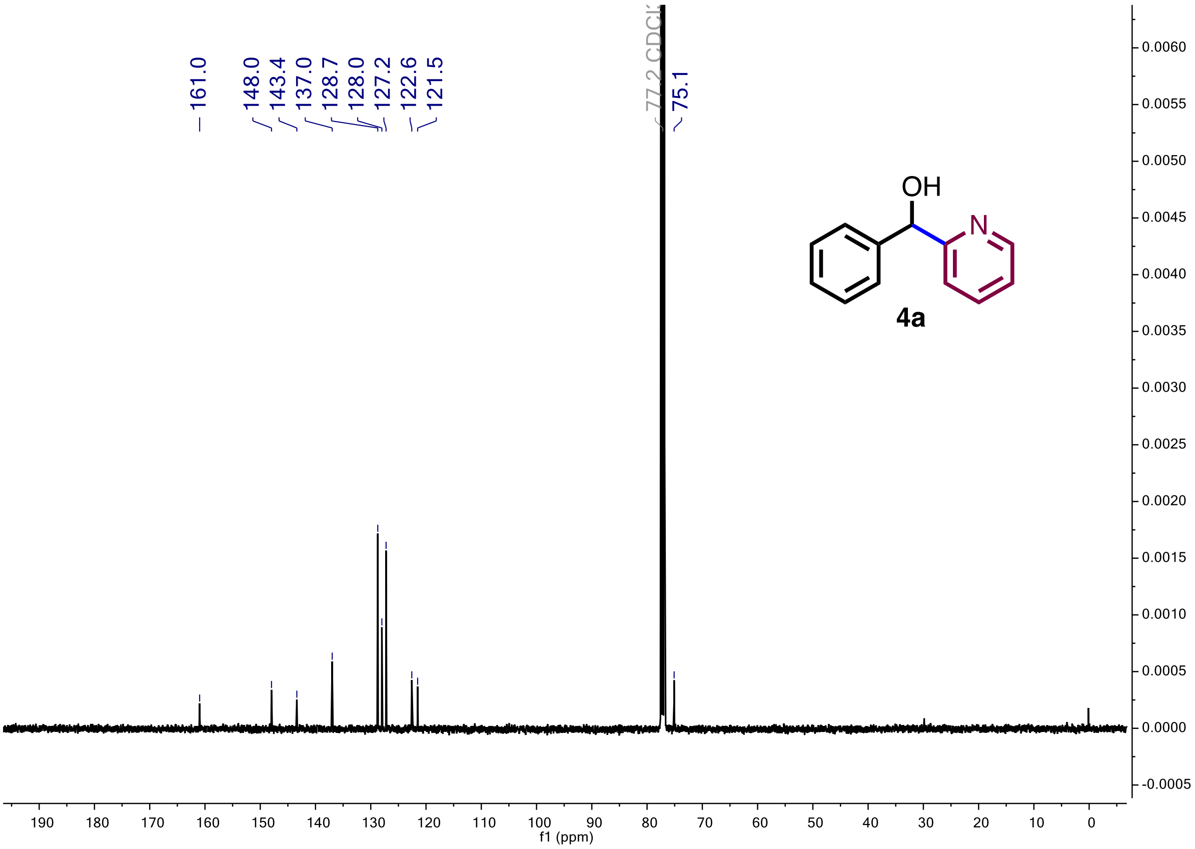

^{1}H and ^{13}C NMR spectra of compound **4b** ($CDCl_3$, 400 MHz, 101 MHz)

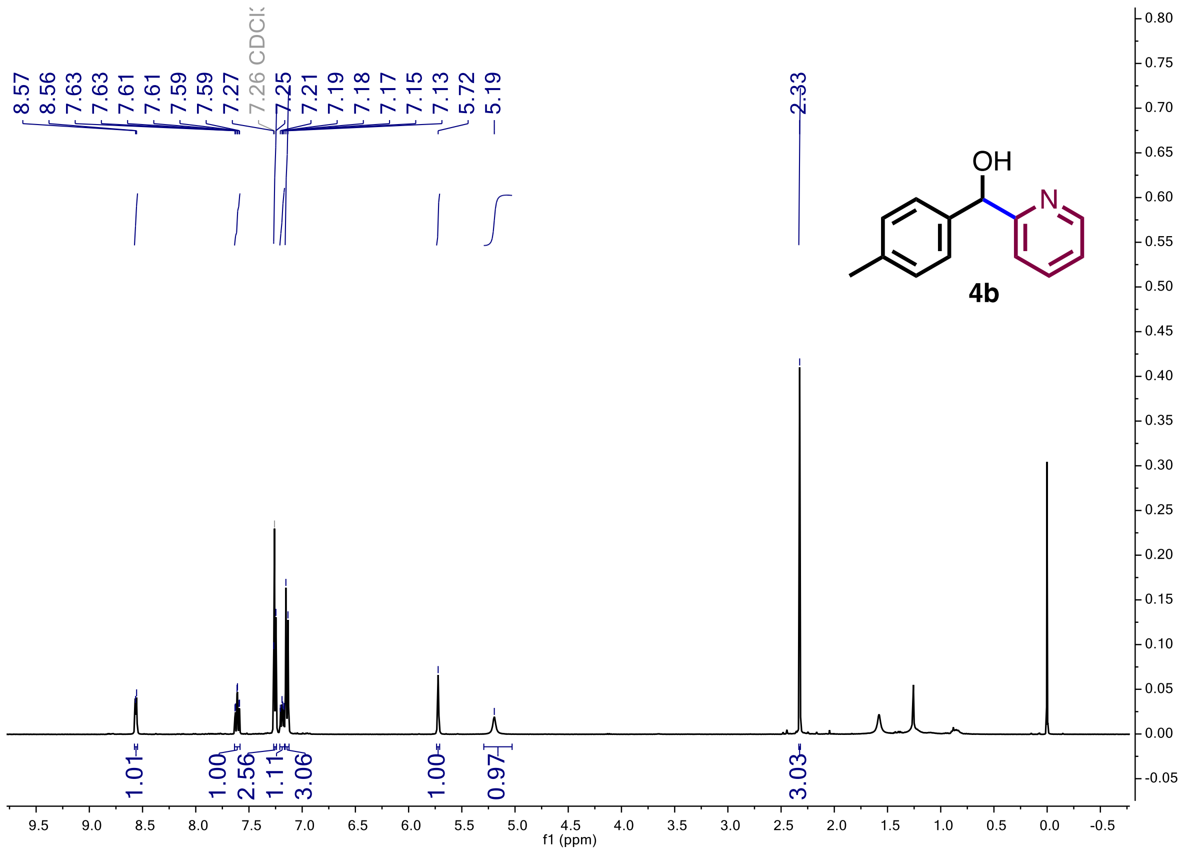

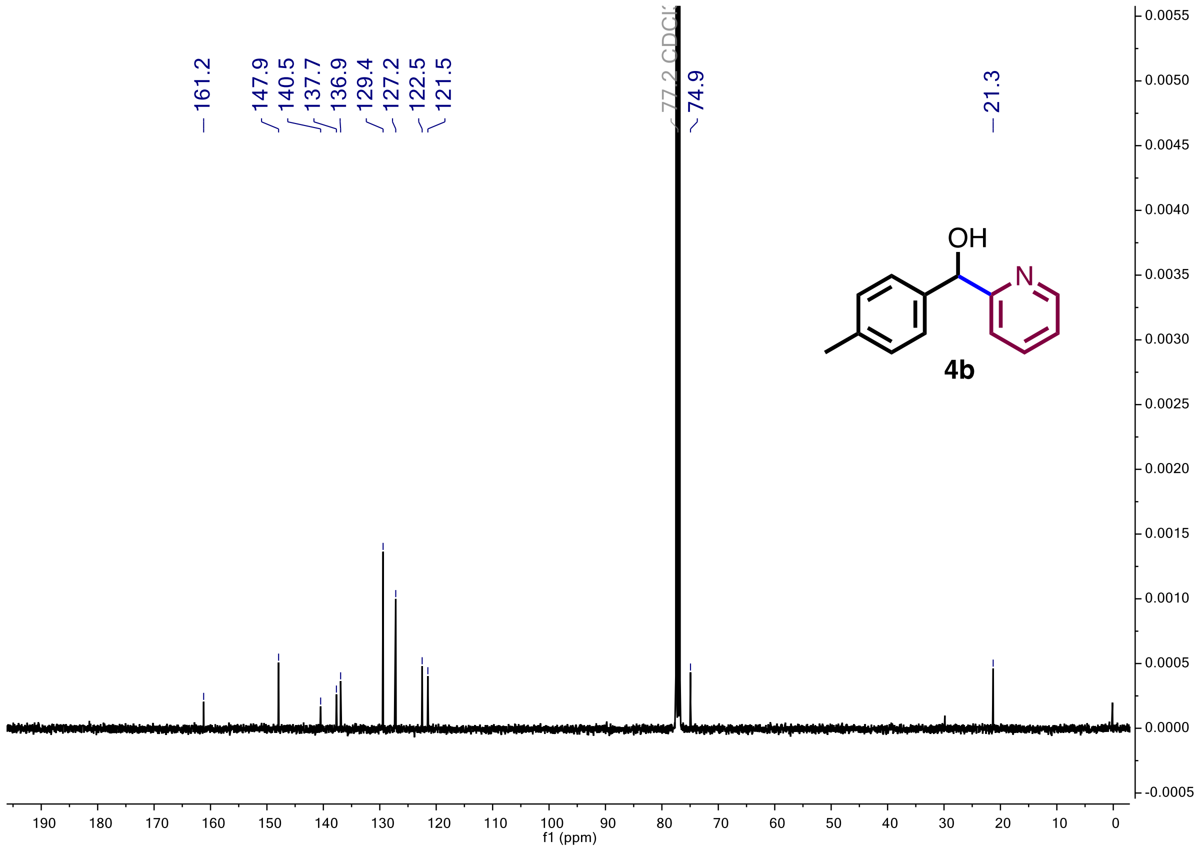

^{1}H and ^{13}C NMR spectra of compound **4c** ($CDCl_3$, 400 MHz, 101 MHz)

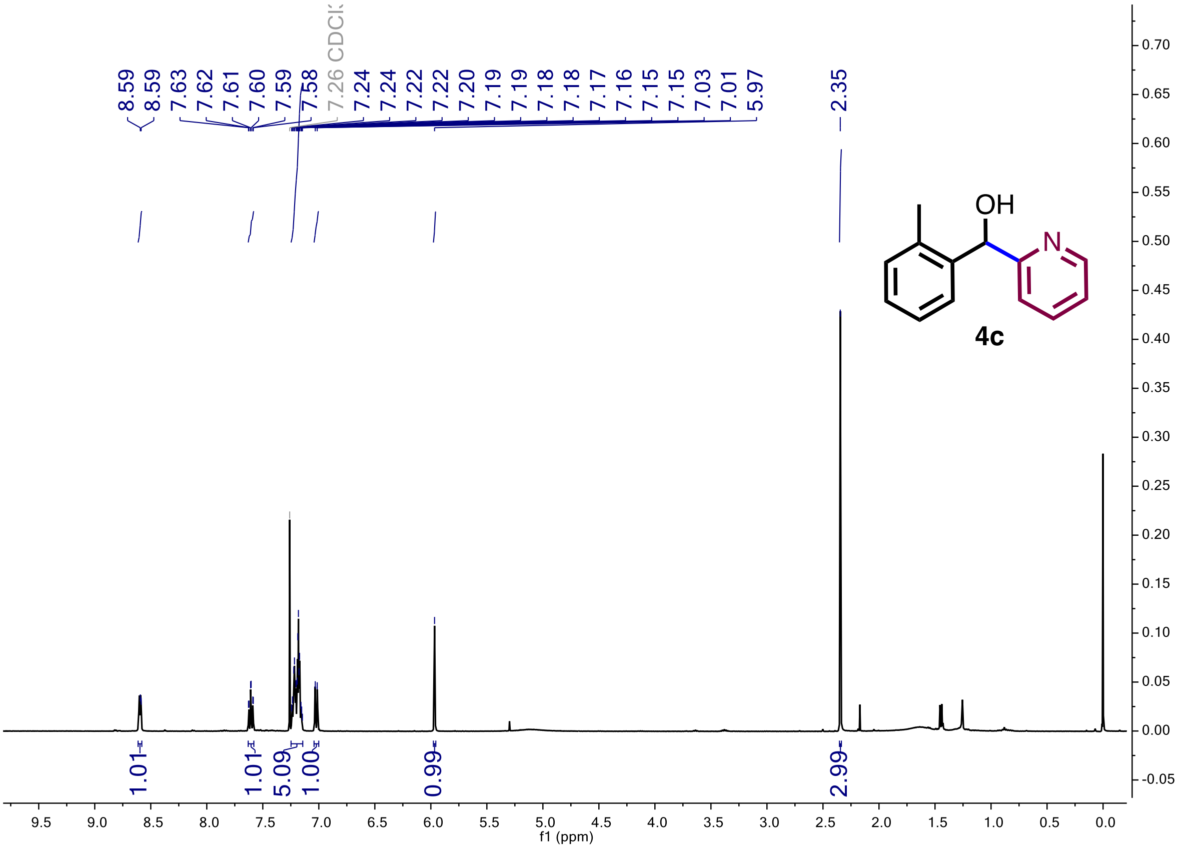

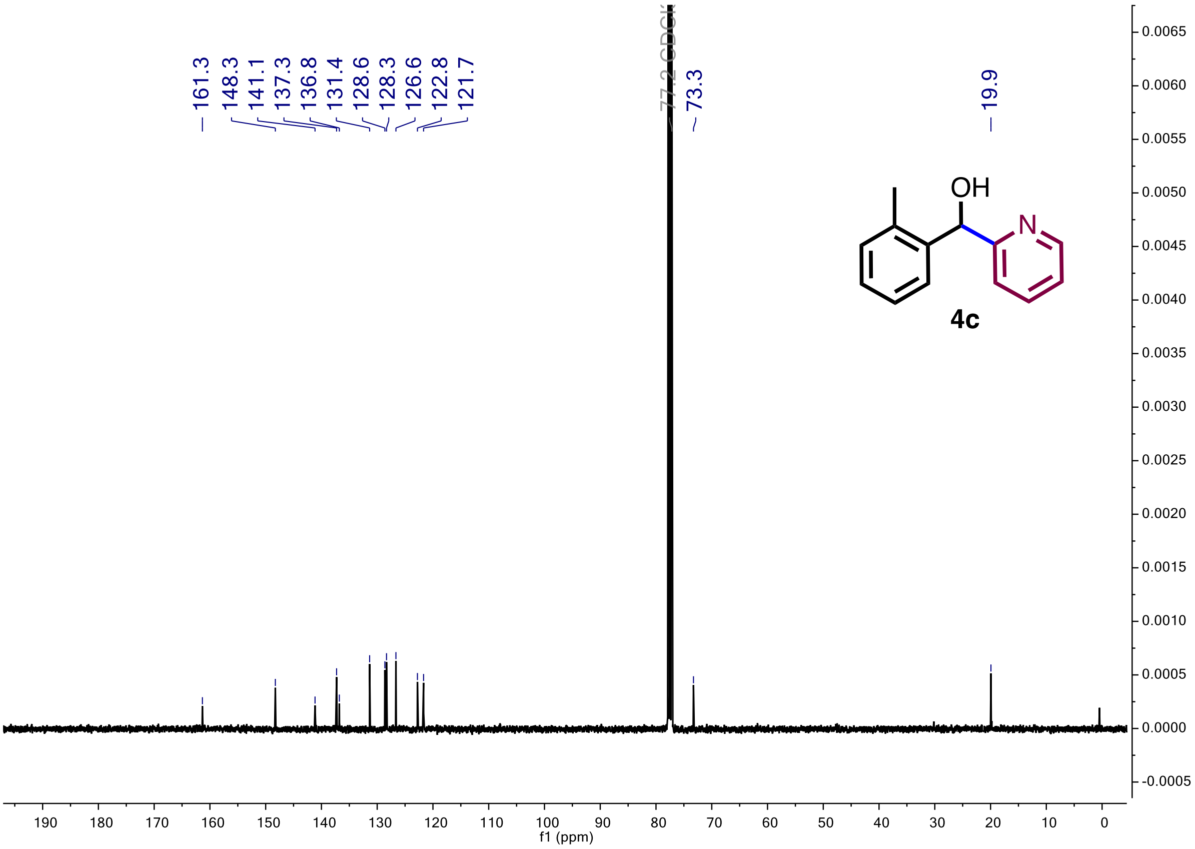

^{1}H, ^{19}F and^{13}C NMR spectra of compound **4d** ($CDCl_3$, 500 MHz, 101 MHz, 376 MHz)

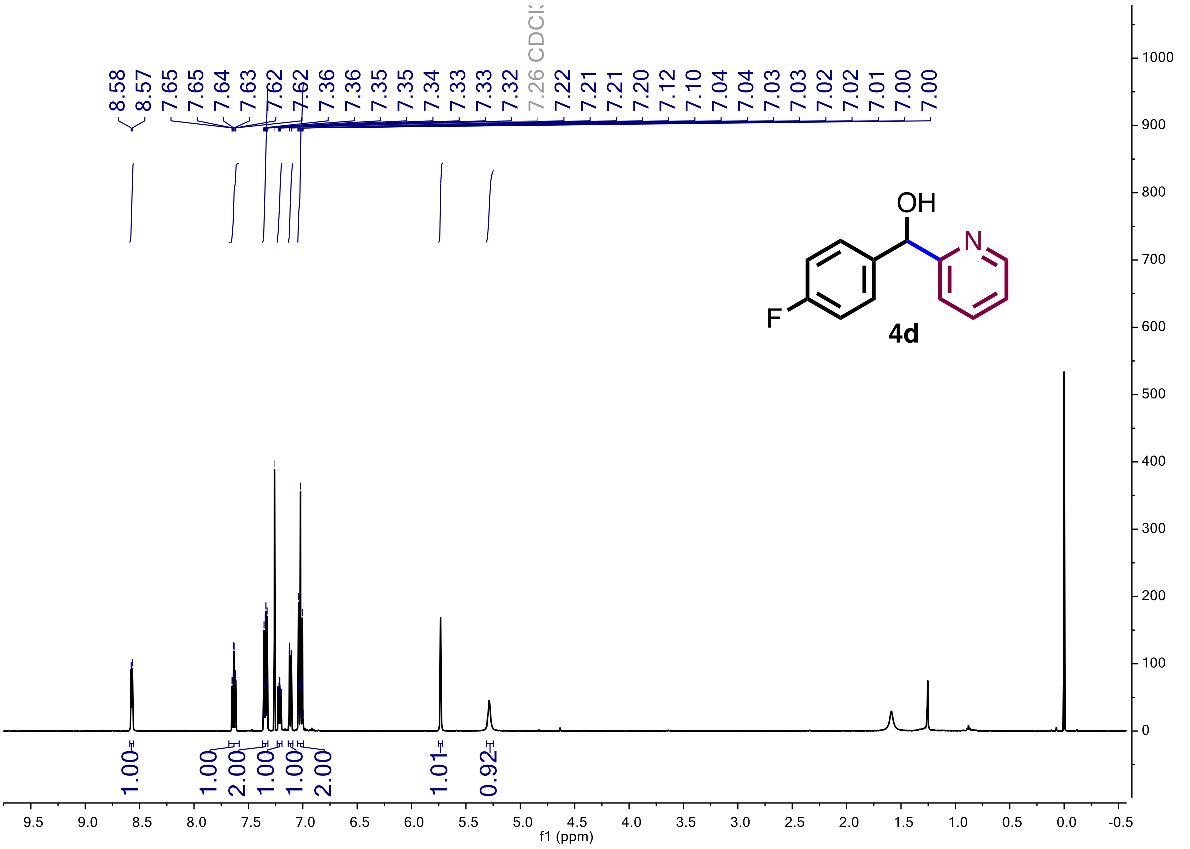

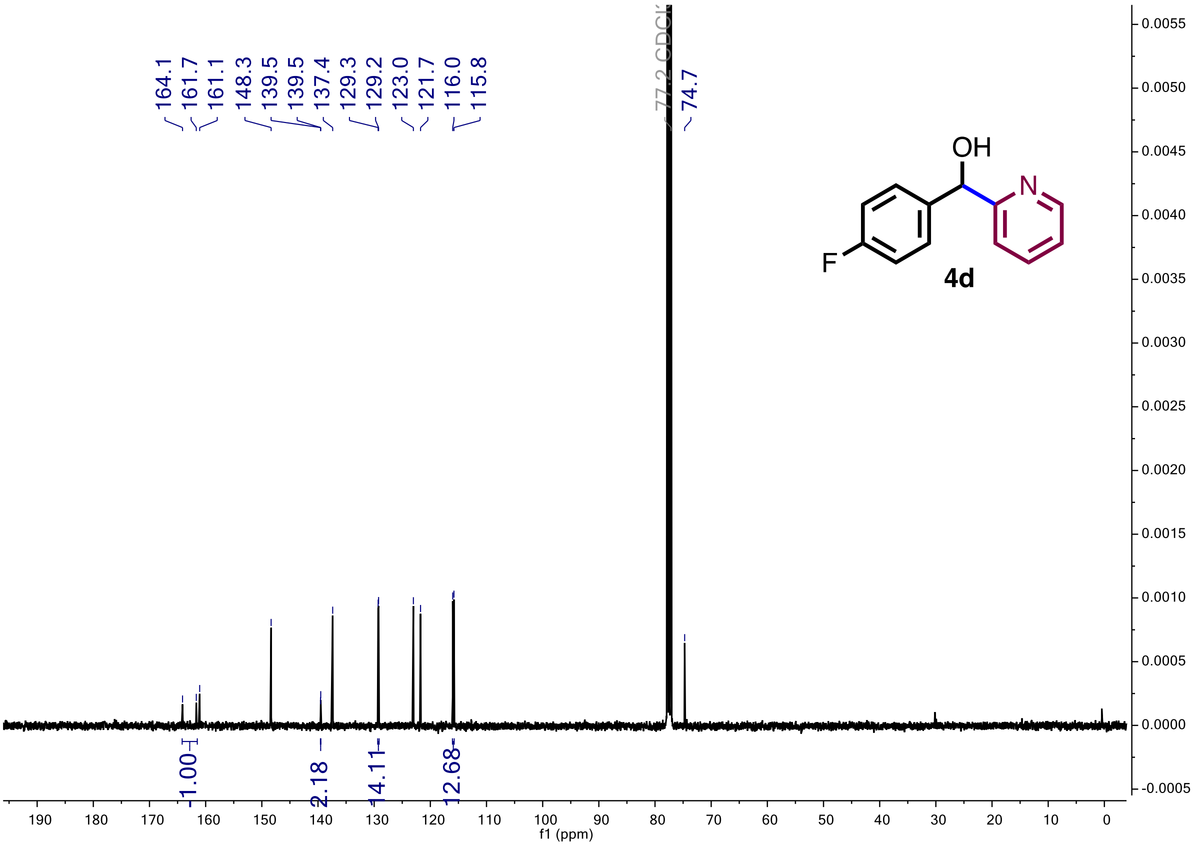

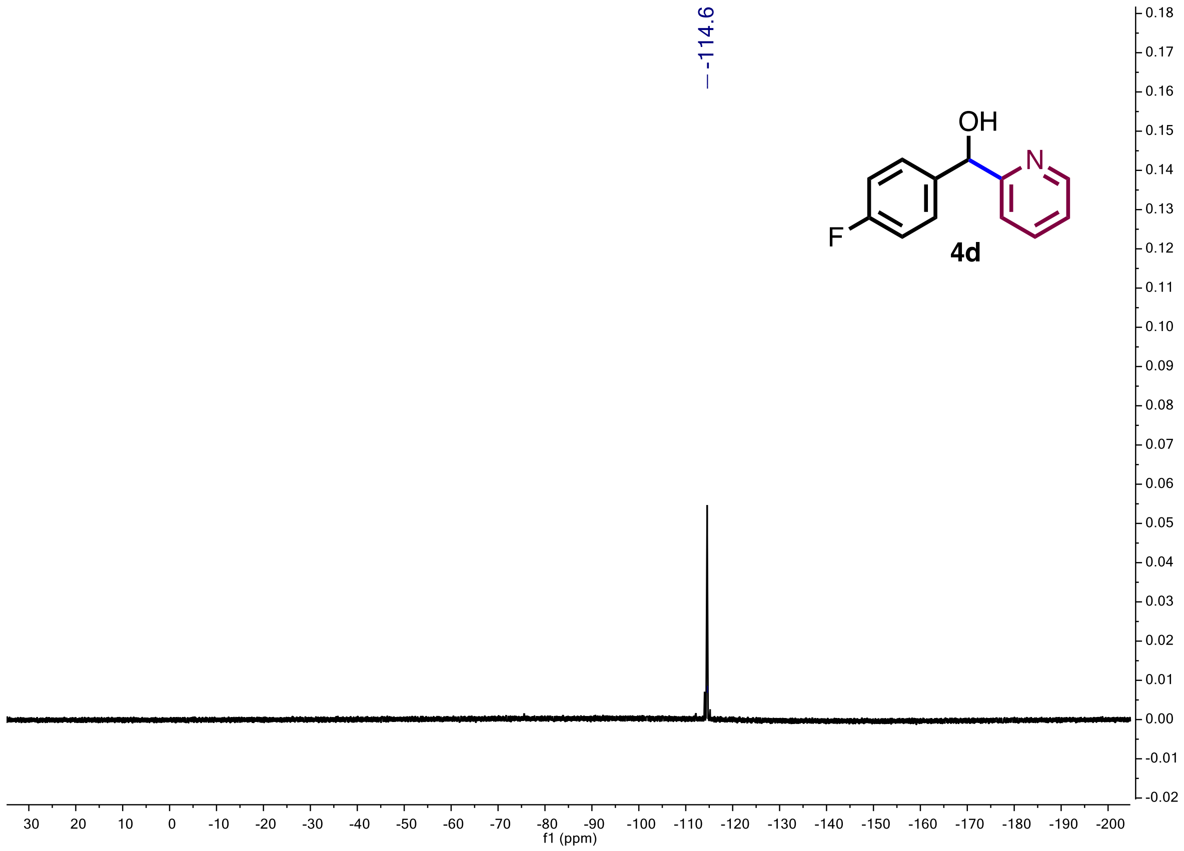

^{1}H and^{13}C NMR spectra of compound **5a** ($CDCl_3$, 400 MHz, 101 MHz)

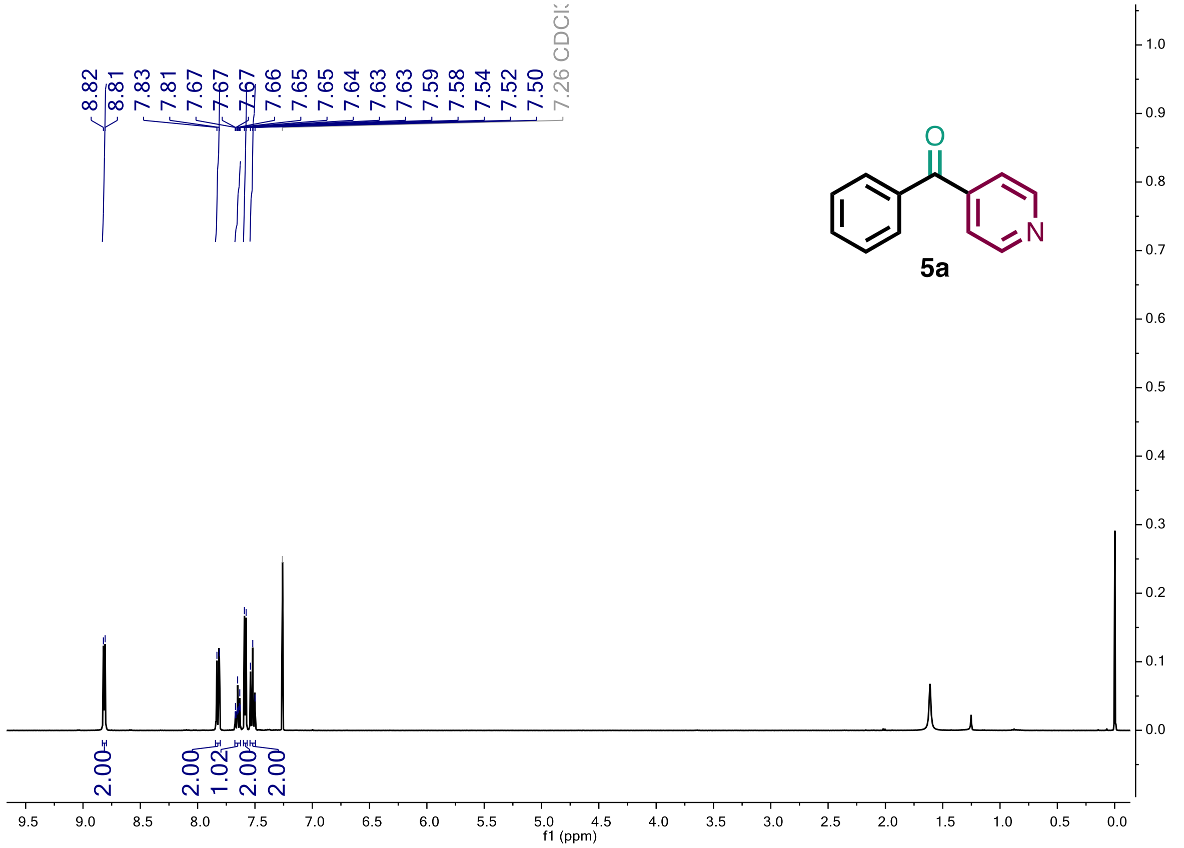

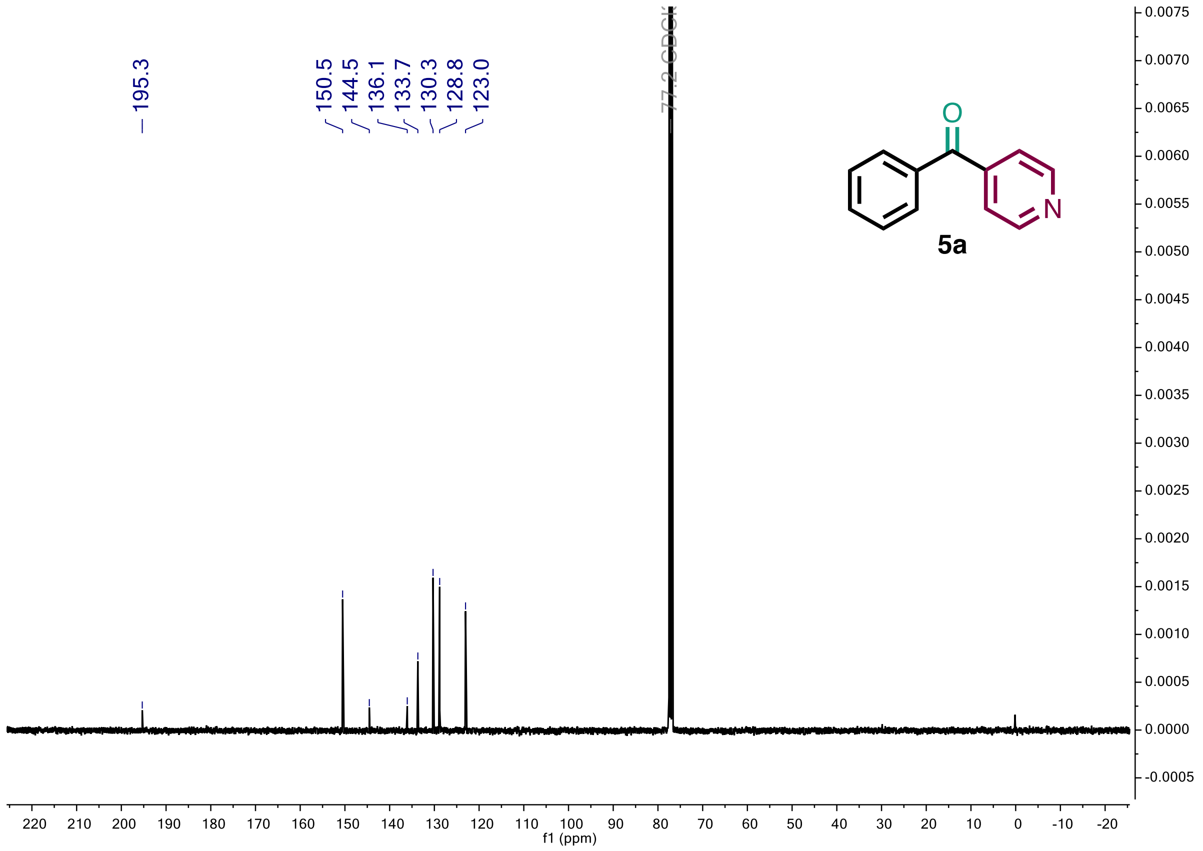

^{1}H and^{13}C NMR spectra of compound **5b** ($CDCl_3$, 400 MHz, 126 MHz)

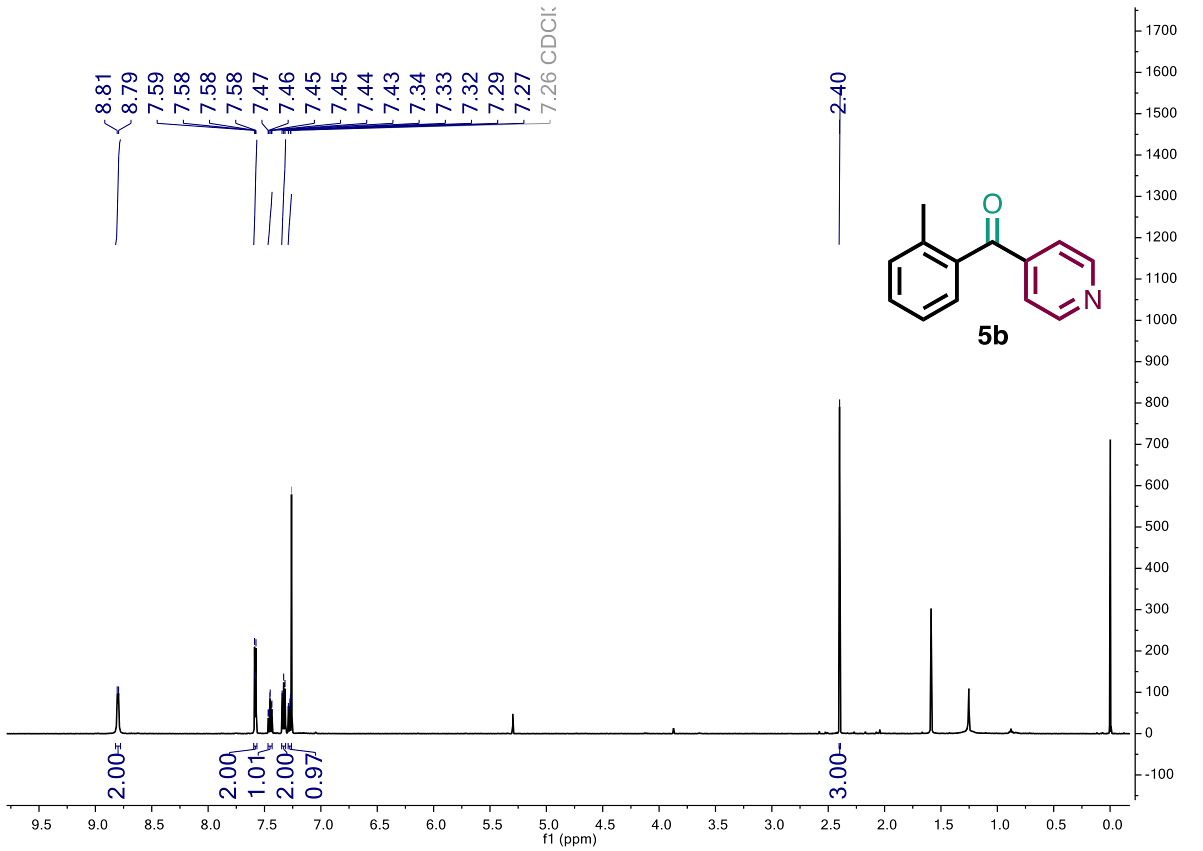

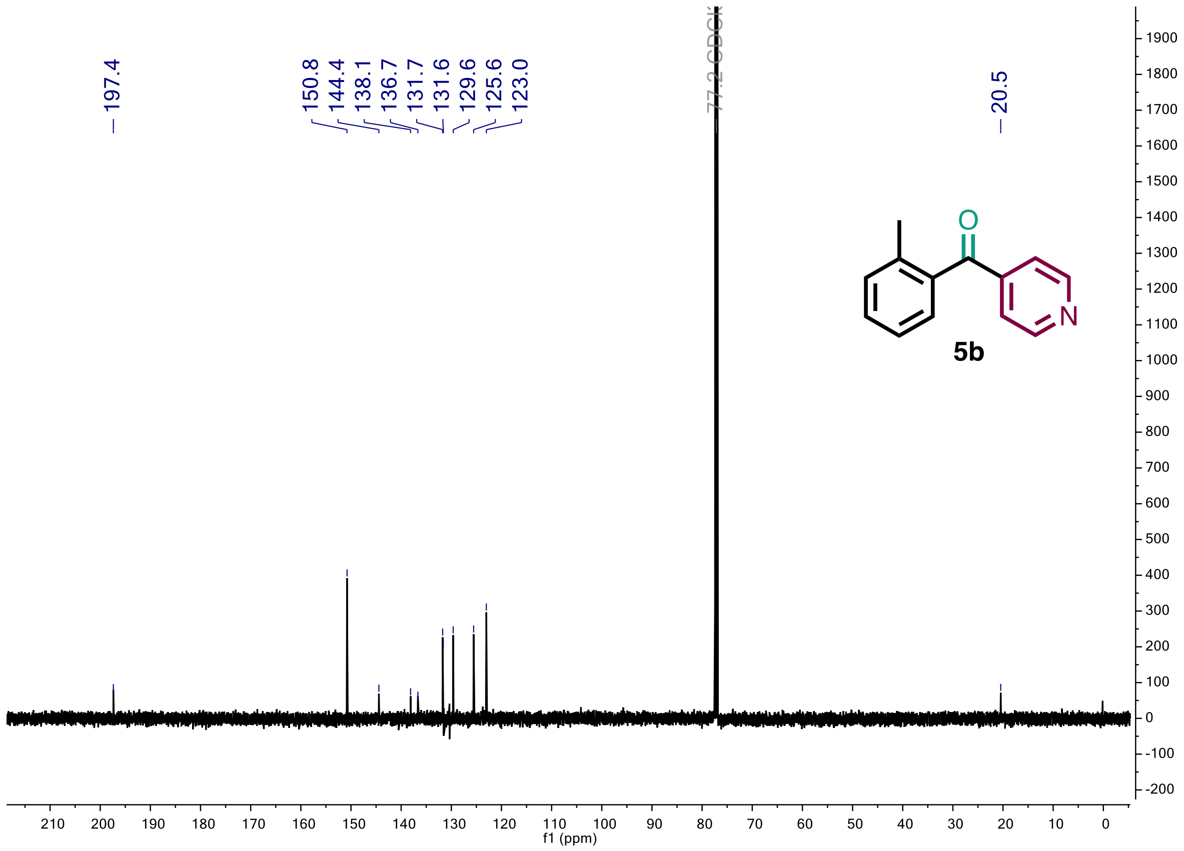

^{1}H and^{13}C NMR spectra of compound **5c** ($CDCl_3$, 400 MHz, 126 MHz)

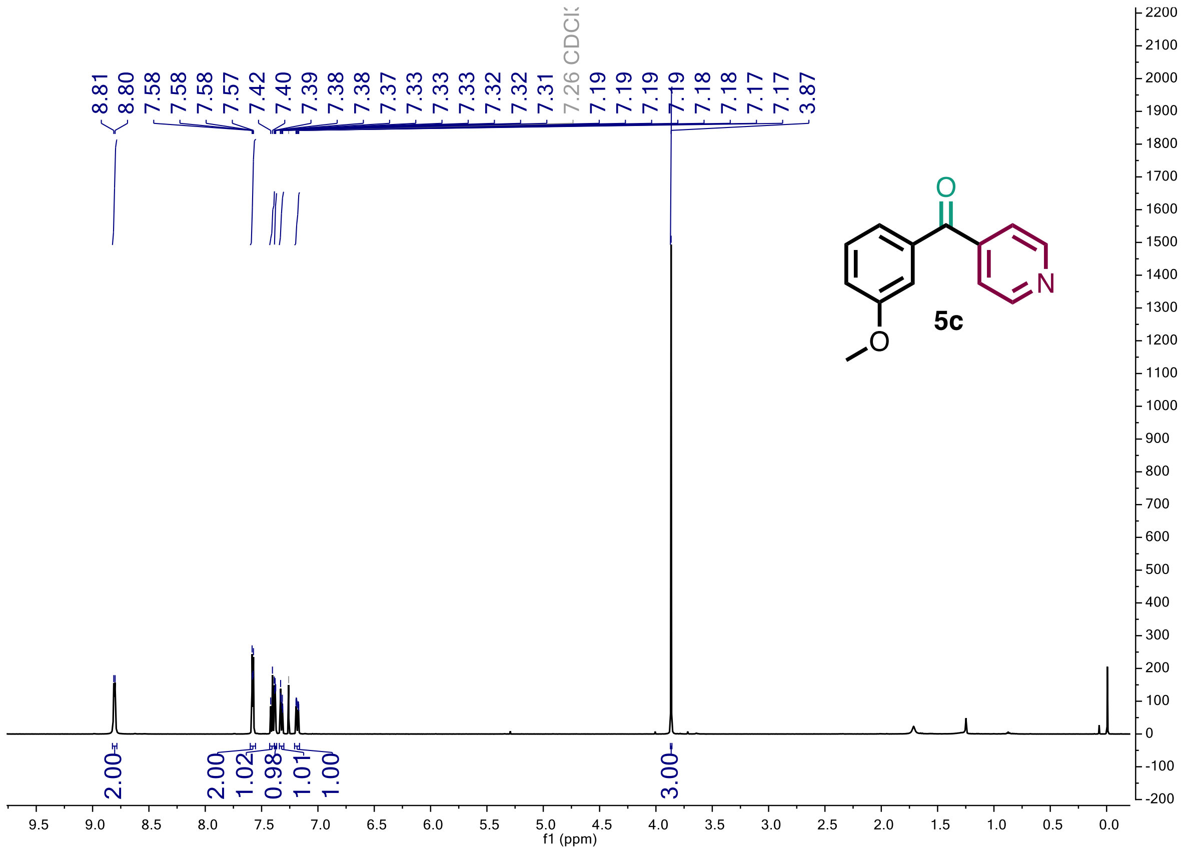

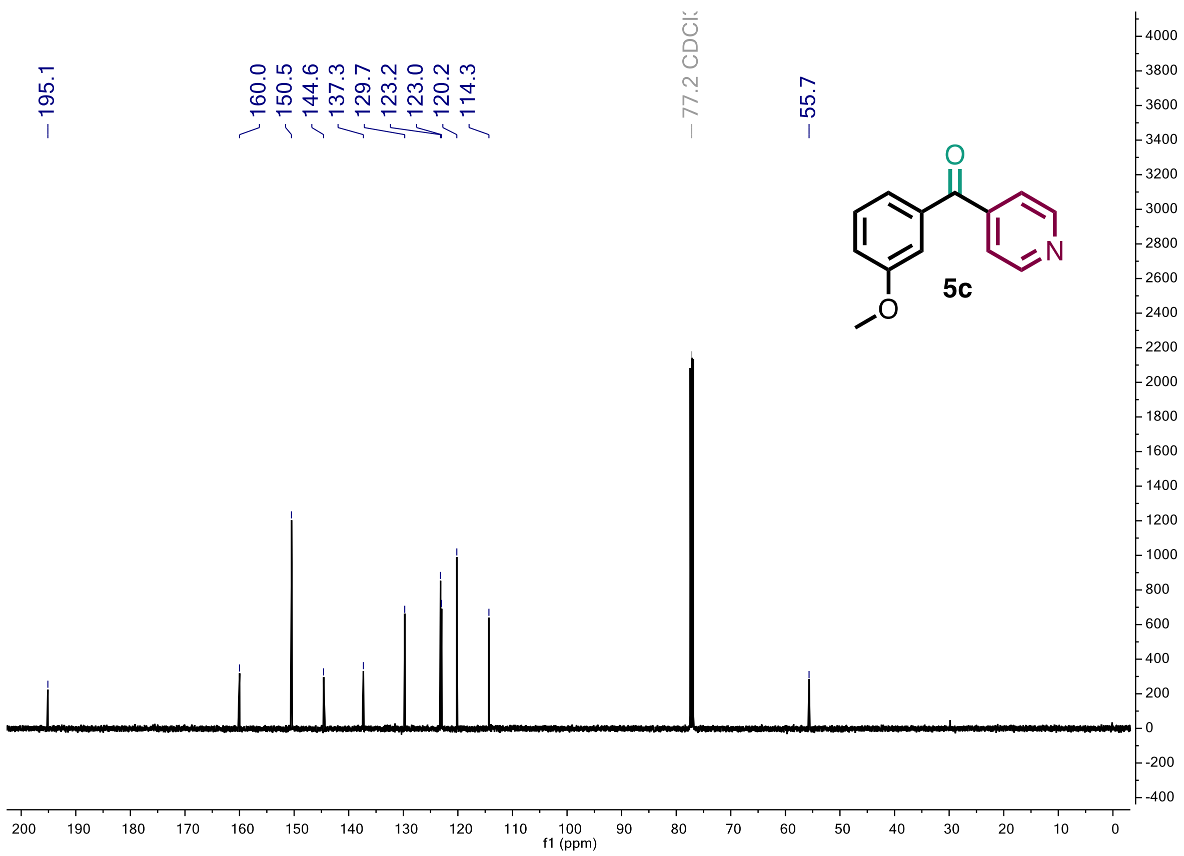

^{1}H and^{13}C NMR spectra of compound **5d** ($CDCl_3$, 400 MHz, 126 MHz)

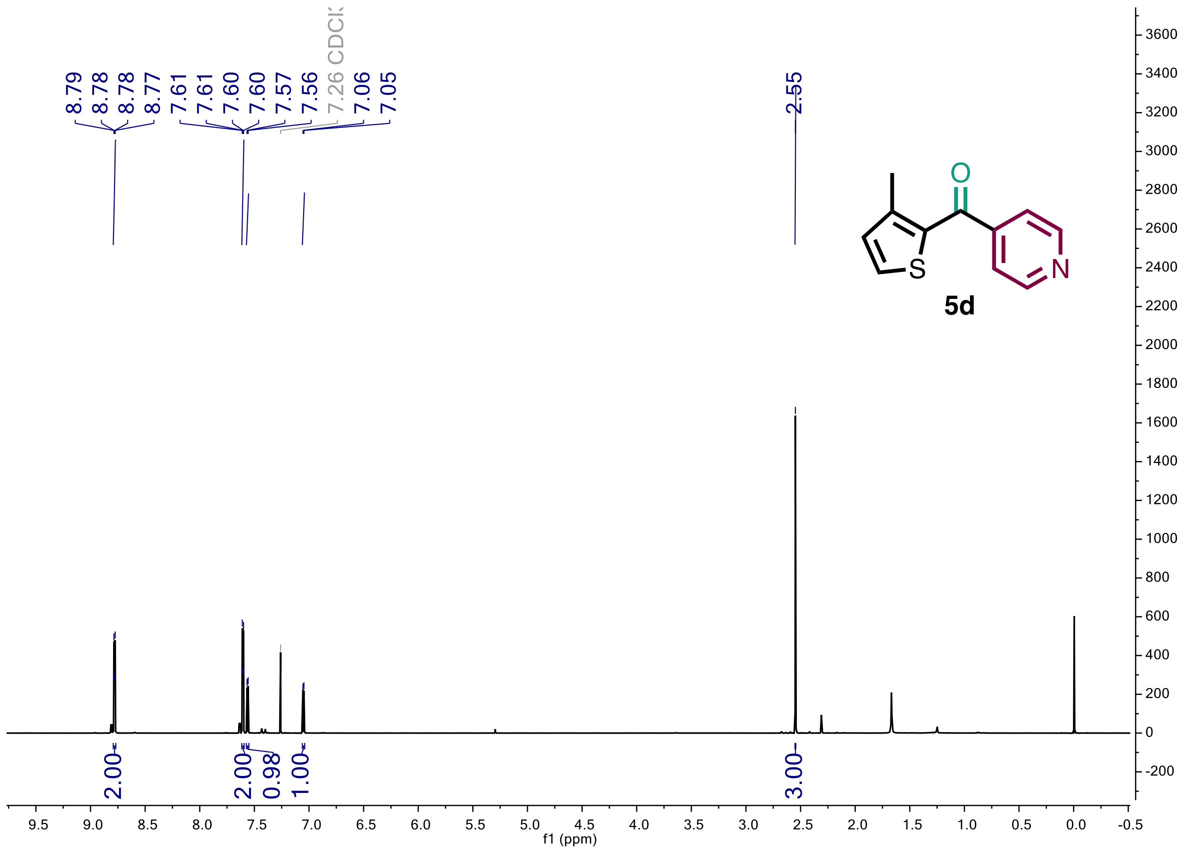

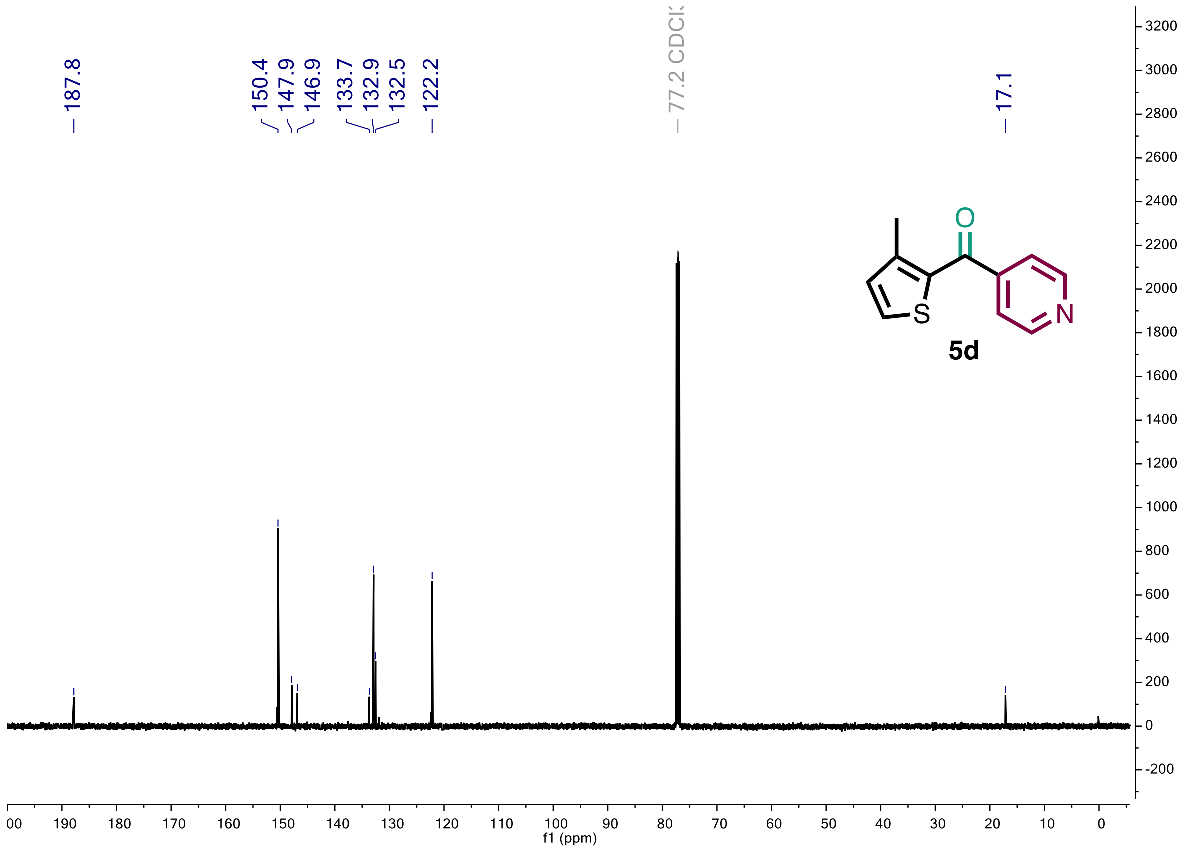

^{1}H and ^{13}C NMR spectra of compound **5e** ($CDCl_3$, 400 MHz, 126 MHz)

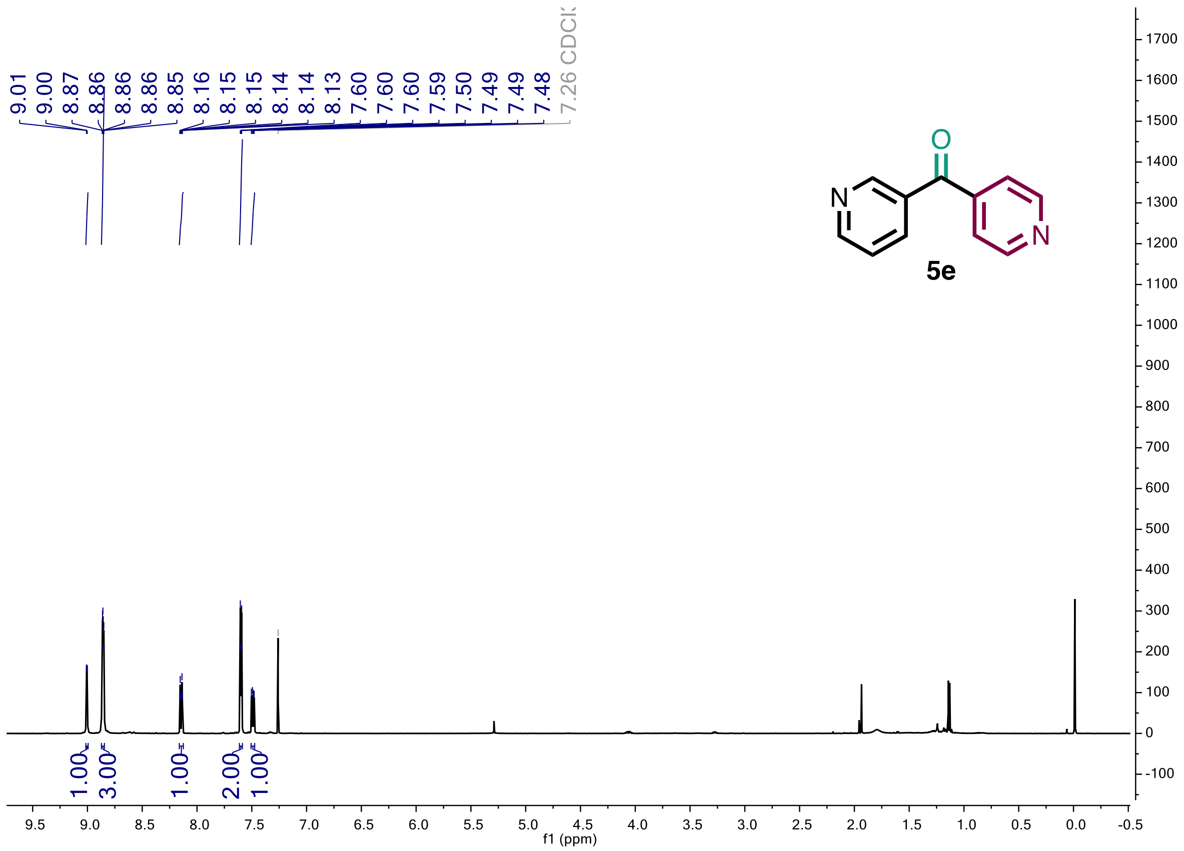

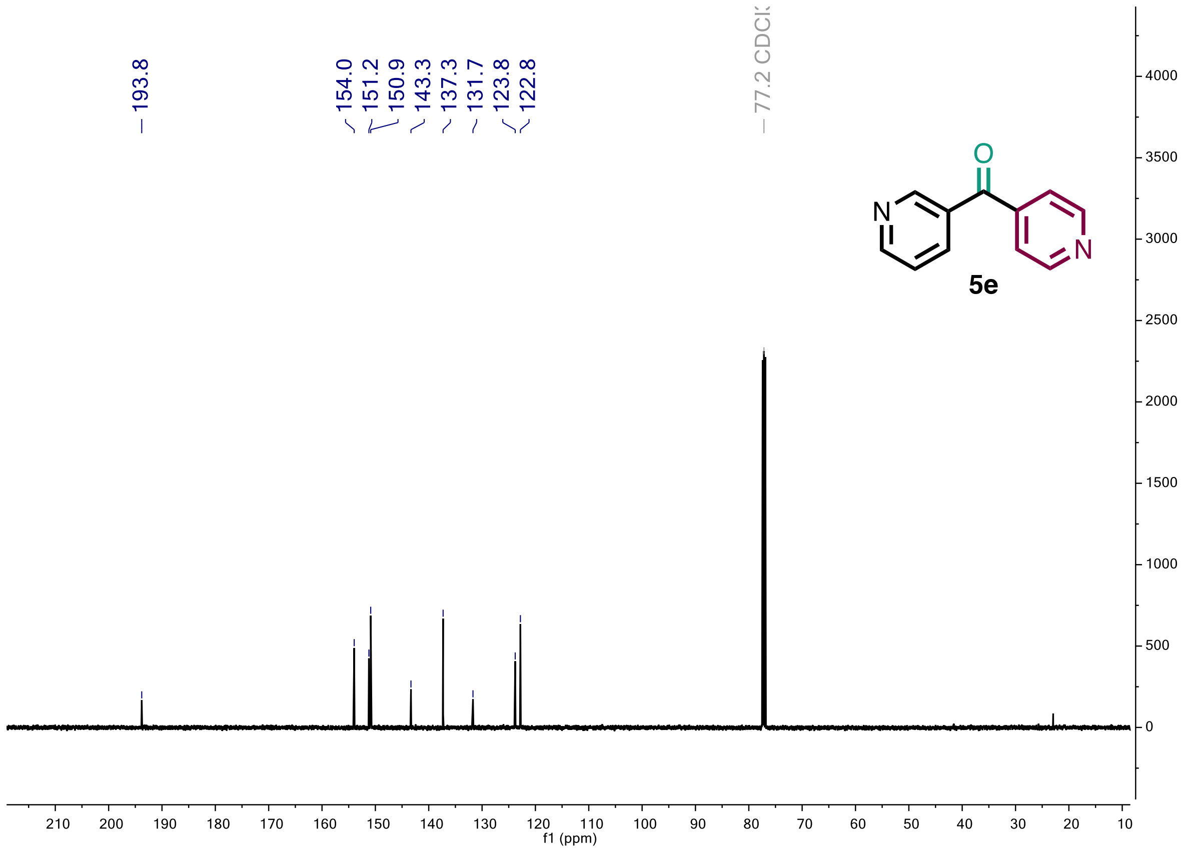
193.8
154.0
151.2
150.9
143.3
137.3
131.7
123.8
122.8
77.2 CDCl3
O
N
N
5e
4000
3500
3000
2500
2000
1500
1000
500
0
210 200 190 180 170 160 150 140 130 120 110 100 90 80 70 60 50 40 30 20 10
f1 (ppm)
